# Pressure-induced liquid-liquid transition in a family of ionic materials

**DOI:** 10.1038/s41467-022-29021-0

**Published:** 2022-03-15

**Authors:** Zaneta Wojnarowska, Shinian Cheng, Beibei Yao, Malgorzata Swadzba-Kwasny, Shannon McLaughlin, Anne McGrogan, Yoan Delavoux, Marian Paluch

**Affiliations:** 1 Institute of Physics, the University of Silesia in Katowice, Silesian Center for Education and Interdisciplinary Research, 75 Pulku Piechoty 1A, 41–500 Chorzow, Poland; 2 Department of Chemistry, University of Wisconsin-Madison, Madison, WI 53705 USA; 3 The QUILL Research Centre, School of Chemistry and Chemical Engineering, The Queen’s University of Belfast, David Keir Building, Stranmillis Rd, BT9 5AG Belfast, NI UK

**Keywords:** Ionic liquids, Thermodynamics, Phase transitions and critical phenomena

## Abstract

Liquid−liquid transition (LLT) between two disordered phases of single-component material remains one of the most intriguing physical phenomena. Here, we report a first-order LLT in a series of ionic liquids containing trihexyl(tetradecyl)phosphonium cation [P_666,14_]^+^ and anions of different sizes and shapes, providing an insight into the structure-property relationships governing LLT. In addition to calorimetric proof of LLT, we report that ion dynamics exhibit anomalous behavior during the LLT, i.e., the conductivity relaxation times (*τ*_*σ*_) are dramatically elongated, and their distribution becomes broader. This peculiar behavior is induced by isobaric cooling and isothermal compression, with the *τ*_*σ*_(*T*_LL_,*P*_LL_) constant for a given system. The latter observation proves that LLT, in analogy to liquid-glass transition, has an isochronal character. Finally, the magnitude of discontinuity in a specific volume at LLT was estimated using the Clausius-Clapeyron equation.

## Abstract (web summary)

Liquid−liquid transitions (LLTs) have been reported for some molecular systems but are difficult to observe under high pressure conditions. Here the authors report and characterize a first-order LLT in a series of ionic liquids containing the trihexyl(tetradecyl)phosphonium cation and anions of different sizes and shapes, using calorimetric and dielectric measurements.

## Introduction

When isotropic liquid is cooled below the melting point, it either solidifies into a crystal or enters into a metastable supercooled state, which then enters a non-equilibrium amorphous phase at the glass transition temperature *T*_g_^1^. The characteristic feature of the latter transformation is a continuous increase of density, accompanied by a slowing down of molecular dynamics and enormous elongation of structural relaxation times: from the time scale of picoseconds at *T*_m_ up to hundreds of seconds in the vicinity of *T*_g_^2^. If cooled rapidly enough, nearly all materials can be transformed into an amorphous form. Thus, the glass-forming ability can be considered as a universal property of condensed matter.

Over the years, such a well-established physical picture of the liquid state has been upended by numerous examples of two distinct liquid phases in single-component materials. The first-order liquid–liquid transition (LLT), separating fluids of different local structures, density, magnetic susceptibility and thermodynamic properties, has been reported for various systems, including atomic elements (sulfur, phosphorus^3^, silicon^4,5^, carbon^6^ and strongly interacting liquids^7^, such as molten oxides^8,9^, e.g., Al_2_O_3_-Y_2_O_3_^10^. Only four molecular liquids (water^11,12^, triphenyl phosphite (TPP)^13–15^, n-butanol^16^, and D-mannitol^17^ have been found with compelling evidence for LLT. Nevertheless, the LLT in these systems remains controversial since it occurs in the supercooled state capable of cold crystallization^18^. The theoretical and experimental observations show that a first-order LLT can occur without a noticeable density change, making this phenomenon even more puzzling^19^. Furthermore, magnetic field can affect the local structure and lead to another liquid phase^20^. It was reported that an aligned liquid state of Co alloys can be transformed into another liquid phase under magnetic fields^21,22^. At the same time, except for a few cases^6,7,23,24^ not much is known about the effect of molecular packing on LLT. Consequently, it has not been clarified how universal the LLT is and what is the critical factor inducing such a transition. Since it has been difficult to identify other examples of LLT in a systematic fashion, the experimental verification of these problems poses a great challenge.

Aprotic ionic liquids (AILs)—a class of glass-formers composed solely of ions^25^, give a unique opportunity to investigate the universality of LLT. The most interesting feature of AILs is that their structural and transport properties can be finely tuned within a wide range by the combination of different positively and negatively charged ions^26^. Thereby, a vast structural diversity of ionic species and various types of competing intermolecular interactions (van der Waals, H-bonding and Coulomb forces), make AILs excellent materials to probe the mechanism underpinning LLT. Furthermore, several previous studies show unique ordering behavior of ionic liquid, namely formation of nanoscale domains of polar and nonpolar groups^27–29^, that potentially can lead to LLT. However, over the years there was no proof of LLT in ILs capable for domains formation. The first report on LLT in ionic liquid has been provided very recently by Harris et al. for an AIL with a tetraalkylphosphopnium cation^30^. At a specific temperature, trihexyl(tetradecyl)phosphonium borohydride, noted as [P_666,14_][BH_4_], was found to undergo enhanced ordering of the alkyl chains in the nonpolar domains. Such a structural reorganization coincides well with the first-order thermodynamic transition, visible in calorimetric, XRD and IR spectroscopy data.

Motivated by this work, we embarked on a quest to identify not an isolated example but a systematically studied family of compounds that would exhibit LLTs, thereby gaining an insight into the structure-property relationships governing the LLT formation. Furthermore, by monitoring the relaxation dynamics of selected AILs under high-pressure conditions, we addressed the long-standing questions regarding the effect of compression on LLT and density fluctuations at T_LL_.

**Fig. 1:**
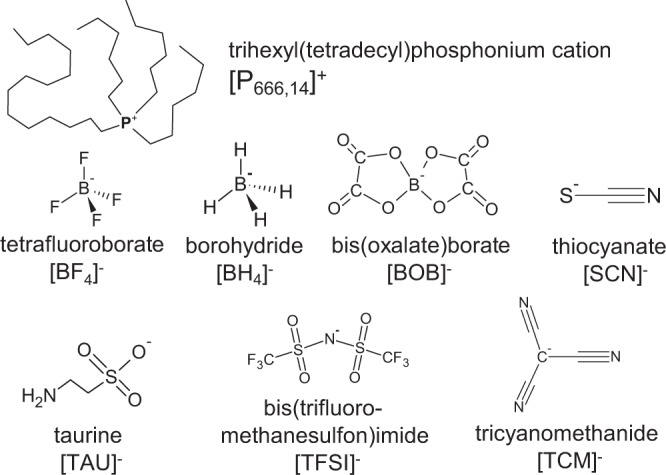
Chemical structures of studied ILs. Seven ionic liquids have been used in this study, containing the trihexyl(tetradecyl)phosphonium cation [P_666,14_]^+^ and anions: tetrafluoroborate [BF_4_]^−^, borohydride [BH_4_]^−^, bis(oxalate)borate [BOB]^−^, thiocyanate [SCN]^−^, taurine [TAU]^−^, bis(trifluoromethanesulfon)imide [TFSI]^−^, tricyanomethanide [TCM]^−^. The chemical structures are available in Supplementary Data 1.

We designed six AILs based on the [P_666,14_]^+^ cation, combined with six different anions (chemical structures shown in Fig. 1). The [P_666,14_][BH_4_] was also examined as a reference. The commonly used phosphonium cation imparted the AILs a relatively high thermal and electrochemical stability, as well as decent ionic conductivity and apolar/polar solvation ability^31^. Anions have been selected to reflect differences in size, geometry, conformational flexibility and coordinating ability (i.e., Lewis basicity). The linear thiocyanate, [SCN]^−^, trigonal planar tricyanomethanide, [TCM]^−^ and tetrahedral tetrafluoroborate [BF_4_]^−^ are all rigid due to their small size. The larger anions include a bulky and rigid bis(oxalate)borate [BOB]^−^, with distorted tetrahedral symmetry around the central boron atom, the bis(trifluoro-methanesulfon)imide, [TFSI]^−^ that is known to assume *cis* and *trans* conformations, and taurine, [TAU]^−^, featuring a flexible alkyl chain allowing for multiple conformations. The basicity of an anion that is—its ability to donate an electron pair can be quantified by Gutmann Donor Number (DN)^32^. On this scale, [TFSI]^−^ is a very weak donor, [TCM]^−^ is slightly stronger, whereas [SCN]^−^ is a moderately strong donor (Table 1). DN for [BOB]^−^ and [TAU]^−^ have not been measured, but [BOB]^−^ is reported to be a very weak donor, and [TAU]^−^ has an amine functionality, which by definition imparts its high propensity to share an electron pair (high DN).

**Table 1:** The thermodynamic characterization of studied systems.

	${T}_{{{{{{\rm{LLT}}}}}}}^{{{{{{\rm{c}}}}}}}$	−${\triangle H}_{{{{{{\rm{LLT}}}}}}}^{{{{{{\rm{c}}}}}}}$	${T}_{{{{{{\rm{LLT}}}}}}}^{{{{{{\rm{h}}}}}}}$	${\triangle H}_{{{{{{\rm{LLT}}}}}}}^{{{{{{\rm{h}}}}}}}$	*T*_g_	*T*_*LL*_ BDS	*T*_c_	−Δ*H*_c_	*T*_m_	Δ*H*_m_	*ν*	*DN*
Anion	K	J g^−1^	K	J g^−1^	K	K	K	J g^−1^	K	J g^−1^	nm^3^	kcal mol^−1^
[SCN]^−^	212.1* 211.6** 213.0*** 213.3	16.6* 14.9** 12.9*** 11.4	207.4* 206.2** 207.5*** 207.1	16.6* 15.1** 13.2*** 11.6	197.7^a^ 199.1^b^	210.3	231.4* 234.2** 251.9*** 254.3	38.0* 39.2** 9.51*** 1.9	270.8* 267.4** 273.0*** 272.3	58.2* 57.0** 18.1*** 5.0	0.0405 0.048^c^	45.9
[BH_4_]^−^	213.8* 214.6** 214.7*** 216.1	8.5* 6.4** 5.6*** 4.6	206.0* 206.8** 207.3*** 207.6	8.3* 6.7** 6.0*** 5.0	198.5^a^ 201.3^b^	210.9	222.5* 232.7** 242.3*** 250.9	51.1* 59.6** 62.8*** 34.5	270.7* 271.1** 270.4*** 271.9	82.5* 77.4** 73.8*** 34.6	0.03 0.046^c^	
[BF_4_]^−^	–	–	–	–	–	–	270.0* 229.0	49.3* 21.9	295* 295	61.4* 61.7	0.079 0.07^c^	7.3
[TCM]^−^	206.7* 206.8** 207.1*** 207.4	13.1* 12.3** 9.9*** 8.1	203.7* 204.4** 204.6*** 204.8	13.1* 12.0** 10.2*** 8.6	197.7^a^ 196.3^b^	203.6	–	–	–	–	0.121 0.093^c^	26.1
[TAU]^−^	214.9* 214.8** 215.8*** 216.1	6.0* 4.8** 4.4*** 3.8	201.6* 202.1** 203.3*** 203.9	6.1* 4.7** 4.1*** 3.9	197.5^a^ 199.5^b^	206.5	–	–	–	–	0.098^c^	
[BOB]^−^	213.4* 213.5** 214.5*** 216.1	0.64* 0.51** 0.42*** 0.38	202.2* 202.8** 203.7*** 204.8	1.3* 1.2** 1.1*** 0.8	202.1^a^ 198.4^b^	–	–	–	–	–	0.174 0.164^c^	
[TFSI]^−^	203.5* 204.3** 204.8*** 205.6	8.6* 7.3** 6.5*** 5.9	200.5* 200.4** 199.8*** 200.1	9.7* 8.2** 7.1*** 6.6	195.9^a^ 194.2^b^	201.2	–	–	–	–	0.161 0.163^c^	11.2

*T*_LL_ and Δ*H* denote respectively the temperature and enthalpy of liquid–liquid transition determined during the cooling (c) and heating (h) scan.

An increase of Δ*H*_LLT_ with a decreased scanning rate indicates that phase 1 needs a certain amount of time to transfer to phase 2.

*T*_*g*_ glass transition temperature, *T*_*c*_ crystallization temperature, *T*_*m*_ melting temperature, *ΔH*_*m*_ enthalpy of melting, *ΔH*_*c*_ enthalpy of crystallization, *v* molecular volume of anions.

^a^The glass transition temperature was determined from DSC measurements after 6 h aging at 183.15 K with the heating rate 10 K/min.

^b^The glass-transition temperature determined from BDS measurements according to definition T_g_ = T (σ_dc_ = 10^−14^ S/cm).

*1 K/min; **2 K/min; ***5 K/min; Others: 10 K/min.

^c^The van der Waals volume was calculated by using the Bondi method.

## Results and discussion

### Calorimetric studies of phase transitions

**Fig. 2:**
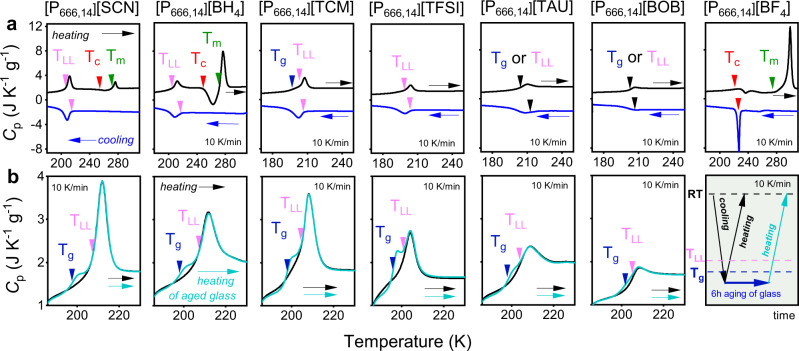
Differential scanning calorimetry (DSC) traces of [P_666,14_]^+^-based ILs. **a** The DSC data collected on cooling (blue curve) and subsequent heating (black curve) with the rate of 10 K min^−1^ of [P_666,14_][SCN], [P_666,14_][BH_4_], [P_666,14_][TCM], [P_666,14_][TFSI], [P_666,14_][TAU], [P_666,14_][BOB] and [P_666,14_][BF_4_] from left to right respectively. Arrows indicate the onset of: LLT (pink one), melting point (green one), cold crystallization (red one) and *T*_g_ (blue one). The values of liquid–liquid transition temperature (*T*_LL_), onset of cold crystallization (*T*_c_), melting temperature (*T*_m_) and enthalpy of these processes Δ*H* are collected in Table 1. **b** Comparison between DSC traces obtained during the standard heating with rate 10 K min^−1^ (black curved) and after the aging process performed in the glassy state, i.e., at 183 K (turquoise curve) of [P_666,14_][SCN], [P_666,14_][BH_4_], [P_666,14_][TCM], [P_666,14_][TFSI], [P_666,14_][TAU] and [P_666,14_][BOB] from left to right respectively. Pink arrows indicates the onset of LLT. The jump in the specific heat capacity during the liquid–glass transition is presented as blue arrows. On the last right panel the procedure of the DSC experiment is presented. Source data are provided as a Source Data file.

To firmly establish the LLT scenario, it is desirable to show its reversibility without crystallization. For this purpose, we firstly analyzed the conventional differential scanning calorimetry (DSC) thermograms obtained on cooling and subsequent heating of tested materials (Fig. 2a). Decreasing temperature with a standard rate of 10 K min^−1^ resulted in a step-like transition for [P_666,14_][BOB] and [P_666,14_][TAU], a sharp exothermic peak at 226 K for [P_666,14_][BF_4_], and a broader exotherm of much lower enthalpy observed at 205–216 K for the other four AILs. On heating, the thermal curve for [P_666,14_][BOB], [P_666,14_][TAU], [P_666,14_][TFSI], [P_666,14_][TCM], [P_666,14_][SCN] and [P_666,14_][BH_4_] featured an endotherm, quite symmetrical with respect to the cooling cycles. Note that this peak is the most pronounced for [TCM]^−^-based IL and the weakest for [BOB]^−^-IL. Upon further heating, [P_666,14_][SCN] and [P_666,14_][BH_4_] undergo cold crystallization and melting. An endotherm indicating the melting process of [P_666,14_][SCN] and [P_666,14_][BH_4_] was recorded at 272 K, while [P_666,14_][BF_4_] reveal *T*_m_ at 295 K. Herein, it should be stressed that the cold crystallization of [P_666,14_][BH_4_] was not reported in ref. ^30^. This indicates higher purity of material examined herein.

To examine the physical nature of phase transition observed in [P_666,14_]^+^-based AILs, DSC experiments with various scanning rates have been performed. As visible in Supplementary Note 1, a slower heating rate slightly moves the step-like increase of *C*_*p*_ towards lower temperatures for [P_666,14_][BOB] and [P_666,14_][TAU]. This, together with the very small enthalpy of this process, suggests its kinetic nature and thus simple vitrification (i.e., liquid–glass transition) of mentioned ILs. On the other side of the spectrum is [P_666,14_][BF_4_], where decreasing the scanning rate from 10 to 1 K min^−1^ shifted the exothermic peak from 229 to 270 K, whereas the endotherm occurred at around 295 K in both cases. Thus, [P_666,14_][BF_4_] crystallizes on cooling, and *T*_c_ increases with a decrease of cooling rate, which is typical behavior. Interestingly, a slight change of anion from [BF_4_] to [BH_4_] provides an IL with the supercooling ability and cold crystallization observed on a subsequent heating scan. In turn, independently of the applied heating rate, a broad endotherm observed for ILs with [BH_4_]^−^, [SCN]^−^, [TCM]^−^ and [TFSI]^−^ anions appears at the same temperature for given IL (*T* ± 1 K), with the only difference in Δ*H*, i.e., the lower the ramp rate, the higher the Δ*H* value (see Table 1). However, even the highest obtained Δ*H* is still around five times lower than the enthalpy of melting process Δ*H*_m_ obtained, e.g., for [P_666,14_][BF_4_]. Note that phase 2 observed below the endotherm is an optically transparent homogenous disordered phase, as confirmed by microscopic observations. These findings lead to conclusion that the prominent peak observed in DSC thermograms of [P_666,14_][TFSI]_,_ [P_666,14_][TCM], and at lower temperature range for [P_666,14_][SCN], and [P_666,14_][BH_4_] indicates the liquid–liquid phase transition (LLT). Herein, it should be mentioned that in addition to LLT, the liquid–glass transition of [P_666,14_][TCM] is also detectable at *T* < *T*_LL_. (see Fig. 2a and Supplementary Fig. 30). However, *T*_g_ does not appear on thermogram of any other examined IL. Since an amorphous phase has a non-equilibrium nature, its thermodynamic and dynamic properties (e.g., the specific volume, enthalpy or relaxation dynamics) evolve over time^33^. This phenomenon, known as physical aging, is accompanied by an increase of heat capacity in the glass transition region (so-called overshoot peak) and therefore can be useful to reveal *T*_g_ of [P_666,14_]^+^-based ILs. Therefore, in the further step, aging experiments have been performed on all [P_666,14_]^+^ ILs, excluding that of [BF_4_]^−^ anion, to confirm that cooling transforms liquid 2 to the glassy state. The experimental protocol of aging involves: (i) decreasing temperature to 183 K (the *T* expected to fall below *T*_g_), (ii) time-dependent isothermal step at this *T*, and (iii) subsequent heating. The thermograms corresponding to the final heating scans are compared with data obtained in standard DSC measurement in Fig. 2b. As a result of the aging experiments, the liquid–glass transition emerged at *T* < *T*_LL_ for 4 samples, i.e., ILs with [BH_4_]^−^, [SCN]^−^, [TCM]^−^ and [TFSI]^−^ anions. However, at the same time, for [P_666,14_][TAU] and [P_666,14_][BOB] there are no overshoot peaks that would confirm typical liquid–glass transition in these ILs (see Supplementary Fig. 31 for comparison). Instead, a slight increase of *C*_*p*_ is visible on the low-temperature side of the endotherm, while the *C*_*p*_^max^ remains unchanged. This indicates that both *T*_g_ and *T*_LL_ co-exist in the close vicinity for [P_666,14_][TAU] and [P_666,14_][BOB]. The values of *T*_g_ determined from the heating scan of aged glass with the rate of 10 K min^−1^ and the onset points of LLT are collected in Table 1.

A closer inspection of the DSC results indicates that the temperature of liquid–glass transition remains constant for studied ILs while T_LL_ decreases in the following order: [BH_4_]^−^ < [SCN]^−^ < [TCM]^−^ < [TAU]^−^ < [TFSI]^−^ < [BOB]^−^ that corresponds well with decreasing basicity and increasing van der Waals volume of the anion. Noteworthy, the sign of LLT is the weakest for IL with large, rigid and non-coordinating anion [BOB]^−^. Interestingly, more flexible, lower symmetry [TFSI]^−^ of similar size induced the well-detectable LLT. Furthermore, the small, symmetrical and non-coordinating [BF_4_]^−^ induced crystallization, whereas for [BH_4_]^−^ both liquid–glass and LLTs are also observed. On the other hand, [SCN]^−^ which is small but has lower symmetry and higher basicity than [BF_4_]^−^, also featured LLT and crystallization/melting behavior. Consequently, it appears that LLT is relatively easy to induce in [P_666,14_]^+^-based ILs, but certain conditions concerning size, symmetry, conformational flexibility and possibly Lewis basicity of the anions must be met. One can deduce that the *V*^vdW^ < 0.1 nm^3^ for rigid structure and *V*^vdW^ < 0.2 nm^3^ for flexible one is the limiting value to observe clear LLT in [P_666,14_]^+^-based ILs. Too large anions most likely complicate the ordering of the alkyl chains in the nonpolar domains and consequently prevent the LLT.

### Changes in ion dynamics accompanying LLT

To further advance the knowledge on LLT in [P_666,14_]-based ILs, dielectric spectroscopy has been employed. First, the single-frequency experiments, analogous to DSC scans, have been performed for all AILs. The evolution of dielectric constant (*ε*′) at 1 MHz, accompanying cooling and subsequent heating of [P_666,14_][TFSI]_,_ [P_666,14_][TCM], [P_666,14_][BOB], [P_666,14_][TAU], [P_666,14_][SCN] and [P_666,14_][BF_4_] at 1 K min^−1^, is illustrated in Supplementary Fig. 32. The *ε*′(*T*) curves obtained for the [TAU]^−^, [TFSI]^−^ and [TCM]^−^ samples reveal reversible character with a step-like signature of LLT. In turn, the behavior of ε′ for [P_666,14_][BOB] and [P_666,14_][BF_4_] resembles the characteristics of the liquid–glass transition and crystallization, respectively, which corresponds to calorimetric data. At the same time, [P_666,14_][SCN] shows LLT, crystallization, and melting processes during the heating scan, which agrees with DSC results.

**Fig. 3:**
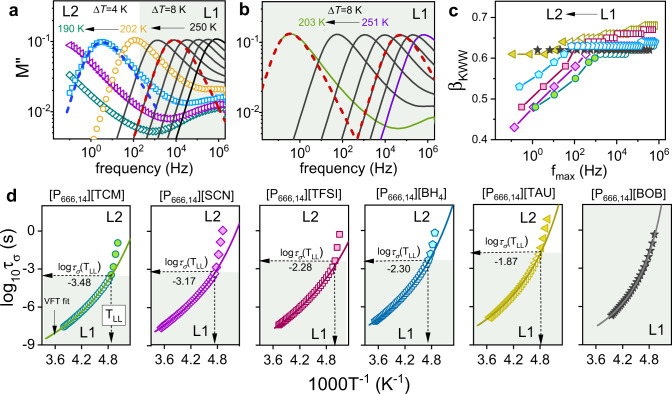
Dielectric response of studied ILs measured at ambient pressure conditions. **a** The representative dielectric data of [P_666,14_][TFSI] in liquid 1 (solid lines) and liquid 2 (scatters) phases recorded on cooling and presented in electric modulus representation. Dashed lines denote fits of KWW function to experimental data with *β*_KWW_ = 0.65 (red dashed line) and 0.53 (blue dashed line), in liquid 1 and liquid 2, respectively. **b** The representative dielectric data of [P_666,14_][BOB] measured in the supercooled liquid 1 state. Dashed lines denote fits of the KWW function with the exponent equal to 0.62. Note that the same value of *β*_KWW_ was used in the close vicinity of *T*_g_ and far from liquid–glass transition. **c** The *β*_KWW_ exponent is plotted as a function of the frequency of modulus peak maximum for all studied ILs. **d** Temperature dependence of conductivity relaxation time for [P_666,14_][TCM], [P_666,14_][SCN], [P_666,14_][TFSI], [P_666,14_][BH_4_], [P_666,14_][TAU], and [P_666,14_][BOB] (from left to right respectively). Scatters-experimental data, solid lines denote the fit of VFT function ${\tau }_{\sigma }={\tau }_{\infty }{{\exp }}\big(\frac{D{T}_{0}}{T-{T}_{0}}\big)$ to experimental data. Dashed lines indicate *T*_LL_ and *τ*_*σ*_ at LLT. The type and color or data points in **c**, **d** are specific for a given IL. The color area on **a**, **b** and **d** denotes the liquid 1 phase. L1 denotes liquid 1 while L2 liquid 2. Source data are provided as a Source Data file.

To verify whether the LLT manifests itself in molecular dynamics behavior, the dielectric measurements over a wide frequency (10^−2^–10^7^ Hz) and temperature range were performed. For ionic systems, the translational displacement of charge carriers (dc-conductivity) dominates the dielectric loss *ε*″(f) function, conventionally employed for data analysis^34^. Therefore, complex electric conductivity σ*(*f*) = *ε*_0_(Z*(*f*)*C*_0_)^−1^ and complex electric modulus M*(*f*) = ε*(*f*)^−1^, are usually adopted to express their dielectric properties^35^. The latter formalism allows for the determination of three relevant quantities describing the ion dynamics in AILs: dc-conductivity *σ*_dc_ = 2π*fε*_0_(*M*″)^−1^ calculated from a low-frequency region of *M*″(*f*); conductivity relaxation times *τ*_*σ*_ = (2π*f*_max_)^−1^ determined directly form *M*″ maximum; and distribution of relaxation times reflected in the width of *M*″(*f*) peak. Therefore, this representation was selected to evaluate the data recorded in this work. [P_666,14_][BF_4_] has been excluded from these studies due to the high tendency to crystallization. Figure 3a shows the representative electric modulus spectra of [P_666,14_][TFSI] collected at 0.1 MPa and various temperatures. This graph shows that the *M*″(*f*) peak (denoted as *σ*-process or conductivity relaxation peak), describing the time scale of translational motions of ions, shifts to lower frequencies upon cooling. This gradual change is in keeping with cooling effects seen in other ionic systems and reflects ions’ suppressed mobility^36^. However, starting from a certain temperature, coinciding well with the calorimetric LLT, the temperature sensitivity of the *σ*-process becomes markedly stronger while the *M*″ peak is broadening significantly. These effects, visualized for [P_666,14_][TFSI], are characteristic of all studied herein AILs (see Supplementary Note 2 for all *M*″ spectra collected for studied herein IL) except for [P_666,14_][BOB]. For the latter ILs, the temperature decrease brings a usual slowing down of ion dynamics, i.e. the *M*″(*f*) peak is keeping the same shape over a broad *T* range (see Fig. 3b).

To quantify changes in the shape of conductivity relaxation peak across the LLT, the Kohlrausch function, $\phi (t)={{\exp }}[-{(t/{\tau }_{{{\upalpha }}})}^{{{{\upbeta }}}_{{{{{{\rm{KWW}}}}}}}}]$^37^, has been used (exemplary fitting curves are presented in Fig. 3a, b). The *β*_KWW_ parameters obtained from fitting of *M*″ peaks are plotted as a function of the frequency of *M*″ peak maximum (*f*_max_) in Fig. 3c. It is well-known that the broader and more asymmetric the peak is, the lower is the value of *β*_KWW_. As shown in Fig. 3c, for a given IL, the exponent characterizing the liquid 1 stays approximately constant and falls in the range 0.62 < *β*_KWW_ < 0.67, which is in the middle of the range reported for various ionic glass-formers^38^. On the other hand, a transition to liquid 2 brings a substantial decrease of *β*_KWW_. The most significant drop is denoted for AIL with [SCN]^−^ anion. On the second place [TCM]^−^ is located, then [TFSI]^−^, [BH_4_]^−^ and the weakest drop occurs for IL with the [TAU]^−^. Interestingly, this order corresponds well with the decrease of enthalpy accompanying LLT. The obtained results indicate that the distribution of the relaxation times becomes broader during the transformation from liquid 1 to liquid 2. In other words, in liquid 1 the species are more dynamically correlated (i.e., components relax with similar *τ*_*σ*_), whereas in phase 2 there is higher heterogeneity (i.e., some components are more mobile and some are less mobile). This result is in agreement with dielectric data measured across LLT for TPP^39^.

At the same time, the LLT of [P_666,14_][BOB] is not detectable on *β*_KWW_(*f*_max_) graph mainly because the transition to liquid 2 overlaps with the liquid–glass transition, where the conductivity relaxation is too long to be directly measured (*f*_max_(*T*_g_) = 1.6·10^−3^ Hz) Consequently, the dielectric response of liquid 1 is recorded over the entire available frequency range, i.e., from 10^6^ to 10^−2^ Hz. In this context, it is not surprising that the shape of *M*″(*f*) function collected for [P_666,14_][BOB] is invariant, i.e., satisfy the time-temperature superposition (TTS) rule being a typical behavior of glass-forming systems in a supercooled state.

The temperature dependence of conductivity relaxation times *τ*_*σ*_(*T*^−1^), calculated directly from the modulus peak maxima, also exhibits a peculiar behavior near the calorimetric LLT (Fig. 3d). In particular, *τ*_*σ*_ collected in liquid 1 reveals a typical non-Arrhenius behavior, and a substantial departure from the Vogel–Fulcher–Tammann (VFT) law occurs at the onset of phase transition; that is, an abrupt increase is observed in apparent activation energy. A closer inspection of Fig. 3d reveals that the values of *τ*_*σ*_(*T*_LL_) are in the range 0.3–15 ms for most of the studied AILs that is far from the time scale commonly identified with the liquid–glass transition (*τ*_*σ*_ = 100 s). The LLT is also clearly detectable when the Stickel operator, [dlog*τ*_*σ*_/d1000*T*^−1^]^−0.5^, is applied; such procedure gives two linear regions that intersect at *T*_LL_ (Supplementary Fig. 34). Notably, the sign of LLT is also detectable in dc-conductivity (*σ*_dc_) behavior. As shown in Supplementary Fig. 35, further cooling of ILs below LLT brings a change of *σ*_dc_(*T*^−1^) from VFT to Arrhenius behavior observed at *σ*_dc_ = 10^−14^ S cm^−1^, which is commonly accepted as the value characterizing *T*_g_. Note that *T*_g_ values determined as a crossover point of *σ*_dc_(*T*^−1^) dependencies are in good agreement with calorimetric *T*_g_ of aged glass (see Table 1).

As could be expected from raw dielectric spectra, the log*τ*_*σ*_(*T*^−1^) and *σ*_dc_(*T*^−1^) data obtained for [P_666,14_][BOB] do not reveal any peculiar behavior. Namely, the experimental points follow a single VFT equation in almost the whole examined temperature range. The only deviation from VFT to Arrhenius dependence occurs at *σ*_dc_ = 10^−14^ S/cm and indicates the liquid–glass transition (see Fig. 3d and Supplementary Fig. 35).

### The LLT under high-pressure conditions

Although rapid cooling is probably the most straightforward method for inducing a first-order phase transition, it is not the only route. The LLT of phosphorus^40^ or nitrogen^41^ can also be realized by isothermal compression. However, according to experimental results reported in the literature, to induce LLT in these systems, extreme temperature and pressure conditions have to be applied, for example, 50 GPa and 1920 K for nitrogen. In this context, one may question the possibility of achieving LLT in [P_666,14_]^+^ ILs through the isothermal compression.

**Fig. 4:**
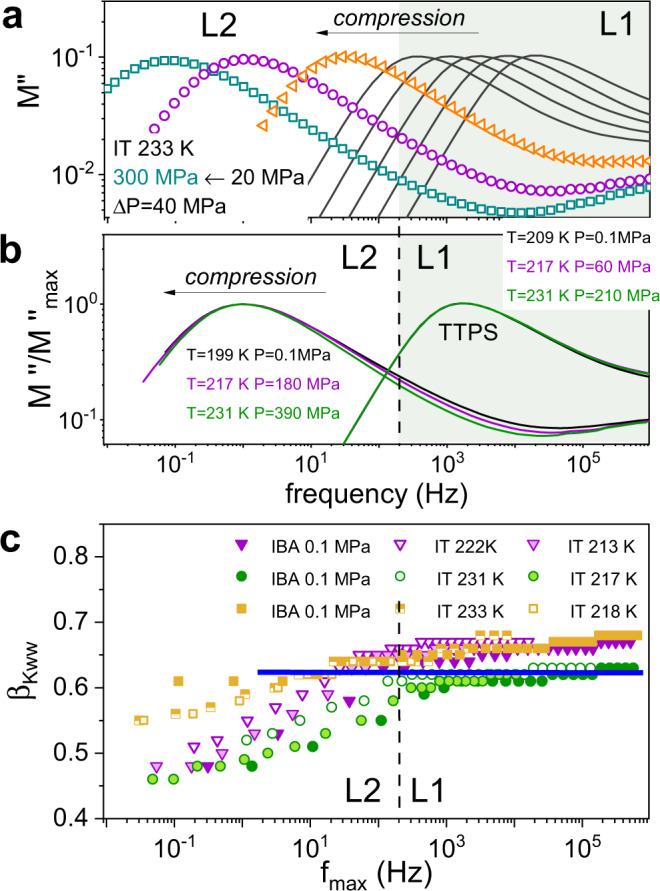
Dielectric response of studied ILs measured at high-pressure conditions. **a** The representative dielectric data recorded for [P_666,14_][TCM] in liquid 1 (solid lines) and liquid 2 (scatters) phases, recorded during compression at isothermal (IT) conditions of 233 K and presented in electric modulus representation. **b** The representative *M*″(*f*) spectra of [P_666,14_][TCM] recorded at various *T-P* conditions however at the same τ_σ_ superimposed to each other in liquid 1 and liquid 2, respectively. Note that time temperature pressure superposition (TTPS) rule is valid. **c** The *β*_KWW_ exponent is plotted as a function of the frequency of modulus peak maximum at various thermodynamic conditions. IBA denotes 0.1 MPa and various temperatures, while IT denotes isotherm and various pressure. Triangles-[P_666,14_][TFSI], circles-[P_666,14_][TCM], squares-[P_666,14_][TAU]. The horizontal line indicates *β*_KWW_ for [P_666,14_][BOB] being constant at various *T-P* conditions. The color area on **a**, **b** denotes the liquid 1 phase. L1 denotes liquid 1 while L2 liquid 2. Source data are provided as a Source Data file.

Four ILs have been chosen for high-pressure tests: [P_666,14_][TCM] and [P_666,14_][TFSI]—as revealing the most spectacular changes in relaxation dynamics across the LLT at 0.1 MPa, as well as [P_666,14_][BOB] and [P_666,14_][TAU]—as counterexamples. The representative high-pressure spectra collected at 223 K for [P_666,14_][TCM] are shown in Fig. 4a (see Supplementary Note 2 for all collected high-pressure data). The observed pattern of behavior upon compression is analogous to the isobaric cooling experiment: despite maintaining the same pressure step, after reaching certain pressure the shifts in *f*_max_ are markedly faster. Also, the shape of the *M*″(f) peak behaves similarly to the ambient pressure experiment—during the isothermal compression, past certain pressure, the peaks broaden significantly. A direct comparison of the spectra collected at given *f*_max_ under various *T-P* conditions shows that the shape of the *σ*-relaxation is independent of thermodynamic variables if phase 1 is considered (Fig. 4b and Supplementary Fig. 38). Such a phenomenon, called the temperature-pressure superposition principle, is a typical feature of glass-forming materials^42^. However, for superimposed spectra of liquid 2, the shape of *M*″(*f*) function at a given value of *τ*_*σ*_ becomes narrower with increasing *T* and *P*. In other words, compression at higher temperatures reduces the distribution of relaxation times in phase 2, making it more homogenous in terms of molecular dynamics. The same pattern has been detected for [P_666,14_][TFSI]; however, the dielectric spectra of [P_666,14_][TAU] are getting slightly broader under pressure. In contrast, the *σ*-dispersion in [P_666,14_][BOB] has been constant at any chosen *τ*_*σ*_ (see Fig. 4c and Supplementary Fig. 38). Consequently, the changes of *M*″(*f*) peak with pressure cannot be treated as a feature unique to [P_666,14_]^+^ ILs, but rather as a unique characteristic of the liquid–liquid transformation. This peculiar behavior is, to some extent, similar to the effect of pressure on polymerization reactions when the material of a narrower distribution of molecular weight is obtained under higher pressure^43^.

**Fig. 5:**
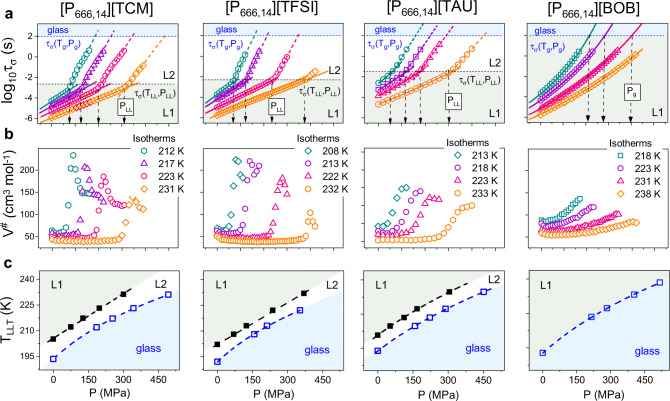
High-pressure data of [P_666,14_][TCM], [P_666,14_][TFSI], [P_666,14_][TAU], and [P_666,14_][BOB] (from left to right respectively). **a** Presents the pressure dependence of conductivity relaxation time measured at various *T*. Solid lines denote the *p*VFT fit ${\tau }_{\sigma }={\tau }_{0}{{\exp }}(\frac{{BP}}{{P}_{0}-P})$ to experimental data. Dashed lines indicate liquid–liquid transition pressure (*P*_LL_) and *τ*_*σ*_ at LLT. *P*_g_ denotes liquid–glass transition pressure. **b** Presents pressure dependence of apparent activation volume, *V*^#^. The four-time increase in *V*^#^ at LLT indicates a formation of nonpolar domains of large scale in liquid 2. **c** *T*_LL_ and *T*_g_ as a function of *P* is presented. The color area on **a** and **c** denotes the liquid 1 phase (gray) and glass region (blue). L1 denotes liquid 1 while L2 liquid 2. Source data are provided as a Source Data file.

The analysis of isothermal *τ*_*σ*_*-P* dependences determined for the studied ILs reveals another intriguing feature of the LLT. As illustrated in Fig. 5a, isothermal compression has fundamentally the same effect on the ion dynamics as isobaric cooling, i.e. the conductivity relaxation times *τ*_*σ*_ are getting longer with squeezing. The *τ*_*σ*_(*P*) data of [P_666,14_][BOB] follow the *p*VFT behavior over the entire examined pressure range, while the experimental points recorded for [P_666,14_][TCM], [P_666,14_][TAU] and [P_666,14_][TFSI] markedly rise in slope at certain *P* bringing an almost four-fold increase in apparent activation volume *V*^#^ = 2.303*RT*(dlog*τ*_*σ*_/d*P*)_*T*_. (Fig. 5b). However, what is interesting, the kink of the *τ*_*σ*_*-P* curve, being a manifestation of LLT, is independent of *T-P* conditions and appears at constant conductivity relaxation times for a given system; specifically, at *τ*_*σ*_ of milliseconds. This result shows that in analogy to liquid–glass transition, the L-L transformation is isochronal in nature. Nevertheless, the time scale of ion dynamics at LLT, *τ*_*σ*_(*T*_LL_,*P*_LL_) is not universal but depends on intermolecular interactions.

Defining *P*_LL_ as the pressure at which the activation volume starts to increase, we obtain the pressure dependence of *T*_LL_ plotted in Fig. 5c. As presented, *T*_LL_ increases with pressure in a linear fashion, with the slope equal to 81 K GPa^−1^ for [P_666,14_][TFSI], 87 K GPa^−1^ for [P_666,14_][TCM] and 82.4 K GPa^−1^ for [P_666,14_][TAU]. It means that the pressure in the order of 1.2 GPa is required to observe the LLT at room temperature conditions. Interestingly, when the isothermal *τ*_*σ*_(*P*) data are extrapolated to *τ*_*σ*_ = 100 s (that is a standard definition of *T*_g_, i.e. *T*_g_ = *T*(*τ*_*σ*_ = 100 s) or *σ*_dc_ = 10^−14^ S cm^−1^, non-linear *T*_g_(*P*) dependences with the d*T*_g_/d*P* coefficient of 125 ± 5 K GPa^−1^ is obtained for [P_666,14_][TFSI], [P_666,14_][TCM] and [P_666,14_][BOB]. This indicates that dynamics of phase 2 of [P_666,14_][TCM] and [P_666,14_][TFSI] freezes (transform to glass) with the same characteristic as supercooled liquid 1 of [P_666,14_][BOB]. The d*T*_g_/d*P* for [P_666,14_][TAU] was slightly lower (106 K GPa^−1^), but still much higher than d*T*_LL_/d*P*.

The determined values of d*T*_LL_/d*P* offer a unique possibility to estimate the variations in volume accompanying the LLT (Δ*V*_LLT_) using a simple Clausius-Clapeyron equation, d*P*/d*T* = Δ*S*_LLT_(Δ*V*_LLT_)^−1^. The Δ*S*_LLT_ denotes the entropy changes during the LLT and can be obtained as Δ*S*_LLT_ = Δ*H*_LLT_(*T*_LLT_)^−1^, where Δ*H*_LLT_ is the enthalpy of first-order transition. The calculated values of Δ*V*_LLT_ are equal to 0.0056 cm^3^ g^−1^, 0.0039 cm^3^ g^−1^ and 0.0025 cm^3^ g^−1^ for [P_666,14_][TCM], [P_666,14_][TFSI] and [P_666,14_][TAU] respectively. To put these numbers into perspective, we measured the density of these ILs as a function of temperature and extrapolated the obtained dependence to *T*_LLT_ (see Supplementary Fig. 39). Such procedure gives *V*_LLT_ = 0.8759 cm^3^ g^−1^ for [P_666,14_][TFSI], 0.9944 cm^3^ g^−1^ for [P_666,14_][TAU] and 1.0452 cm^3^ g^−1^ for [P_666,14_][TCM], indicating that changes of around 0.5% occur at LLT, that is within the error in dilatometric measurements. These small variations in the specific volume can be easily overlooked in direct *V*(*T,P*) measurements and reveal that density is not the dominant order parameter governing the LLT in ILs. This is in agreement with the experimental data recorded for sodium acetate trihydrate (CH_3_COONa·3H_2_O) where a first-order LLT without density discontinuity was identified^19^. Moreover, LLTs without density change were suggested to take place in several metallic glass-formers^44^.

In summary, our findings provide experimental support for the hypothesis that the LLT occurs in ion-containing systems. Our studies of a series of [P_666,14_]^+^-based AILs and seven different anions demonstrate that the LLT takes place in AILs with [TCM]^−^, [TFSI]^−^, [TAU]^−^, [BH_4_]^−^ and [SCN]^−^ anions, but not with [BF_4_]^−^ that shows crystallization. On the other hand, for [P_666,14_][BOB], the LLT overlaps with the liquid–glass transition. Interestingly, *T*_g_ is approximately the same for all studied AILs, while *T*_LL_ is decreasing with an increase of anion van der Waals volume. This demonstrates that ILs can be fine-tuned to display *T*_LL_, which is dependent on several parameters, such as anion size, geometry, conformational flexibility, Lewis basicity and the strength of interionic interactions. In this context, the question about the differences between liquid structures formed by [P_666,14_]^+^-based AILs appears and should be addressed in the future e.g. by using SAXS or neutron scattering.

Our study also provides an important approach to the LLT-correlated properties. We found that the parameters characterizing the ion dynamics (*τ*_*σ*_*, σ*_dc_) and distribution of relaxation times (*β*_KWW_) monitored on isobaric cooling and isothermal compression reveal peculiar behavior at the LLT. Furthermore, independently of *T-P* conditions, the sign of LLT is observed at *τ*_σ_ = const. within a given system, i.e., it occurs at a certain time scale of ionic motions dependent on interionic interactions. Further studies of this issue e.g. by using dynamic light scattering (DLS) under high pressure conditions are desired. Upon transition liquid 1→liquid 2 (induced by cooling or high-pressure), the AILs become more heterogeneous in terms of ion mobility. Additionally, the d*T*_LL_/d*P* coefficient determined for [TCM]^−^, [TAU]^−^ and [TFSI]^−^-based ILs in a high-pressure experiment, together with Δ*H*_LL_ and *T*_LL_ let us estimate the volume changes accompanying LLT by using the Clausius-Clapeyron equation. We found that Δ*V*_LL_ is very small and can be undetectable in conventional dilatometry measurement. Such an LLT may offer a unique opportunity for investigating the subtle structural and dynamic changes of liquid.

## Methods

### Synthesis

#### [P_666,14_][TFSI]

Trihexyl(tetradecyl)phosphonium chloride [P_666,14_][Cl] (0.010 mol eq.) and Li[TFSI] were separately dissolved in deionised water, combined in a round-bottomed flask, and left to react (1 h, RT, 600 rpm). The organic layer was separated and washed with deionized water and then dichloromethane. Subsequent washes were performed with solution of Li[TFSI] in deionised water, and then with deionised water, until no chloride could be detected with silver nitrate solution. DCM was removed via rotary evaporation (30 min, 35 °C) and the ionic liquid was dried under high vacuum (12 h, 70 °C, 10^−2^ mbar). The product was analysed by ^1^H, ^13^C, ^19^F and ^31^P NMR spectroscopy (d_6_-DMSO) and by XRF for chloride content. [P_666,14_][SCN], [P_666,14_][TCM] and [P_666,14_][BF4] were prepared following analogous procedures, from their respective salts.

#### [P666,14][TAU]

[P_666,14_][Cl] was converted to [P_666,14_][OH] (solution in methanol). Taurine (0.012 mol eq.) was dissolved in deionised water, to which methanol solution of [P_666,14_][OH] (0.015 mol eq.) was added and left to react (2 h. RT, 600 rpm). Solvents were then removed via rotary evaporation (3 h, 30–80 °C), and the crude product was dissolced in dry acetonitrile and stored in the fridge (5 °C, 12 h) until the excess taurine crystallised and was filtered off. Acetonitrile was removed via rotary evaporation (30 min, 60 °C) and the ionic liquid was dried under high vacuum (overnight, 70 °C, 10^–2^ mbar). The product was analysed 1H, 13C and 31P NMR spectroscopy (d6-DMSO) and by XRF for chloride content.

#### [P666,14][BH4]

[P_666,14_][Cl] (0.10 mol eq.) and Na[BH_4_] (0.13 mol eq.) were separately dissolved in deionised water, combined in a round-bottomed flask, and left to react (48 h, RT, 600 rpm). Chloroform was added to dissolve the ionic liquid layer, and the aqueous layer was separated. The organic layer was washed ten times with Na[BH_4_] solution, then chloroform was removed via rotary evaporation (30 min, 35 °C) and the solution was dried overnight under high vacuum (12 h, 70 °C, 10^−2^ mbar). Chloride removal was carried out in three subsequent polishing steps (multiple sodium borohydride washes) until chloride content was below 2000 ppm by XRF analysis.

#### [P_666,14_][BOB]

Was synthesized in a two-step synthesis. Firstly, oxalic acid (0.03 mol eq.) and boric acid (0.01 mol eq.) were separately dissolved in water and then combined under vigorous stirring. Na_2_CO_3_ (0.5 mol eq.) was slowly added and the turbid solution was heated in an oil bath at 120 °C. Water was distilled off until a dry white powder was obtained, which then was dispersed in hot acetonitrile (60 °C, 1 h), filtered, washed with cold ethanol and finally dried overnight under high vacuum (12 h, 60 °C, 10^−2^ mbar). Subsequently, [P_666,14_][Cl] (0.01 mol eq.) and Na[BOB] (0.01 mol eq.) were stirred in dichloromethane (overnight, RT, 600 rpm) and then water was added, inducing phase separation. The organic layer was separated and washed with solution of Na[BOB] in deionized water, then with deionised water, until no chloride could be detected with silver nitrate solution. Subsequently, DCM was removed via rotary evaporation (30 min, 35 °C) and the ionic liquid was dried overnight under high vacuum (12 h, 60 °C, 10^−2^ mbar). For more details see Supplementary Information file.

#### Differential scanning calorimetry (DSC)

Calorimetric experiments of studied ILs were performed by means of a Mettler Toledo DSC1STAR System equipped with a liquid nitrogen cooling accessory and an HSS8 ceramic sensor (a heat flux sensor with 120 thermocouples). Each sample with a mass of around 10–20 mg was measured in aluminum crucibles with a 40 μL volume. During the experiments, the flow of nitrogen was kept at 60 mL min^–1^. Enthalpy and temperature calibrations were performed using indium and zinc standards. Low-temperature verification was made using CCl_4_ and *n*-heptane (182.15 K, 140.5 J g^−1^) at different scanning rates (0.7, 1, 5, and 10 K min^−1^). The baseline was constructed as a straight line from the onset to the endpoint. A dedicated software Mettler Toledo DSC1STAR allows various calculations (onset, heat, peak temperature, etc.) from the original recorded DSC curves. Prior to the measurement, the samples were annealed 30 min at 373 K. Temperature ramps involved cooling to 143 K and then heating to 373 K with a rate of 10 K per min. Samples were cycled at least 3 times to ensure reproducibility and high accuracy. The 6-h aging experiment was performed at 183 K after cooling with the rate of 10 K min^−1^.

#### Dielectric measurements

The dielectric measurements at ambient pressure for studied ILs were carried out over a frequency range from 10^−1^ Hz to 10^7^ Hz by means of Novo-Control GMBH Alpha dielectric spectrometer. The Novocool system controlled the temperature with an accuracy of 0.1 K. During this measurement the sample was placed between two stainless steel electrodes (diameter = 15 mm). The distance of 0.08 mm was provided by the quartz ring. For the pressure-dependent dielectric measurements, we used the capacitor filled with the studied sample, which was next placed in the high-pressure chamber and compressed using silicone oil. Note that during the measurement, the sample was only in contact with stainless steel. The pressure was measured by the Unipress setup with a resolution of 1 MPa. The temperature was controlled within 0.1 K by means of a Weiss fridge.

## Supplementary information

Supplementary Information

Description of Additional Supplementary Files

Supplementary Data 1

## Data Availability

All data generated or analyzed during this study are included in this published article (and its Supplementary information files). Source data are provided with this paper.

## Source data

Source Data
