# Supplementary material for: Pressure-induced liquid-liquid transition in a family of ionic materials
Source: Nat Commun. 2022 Mar 15;13:1342. doi: 10.1038/s41467-022-29021-0 (PMC8924164; doi:10.1038/s41467-022-29021-0)
Supplement: Supplementary file 1 — Supplementary Information [file 41467_2022_29021_MOESM1_ESM.pdf]

Supplementary Information:
Pressure-induced liquid-liquid transition in a family of ionic materials

Z. Wojnarowska et al.

Supplementary Methods

Trihexyl(tetradecyl)phosphonium chloride, $[P_{66614}]Cl$, was kindly provided by Solvay. Sodium tricyanomethanide was bought from TCI Chemicals. All other chemicals were purchased from Sigma-Aldrich and used as received. XRF analysis was performed on a Rigaku NEX QC+ QuantEZ High-Resolution Energy Dispersive X-ray Fluorescence (EDXRF) Spectrometer. NMR spectra were recorded on either a Bruker Avance III 400 MHz spectrometer or a Bruker Avance II DPX 600 MHz spectrometer.

$[P_{666,14}]$[TFSI]. Trihexyl(tetradecyl)phosphonium chloride, $[P_{666,14}]Cl$ (0.010 mol eq.) and lithium bis(trifluoromethanesulfon)imide Li[TFSI] (0.013 mol eq.) were separately dissolved in 25 cm^3 deionised water (18.2 MΩ.cm) (total 50 cm^3) and then combined in a round-bottomed flask (250 cm^3), resulting in the formation of a biphasic liquid system; the mixture was left to react (1 h, room temperature, 600 rpm). The aqueous layer was separated, and the organic layer was collected and washed, firstly with deionized water (18.2 MΩ.cm) (10 cm^3) and then dichloromethane, DCM (10 cm^3). Subsequent washes were performed with solution of Li[TFSI] in deionised water (18.2 MΩ.cm). Final three washes were performed with deionised water (18.2 MΩ.cm) until no chloride could be detected with silver nitrate solution. Subsequently, DCM was removed *via* rotary evaporation (30 min, 35 °C) and the ionic liquid was dried under high vacuum (12h, 70 °C, 10^{-2} mbar). XRF analysis confirmed chloride content was below the detectable limit. ^{1}H, ^{13}C, ^{19}F and ^{31}P NMR spectra of the IL were recorded in d_6-DMSO.

^{1}H NMR (400.13 MHz, d_6 -DMSO) δ: 0.83-0.92 (m, 12H), 1.21-1.27 (m, 18H), 1.28-1.35 (m, 14H), 1.35-1.43 (m, 8H), 1.43-1.55 (m, 8H), 2.11-2.23 (m, 8H).

$^{13}C\{^{1}H\}$ NMR (100.61 MHz, d_6 -DMSO) δ: 13.53 (s, P-$(CH_2)_5$-***CH_3***)., 13.64 (s, P-$(CH_2)_{13}$-***CH_3***)., 17.45 (d, $^{1}J_{C/P}$ = 188 Hz, P-***CH_2***-$(CH_2)_4$-CH_3), 17.52 (d, $^{1}J_{C/P}$ = 192 Hz, P-***CH_2***-$(CH_2)_{12}$-CH_3), 20.46 (s,), 20.50 (s,), 20.54 (s,), 21.75 (s,), 22.07 (s,), 28.81 (d, $^{2}J_{C/P}$ = 116 Hz, P-CH_2-***CH_2***-$(CH_2)_3$-CH_3), 28.90 (d, $^{2}J_{C/P}$ = 128 Hz, P-CH_2-***CH_2***-$(CH_2)_{11}$-CH_3), 29.04 (s, P-$(CH_2)_4$-***CH_2***-CH_3), 29.09 (s,), 29.70 (d, $^{3}J_{C/P}$ = 60 Hz, P-$(CH_2)_2$-***CH_2***-$(CH_2)_{10}$-CH_3), 29.95 (d, $^{3}J_{C/P}$ = 60 Hz, P-$(CH_2)_2$-***CH_2***-$(CH_2)_2$-CH_3), 30.34 (s, P-$(CH_2)_3$-***CH_2***-$(CH_2)_9$-CH_3), 31.32 (s, P-$(CH_2)_3$-***CH_2***-CH_2-CH_3), 119.50 (q, $^{1}J_{C/F}$ = 1280 Hz CF_3).

^{19}F NMR (376.50 MHz, d_6 -DMSO) δ: -79.00.

$^{31}P\{^{1}H\}$ NMR (161.98 MHz, d_6 -DMSO) δ: 33.55.

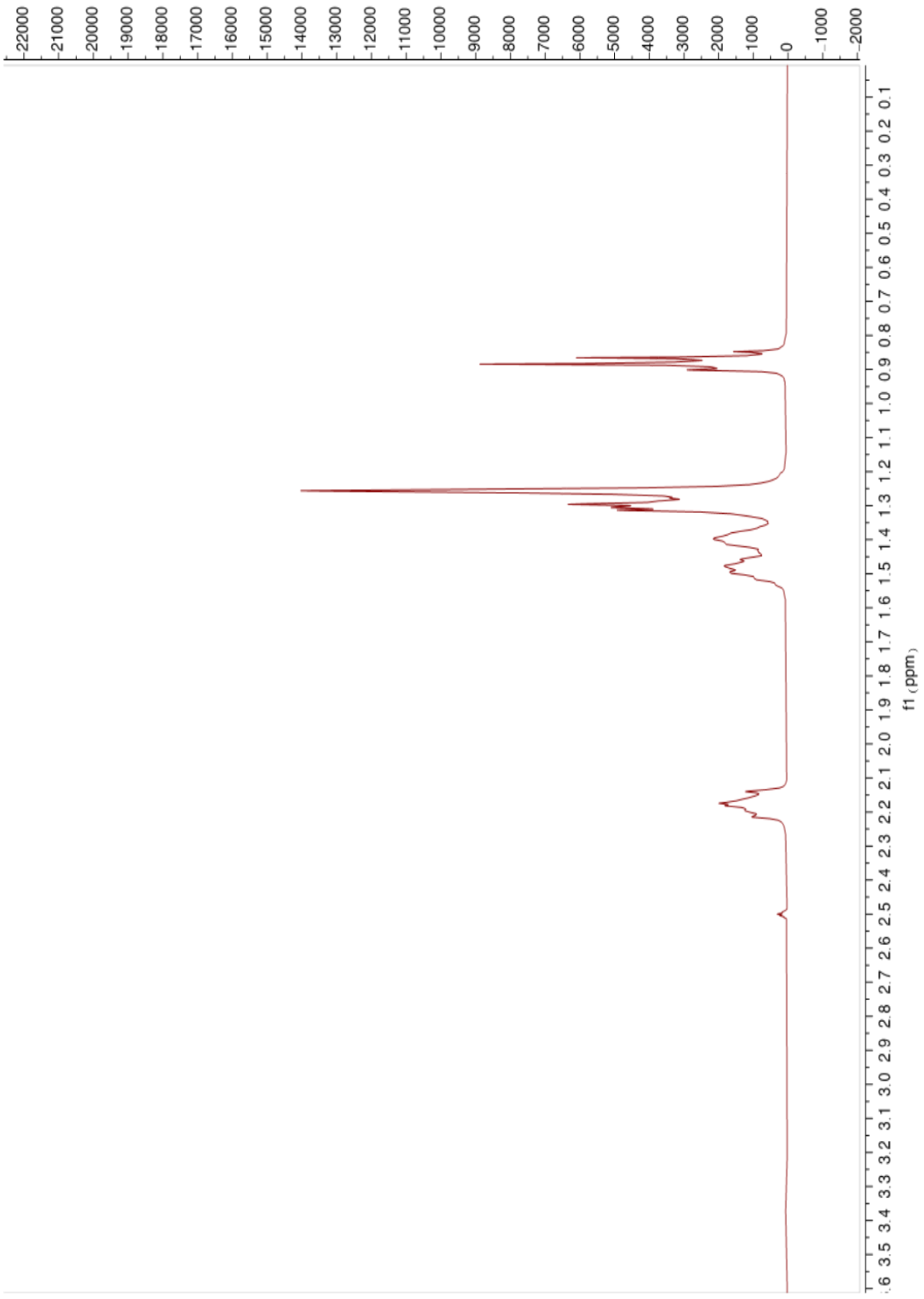

Supplementary Figure 1. ^{1}H NMR spectrum (DMSO-d_6, 400.13 MHz, 25 ˚C) of [$P_{666,14}$][TFSI]. Source data are provided as a Source Data file.

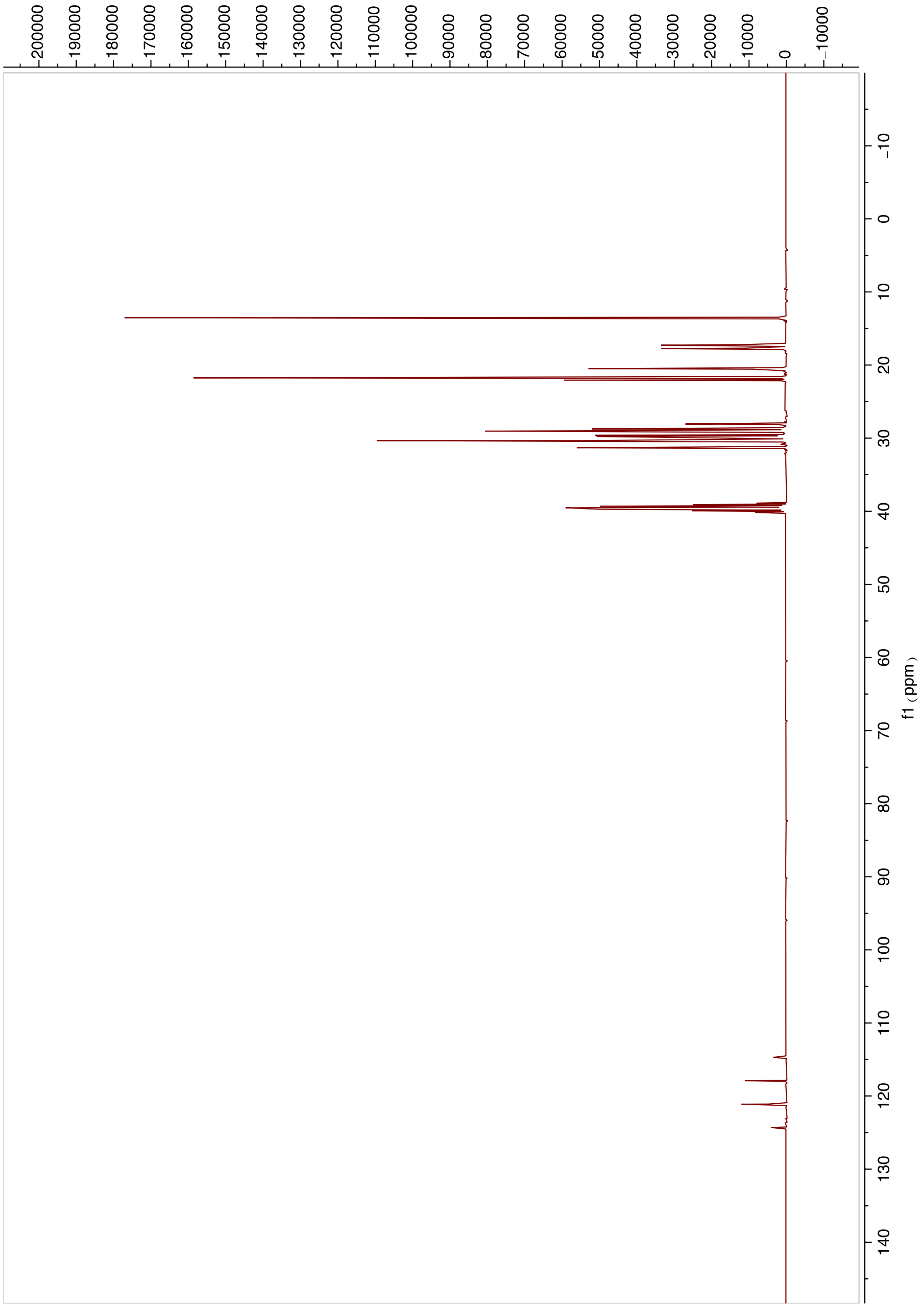

Supplementary Figure 2. ^{13}C NMR spectrum (d_6-DMSO, 100.61 MHz, 25 ˚C) of [$P_{666,14}$][TFSI] in d_6-DMSO. Source data are provided as a Source Data file.

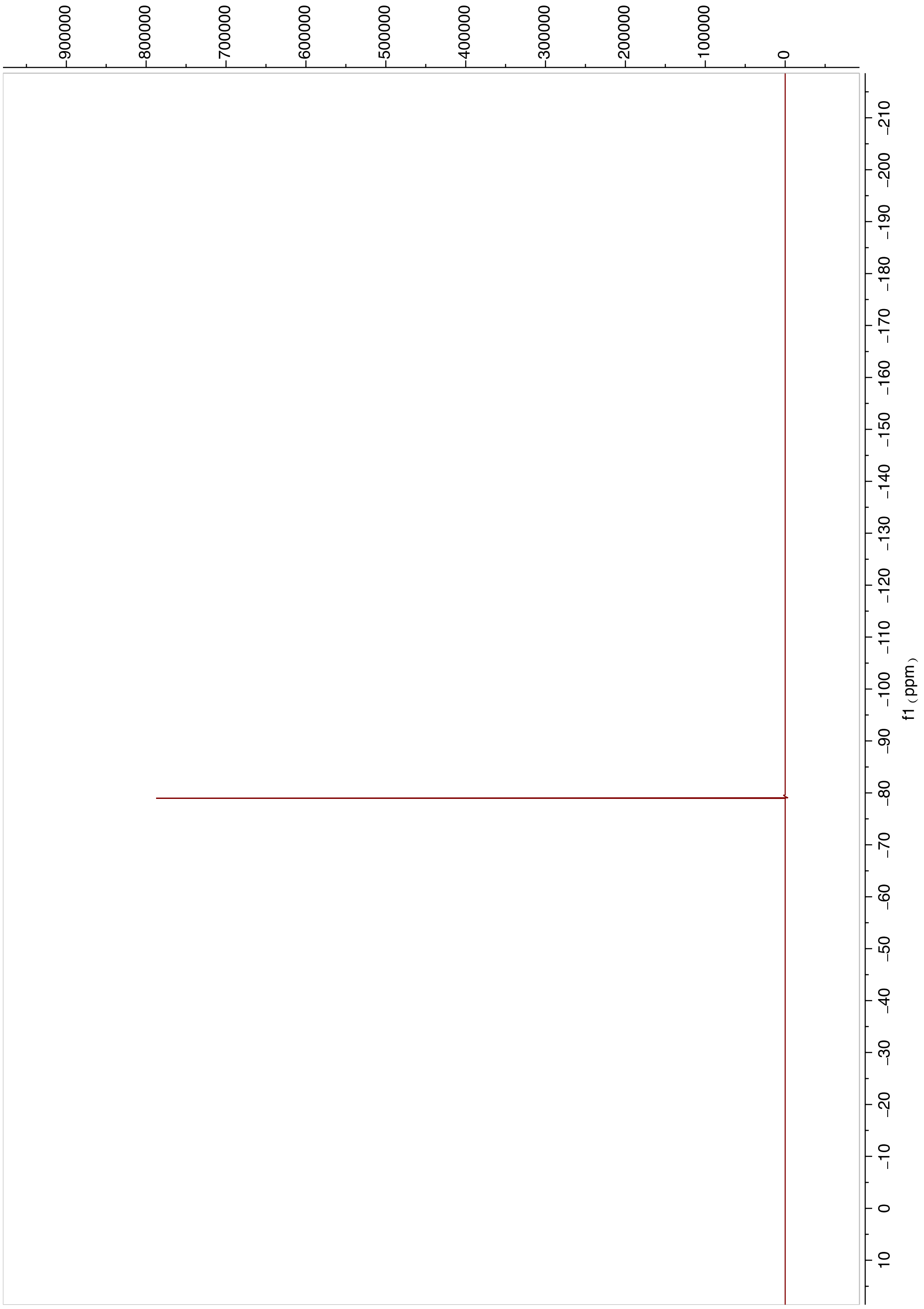

Supplementary Figure 3. ^{19}F NMR spectrum (d_6-DMSO, 376.50 MHz, 25 ˚C) of [$P_{666,14}$][TFSI]. Source data are provided as a Source Data file.

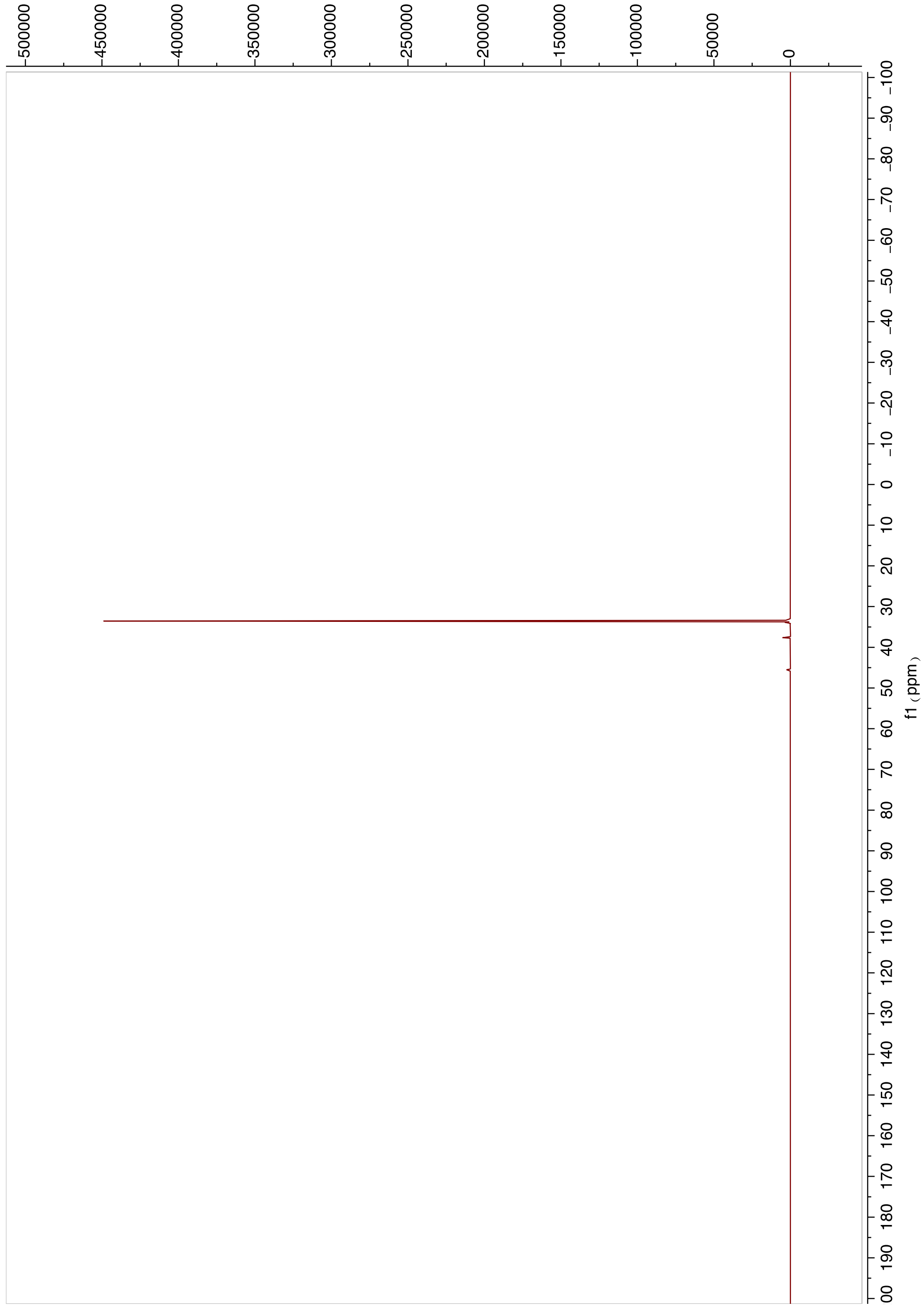

Supplementary Figure 4. ^{31}P NMR spectrum (d_6-DMSO, 161.98 MHz, 25 ˚C) of [$P_{666,14}$][TFSI]. Source data are provided as a Source Data file.

[$P_{666,14}$][SCN]. Trihexyl(tetradcyl)phosphonium chloride [$P_{666,14}$]Cl (0.010 mol eq.) and K[SCN] (0.013 mol eq.) were separately added to 25 cm^3 deionised water (18.2 MΩ.cm) deionised water (total 50 cm^3) and then combined in a round-bottomed flask (250 cm^3), resulting in the formation of a biphasic liquid system; the mixture was left to react (1 h, room temperature, 600 rpm). The aqueous layer was separated, and the organic layer was collected and washed, firstly with deionised water (10 cm^3) and then dichloromethane, DCM (10 cm^3). Subsequent washes were performed with solution of K[SCN] in deionised water (18.2 MΩ.cm). Final three washes were performed with deionised water (18.2 MΩ.cm) until no chloride could be detected with XRF. Subsequently, DCM was removed *via* rotary evaporation (30 min, 35 °C) and the ionic liquid was dried under high vacuum (12h, 70 °C, 10^{-2} mbar). XRF analysis of [$P_{666,14}$][SCN] confirmed chloride content was below the detectable limit. ^{1}H, ^{13}C and ^{31}P NMR spectra of the ionic liquid were recorded in d_6-DMSO.

^{1}H NMR (600.13 MHz, d_6 -DMSO) δ: 0.86-0.94 (m, 12H), 1.23-1.30 (m, 18H), 1.31-1.36 (m, 14H), 1.37-1.44 (m, 8H), 1.45-1.55 (m, 8H), 2.16-2.24 (m, 8H).

^{13}C{^{1}H}NMR (150.90 MHz, d_6 -DMSO) δ: 13.58 (s, P-$(CH_2)_5$-***CH_3***)., 13.60 (s, P-$(CH_2)_{13}$-***CH_3***), 18.63 (d, $^1J_{C/P}$ = 186 Hz, P-***CH_2***-$(CH_2)_4$-CH_3), 21.35 (s,), 22.01 (s,), 22.14 (s,), 22.27 (s,), 27.86 (d, $^2J_{C/P}$ = 198 Hz, P-CH_2-***CH_2***-$(CH_2)_3$-CH_3), 28.83 (d, $^2J_{C/P}$ = 198 Hz, P-CH_2-***CH_2***-$(CH_2)_{11}$-CH_3), 28.97 (s,), 29.28 (s, P-$(CH_2)_4$-***CH_2***-CH_3), 29.36 (s,), 30.08 (d, $^3J_{C/P}$ = 60 Hz, P-$(CH_2)_2$-***CH_2***-$(CH_2)_{10}$-CH_3), 30.43 (d, $^3J_{C/P}$ = 60 Hz, P-$(CH_2)_2$-***CH_2***-$(CH_2)_2$-CH_3), 30.70 (s, P-$(CH_2)_3$-***CH_2***-$(CH_2)_9$-CH_3), 31.31.09 (s, P-$(CH_2)_3$-***CH_2***-CH_2-CH_3), 31.54 (s,), 130.67 (s, S-***C***N).

^{31}P{^{1}H}NMR (242.938 MHz, d_6 -DMSO) δ: 32.58.

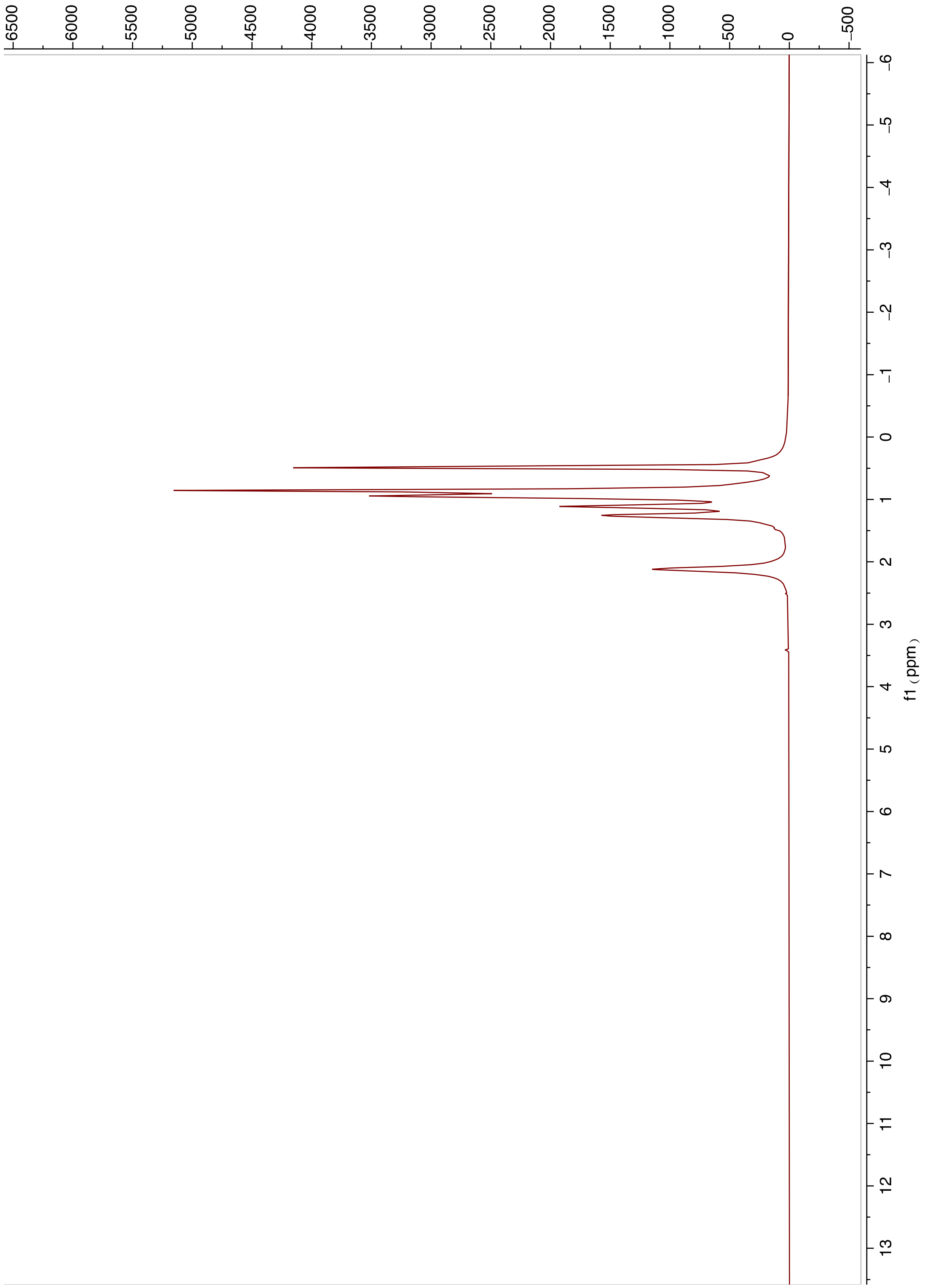

Supplementary Figure 5. ^{1}H NMR spectrum (d_6-DMSO, 600.13 MHz, 25 ˚C) of $[P_{666,14}][SCN]$. Source data are provided as a Source Data file.

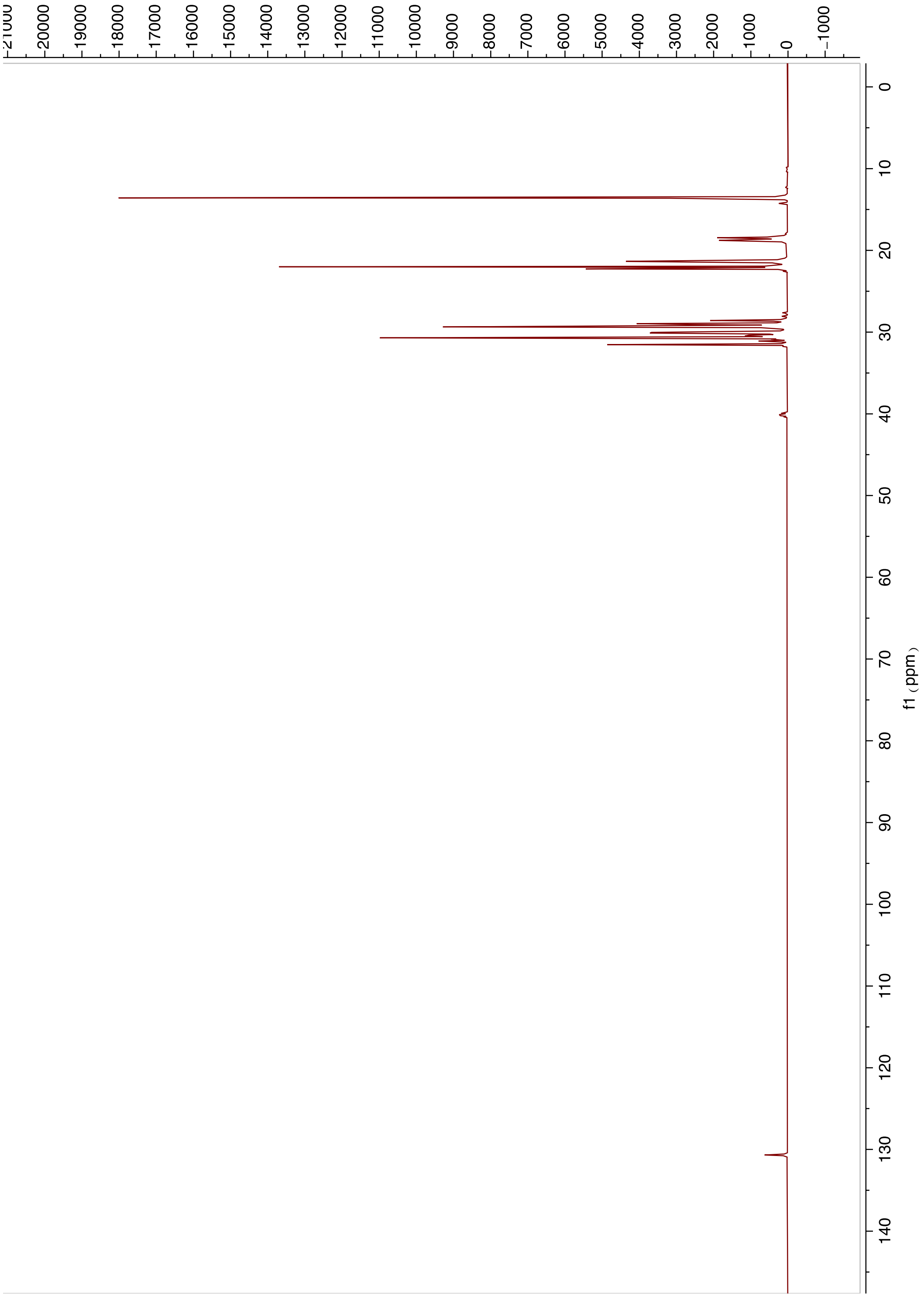

Supplementary Figure 6 . ^{13}C NMR spectrum (d_6-DMSO, 150.90 MHz, 25 ˚C) of [$P_{666,14}$][SCN]. Source data are provided as a Source Data file.

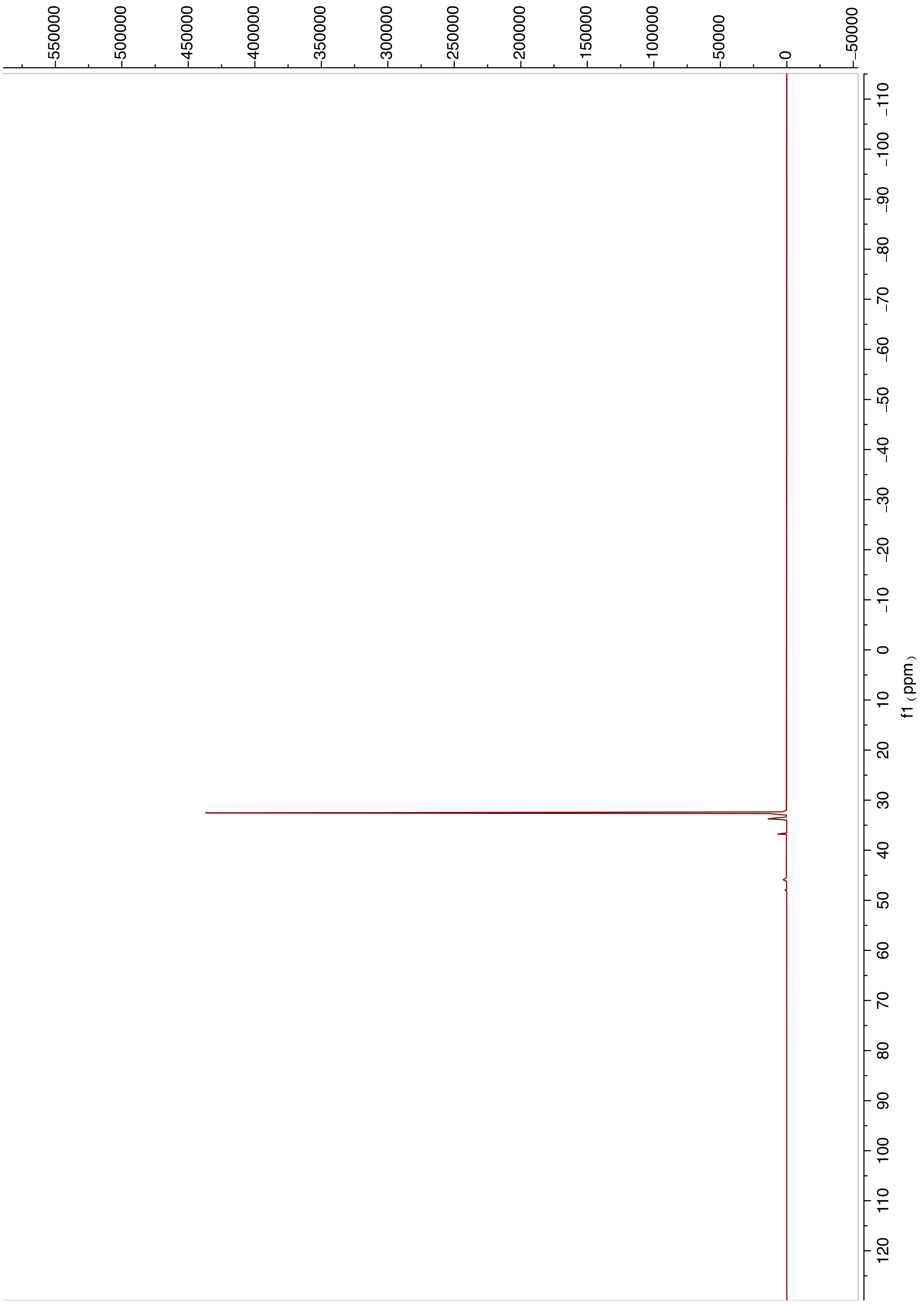

Supplementary Figure 7. ^{31}P NMR spectrum (d_6-DMSO, 242.94 MHz, 25 ˚C) of [$P_{666,14}$][SCN]. Source data are provided as a Source Data file.

[$P_{666,14}$][TCM]. Trihexyl(tetradecyl)phosphonium chloride [$P_{666,14}$]Cl (0.010 mol eq.) and sodium tricyanomethanide, Na[TCM] (0.013 mol eq.) were separately added to 25 cm^3 deionised water (18.2 MΩ.cm) (total 50 cm^3) and then combined in a round-bottomed flask (250 cm^3), resulting in the formation of a biphasic liquid system; the mixture was left to react (1 h, room temperature, 600 rpm). The aqueous layer was separated, and the organic layer was collected and washed, firstly with deionised water (18.2 MΩ.cm) (10 cm^3) and then dichloromethane, DCM (10 cm^3). Six subsequent washes were performed with solution of Na[TCM] in deionised water (18.2 MΩ.cm). Final three washes were performed with deionised water (18.2 MΩ.cm) until no chloride could be detected with silver nitrate solution. Subsequently, DCM was removed *via* rotary evaporation (30 min, 35 °C) and the ionic liquid was dried under high vacuum (12h, 70 °C, 10^{-2} mbar). XRF analysis of [$P_{666,14}$][TCM] recorded a chloride content of 89.3 ppm with a lower detection limit (LLD) of 2.80 ppm. ^{1}H, ^{13}C and ^{31}P NMR spectra of the ionic liquid were recorded in d_6-DMSO.

^{1}H NMR (600.13 MHz, d_6 -DMSO) δ: 0.84-0.93 (m, 12H), 1.21-1.28 (m, 18H), 1.30-1.35 (m, 14H), 1.35-1.42 (m, 8H), 1.43-1.54 (m, 8H), 2.14-2.23 (m, 8H).

^{13}C{^{1}H}NMR (150.90 MHz, d_6 -DMSO) δ: 4.87 (s, ***C***-$(CN)_3$),13.54 (s, P-$(CH_2)_5$-***CH_3***)., 13.68 (s, P-$(CH_2)_{13}$-***CH_3***), 18.28 (d, $^1J_{C/P}$ = 186 Hz, P-***CH_2***-$(CH_2)_4$-CH_3), 20.92 (s,), 20.95 (s,), 21.96 (s,), 22.34 (s,), 28.07 (s,), 28.63 (d, $^2J_{C/P}$ = 196 Hz, P-CH_2-***CH_2***-$(CH_2)_3$-CH_3), 29.12 (d, $^2J_{C/P}$ = 114 Hz, P-CH_2-***CH_2***-$(CH_2)_{11}$-CH_3), 29.33 (s,), 29.36 (s, P-$(CH_2)_4$-***CH_2***-CH_3), 29.38 (s,), 29.92 (d, $^3J_{C/P}$ = 60 Hz, P-$(CH_2)_2$-***CH_2***-$(CH_2)_{10}$-CH_3), 30.25 (d, $^3J_{C/P}$ = 54 Hz, P-$(CH_2)_2$-***CH_2***-$(CH_2)_2$-CH_3), 30.49 (s,), 31.06 (s, P-$(CH_2)_3$-***CH_2***-$(CH_2)_9$-CH_3), 31.59 (s, P-$(CH_2)_3$-***CH_2***-CH_2-CH_3), 120.83 (s, C-(***C***N)$_3$).

^{31}P{^{1}H}NMR (242.94 MHz, d_6 -DMSO) δ: 32.96.

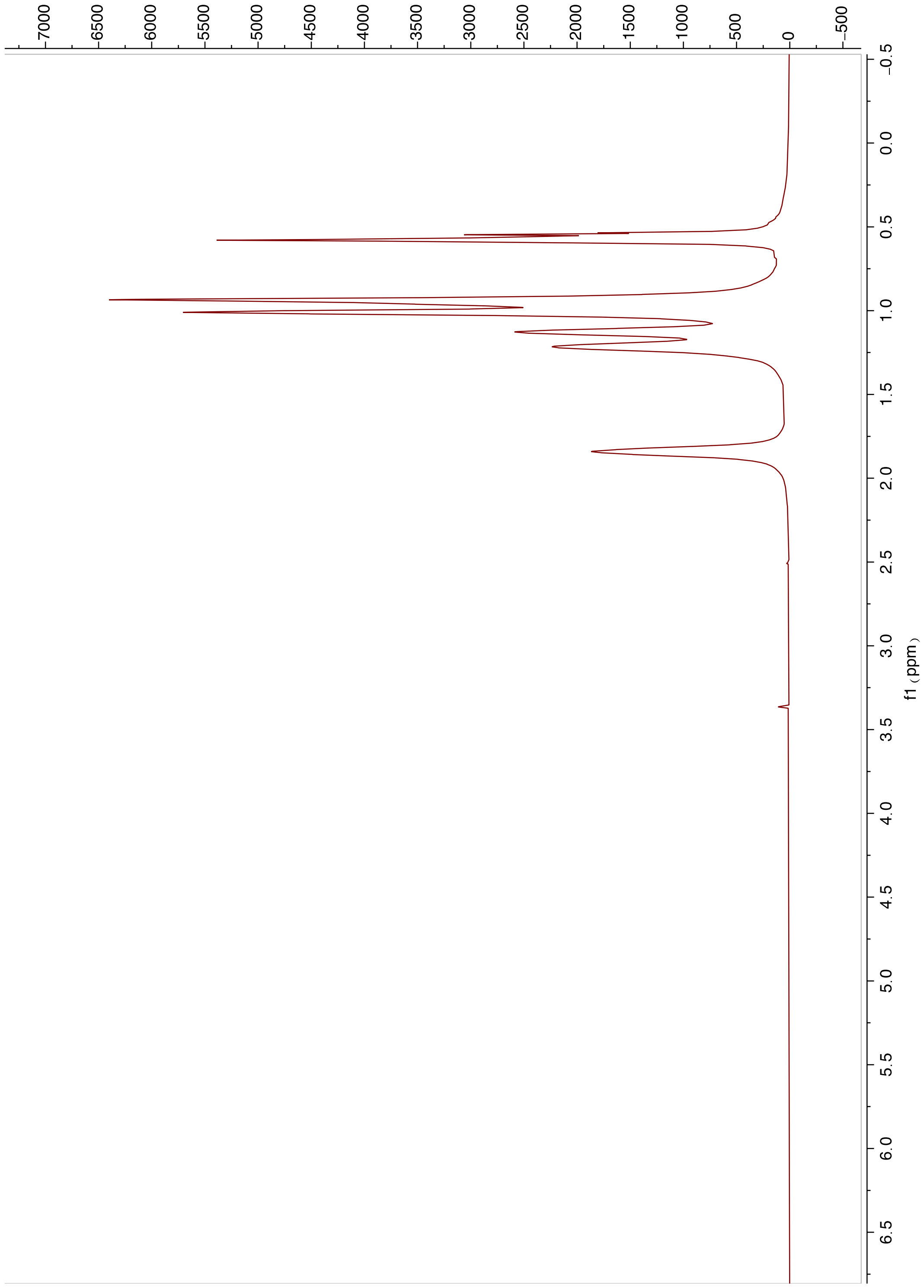

Supplementary Figure 8 ^{1}H NMR spectrum (d_6-DMSO, 600.13 MHz, 25 ˚C) of [$P_{666,14}$][TCM]. Source data are provided as a Source Data file.

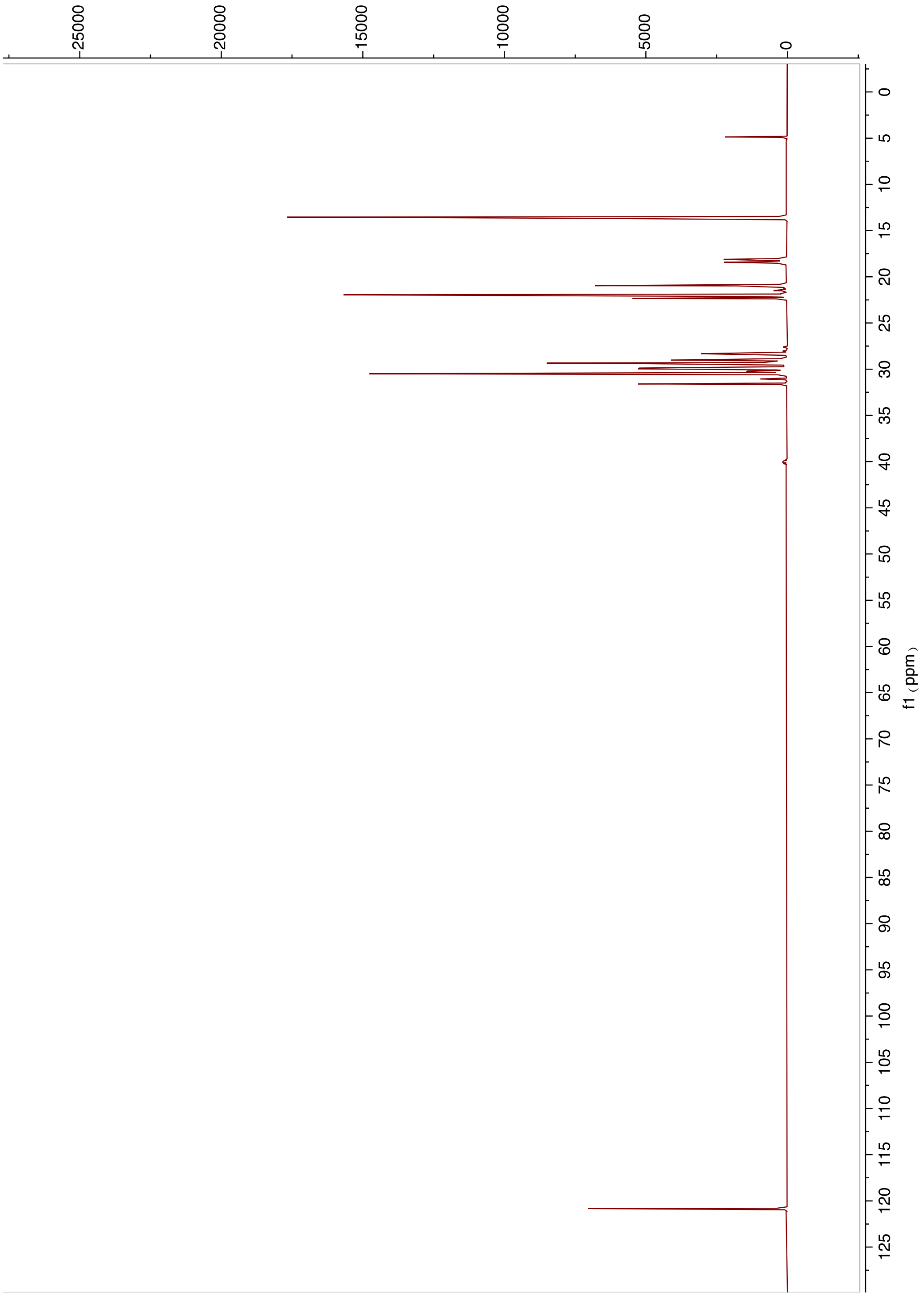

Supplementary Figure 9 ^{13}C NMR spectrum (d_6-DMSO, 150.90 MHz, 25 ˚C) of [$P_{666,14}$][TCM]. Source data are provided as a Source Data file.

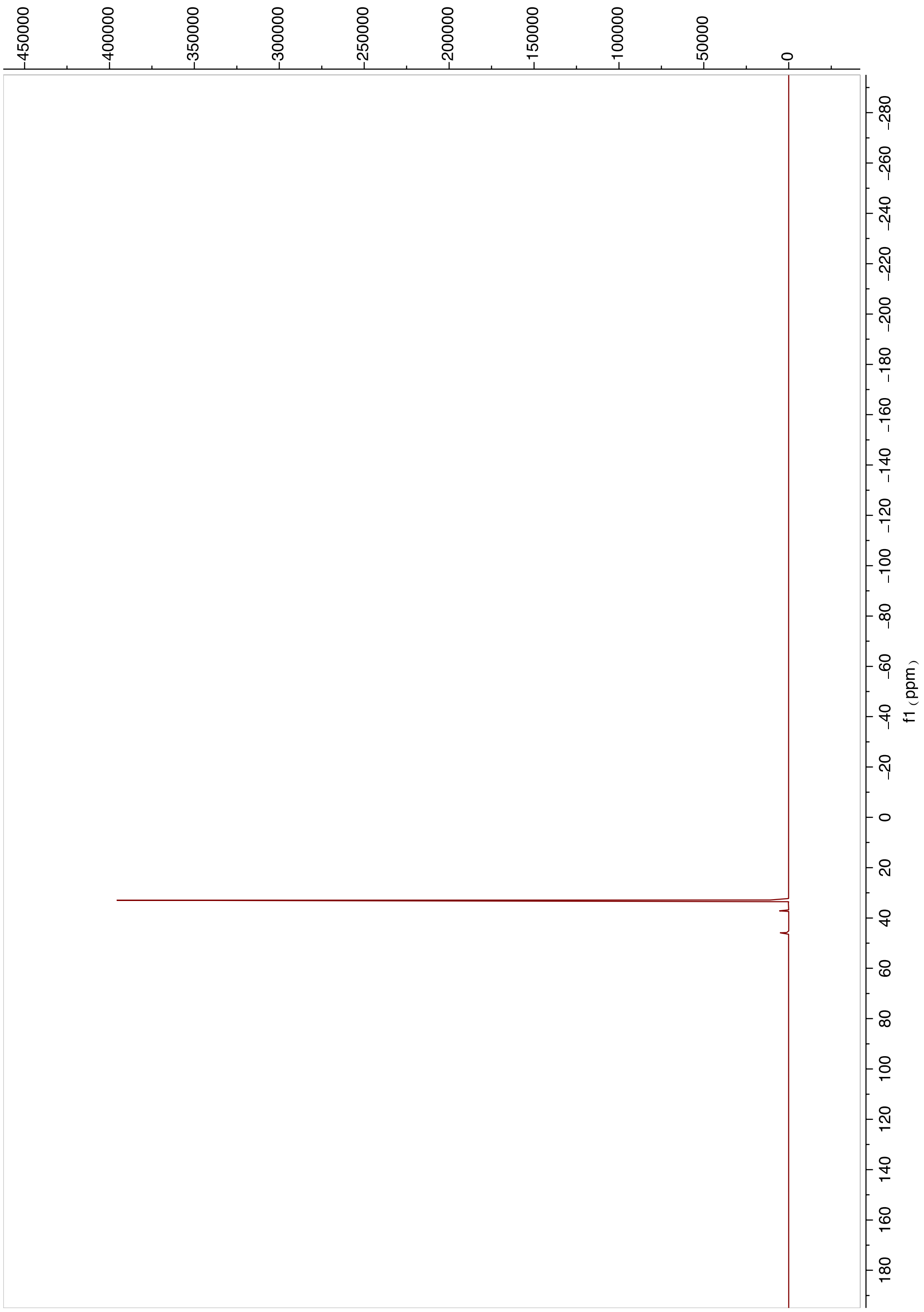

Supplementary Figure 10. ^{31}P NMR spectrum (d_6-DMSO, 242.94 MHz, 25 ˚C) of [$P_{666,14}$][TCM]. Source data are provided as a Source Data file.

[$P_{666,14}$][BF_4]. Trihexyl(tetradecyl)phosphonium chloride [$P_{666,14}$]Cl (0.010 mol eq.) and sodium tetrafluoroborate, Na[BF_4] (0.013 mol eq.) were separately added to 25 cm^3 deionised water (18.2 MΩ.cm) (total 50 cm^3) and then combined in a round-bottomed flask (250 cm^3), resulting in the formation of a biphasic liquid system; the mixture was left to react (1 h, room temperature, 600 rpm). The aqueous layer was separated, and the organic layer was collected and washed, firstly with deionised water (18.2 MΩ.cm) (10 cm^3) and then dichloromethane, DCM (10 cm^3). Subsequent washes were performed with solution of Na[BF_4] in deionised water (18.2 MΩ.cm). Final three washes were performed with deionised water (18.2 MΩ.cm) until no chloride could be detected with silver nitrate solution. Subsequently, DCM was removed *via* rotary evaporation (30 min, 35 °C) and the ionic liquid was dried under high vacuum (12h, 70 °C, 10^{-2} mbar). XRF analysis of [$P_{666,14}$][BF_4] recorded a chloride content of 69.0 ppm with a lower detection limit (LLD) of 2.35 ppm. ^{1}H, ^{13}C, ^{11}B and ^{31}P NMR spectra of the ionic liquid were recorded in d_6-DMSO.

^{1}H NMR (600.13 MHz, d_6 -DMSO) δ: 0.89-0.96 (m, 12H), 1.25-1.32 (m, 18H), 1.32-1.38 (m, 14H), 1.39-1.47 (m, 8H), 1.47-1.56 (m, 8H), 2.17-2.26 (m, 8H).

^{13}C{^{1}H}NMR (150.90 MHz, d_6 -DMSO) δ: 13.75 (s, P-$(CH_2)_5$-***CH_3***)., 13.83 (s, P-$(CH_2)_{13}$-***CH_3***), 17.36 (d, $^1J_{C/P}$ = 192 Hz, P-***CH_2***-$(CH_2)_4$-CH_3), 17.46 (d, $^1J_{C/P}$ = 186 Hz, P-***CH_2***-$(CH_2)_{12}$-CH_3), 20.47 (s,), 20.50 (s,), 21.77 (s,), 22.06 (s,), 28.07 (s,), 28.79 (d, $^2J_{C/P}$ = 186 Hz, P-CH_2-***CH_2***-$(CH_2)_3$-CH_3), 28.86 (d, $^2J_{C/P}$ = 186 Hz, P-CH_2-***CH_2***-$(CH_2)_{11}$-CH_3), 28.99 (s,), 29.03 (s, P-$(CH_2)_4$-***CH_2***-CH_3), 29.05 (s,), 29.70 (d, $^3J_{C/P}$ = 60 Hz, P-$(CH_2)_2$-***CH_2***-$(CH_2)_{10}$-CH_3), 29.93 (d, $^3J_{C/P}$ = 60 Hz, P-$(CH_2)_2$-***CH_2***-$(CH_2)_2$-CH_3), 30.36 (s, P-$(CH_2)_3$-***CH_2***-$(CH_2)_9$-CH_3), 31.28 (s, P-$(CH_2)_3$-***CH_2***-CH_2-CH_3).

^{11}B NMR (64.48 MHz, d_6 -DMSO) δ: -1.26.

^{19}F NMR (564.69 MHz, d_6 -DMSO) δ: -148.42.

^{31}P{^{1}H}NMR (242.94 MHz, d_6 -DMSO) δ: 33.70.

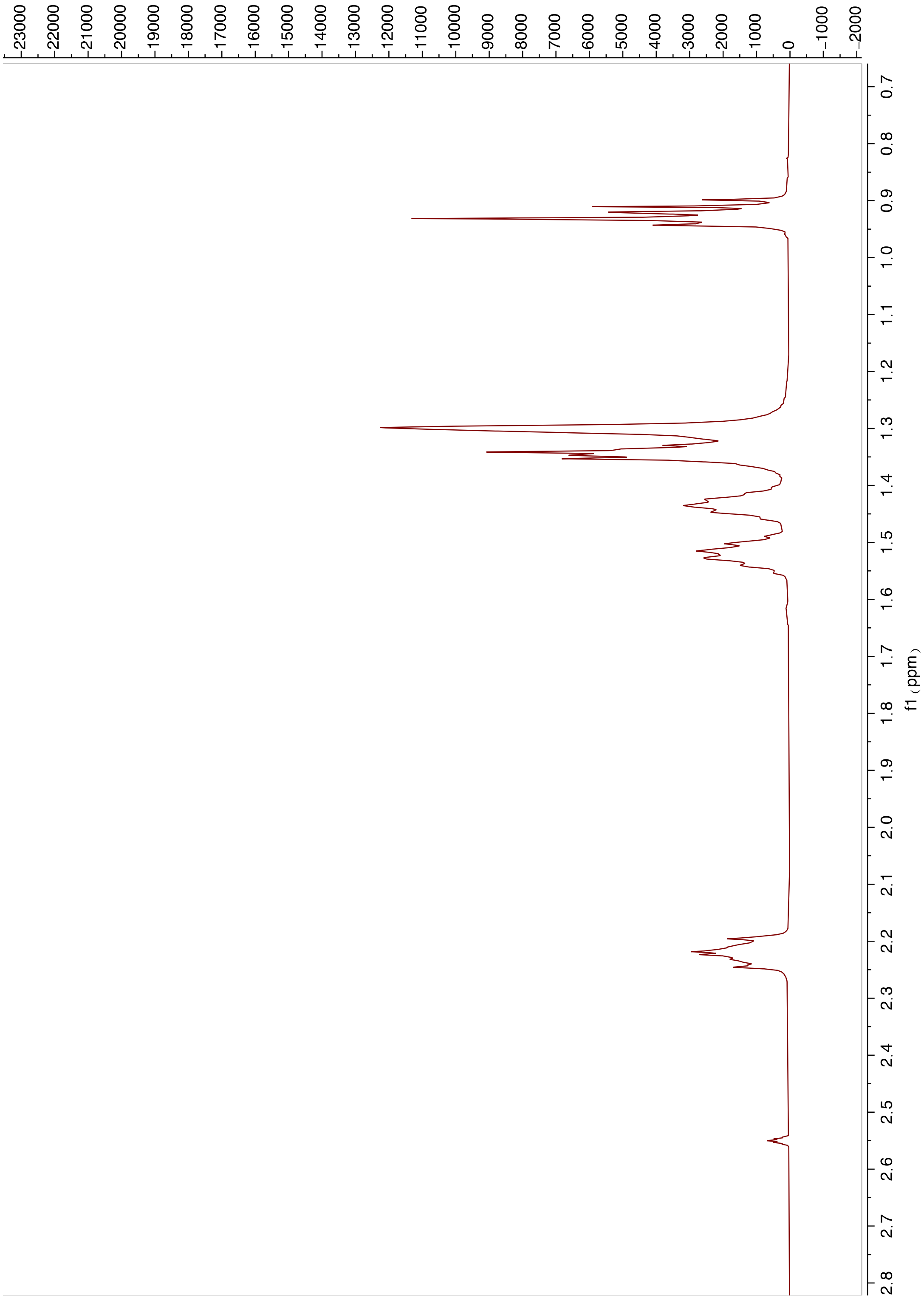

Supplementary Figure 11 ^{1}H NMR spectrum (d_6-DMSO, 600.13 MHz, 25 ˚C) of [$P_{666,14}$][BF_4]. Source data are provided as a Source Data file.

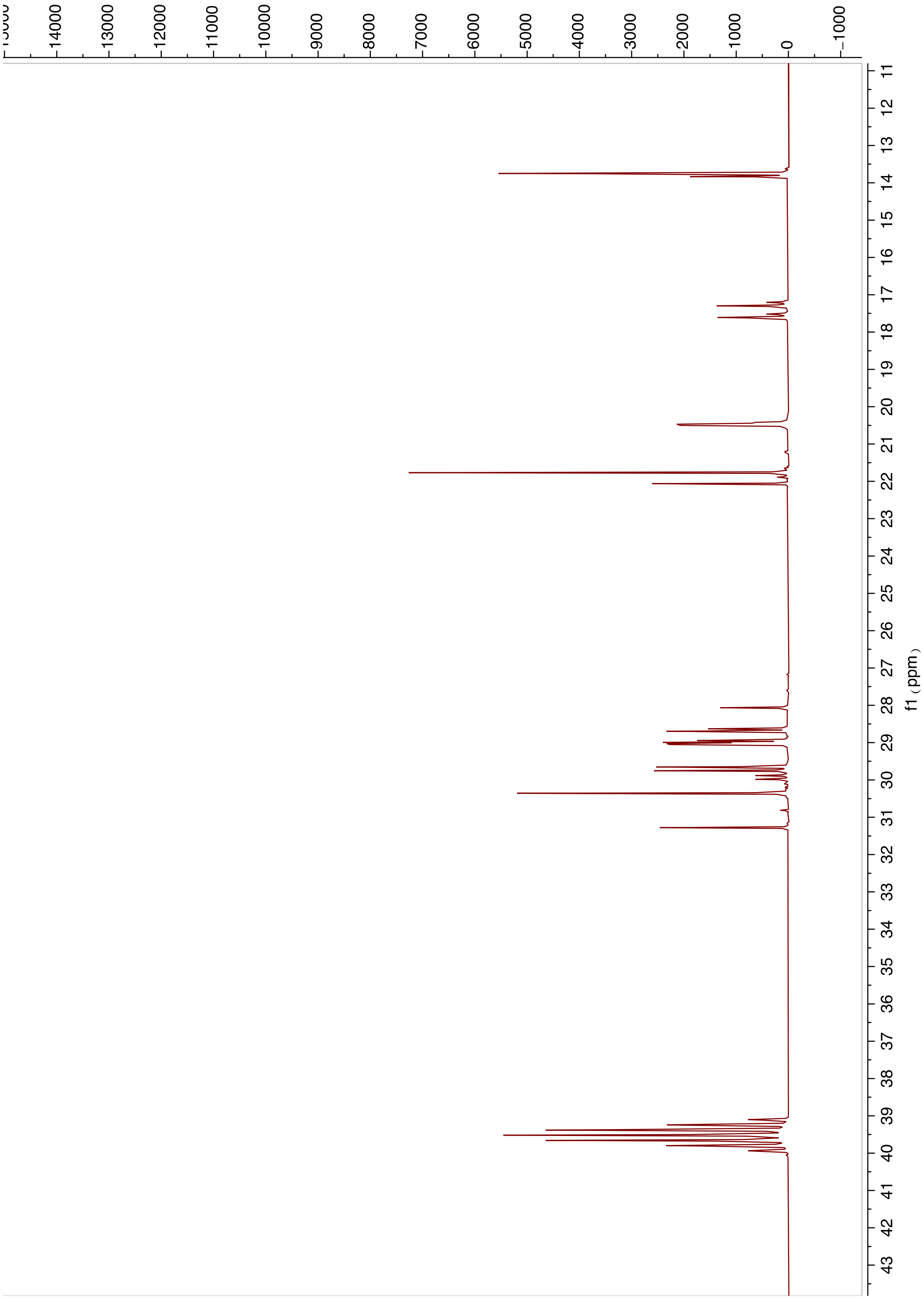

Supplementary Figure 12 ^{13}C NMR spectrum (d_6-DMSO, 150.90 MHz, 25 ˚C) of [$P_{666,14}$][BF_4]. Source data are provided as a Source Data file.

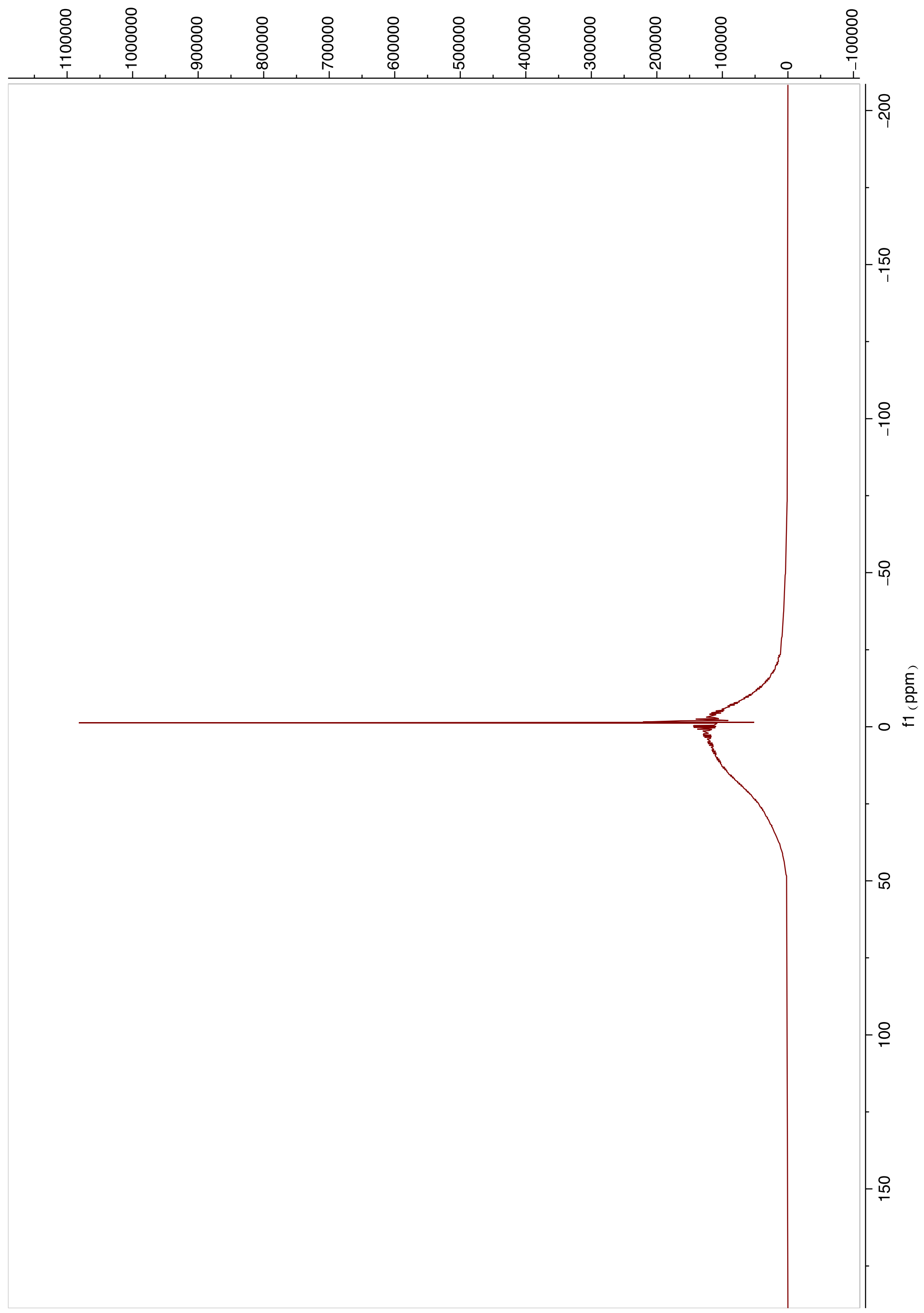

Supplementary Figure 13 ^{11}B NMR spectrum (d_6-DMSO, 64.48 MHz, 25 ˚C) of [$P_{666,14}$][BF_4]. Source data are provided as a Source Data file.

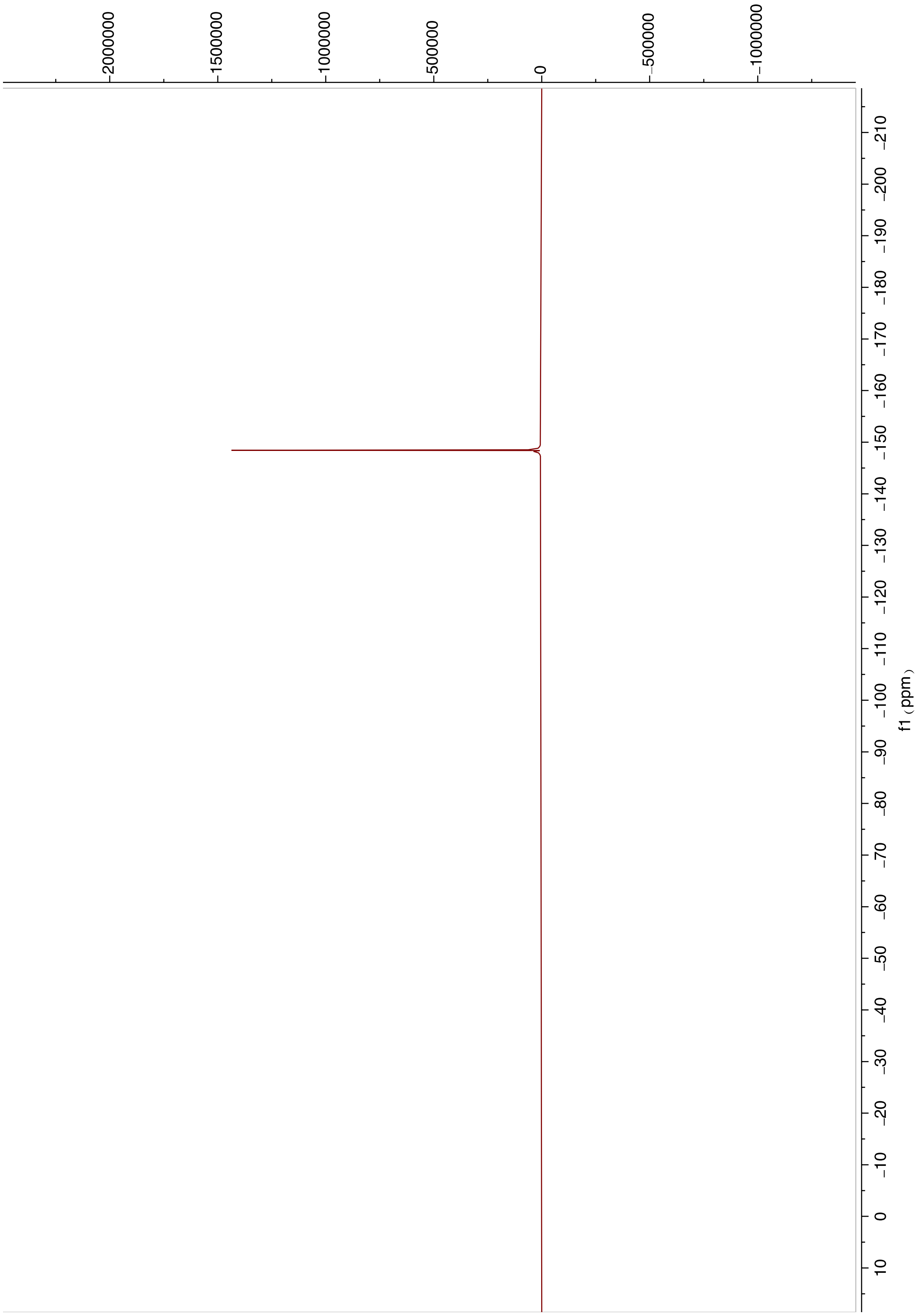

Supplementary Figure 14 ^{19}F NMR spectrum (d_6-DMSO, 564.69 MHz, 25 ˚C) of [$P_{666,14}$][BF_4]. Source data are provided as a Source Data file.

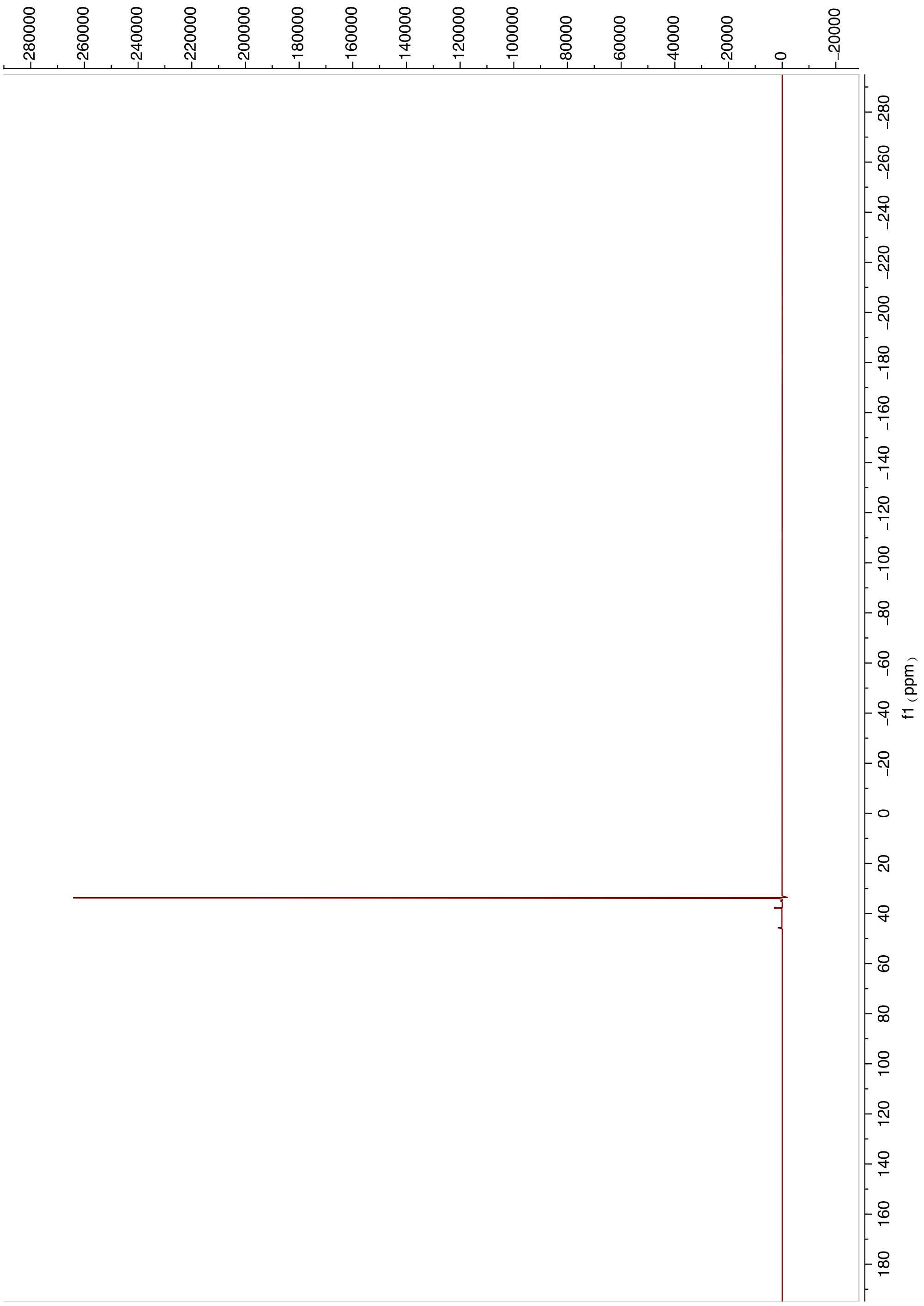

Supplementary Figure 15. ^{31}P NMR spectrum (d_6-DMSO, 242.94 MHz, 25 ˚C) of [$P_{666,14}$][BF_4]. Source data are provided as a Source Data file.

[$P_{666,14}$][TAU]. Trihexyl(tetradecyl)phosphonium chloride [$P_{666,14}$]Cl was converted to [$P_{666,14}$][OH] (solution in methanol) following a literature procedure.[1] [$P_{666,14}$][TAU] was synthesised following a modified literature procedure.[2] Taurine (0.012 mol eq.) was dissolved in 35 cm^3 of deionised water (18.2 MΩ.cm) and [$P_{666,14}$][OH] (0.015 mol eq., solution in methanol) was added. The solution was stirred for 2 h at ambient temperature and the water-methanol mixture was removed *via* rotary evaporation (3 h, 30-80 °C). Crude product was dissolved in 50 ml of dry acetonitrile and stored in the fridge (5 °C) for 12h. The excess taurine crystallised and was filtered off. Acetonitrile was removed *via* rotary evaporation (30 min, 60 °C) and the ionic liquid was dried under high vacuum (overnight, 70 °C, 10^{-2} mbar). XRF analysis of [$P_{666,14}$][TAU] confirmed chloride content was below the detectable limit. ^{1}H, ^{13}C, ^{11}B and ^{31}P NMR spectra of the ionic liquid were recorded in d_6-DMSO.

^{1}H NMR (600.13 MHz, d_6 -DMSO) δ: 0.83-0.92 (m, 12H) 1.19-1.28 (m, 18H), 1.28-1.35 (m, 14H), 1.35-1.44 (m, 8H), 1.44-1.54 (m, 8H), 2.20-2.30 (m, 8H), 2.47-2.53 (t, 2H), 2.77-2.83 (t, 2H).

^{13}C{^{1}H}NMR (150.90 MHz, d_6 -DMSO) δ: 13.61 (s, P-$(CH_2)_5$-***CH_3***)., 13.66 (s, P-$(CH_2)_{13}$-***CH_3***), 17.39 (d, $^{1}J_{C/P}$ = 186 Hz, P-***CH_2***-$(CH_2)_4$-CH_3), 17.47 (d, $^{1}J_{C/P}$ = 192 Hz, P-***CH_2***-$(CH_2)_{12}$-CH_3), 20.56 (s,), 20.58 (s,), 21.75 (s,), 22.03 (s,), 28.13 (s,), 28.85 (d, $^{2}J_{C/P}$ = 204 Hz, P-CH_2-***CH_2***-$(CH_2)_3$-CH_3), 28.96 (s,), 29.01 (d, $^{2}J_{C/P}$ = 36 Hz, P-CH_2-***CH_2***-$(CH_2)_{11}$-CH_3), (s, P-$(CH_2)_4$-***CH_2***-CH_3), (s,), 29.71 (d, $^{3}J_{C/P}$ = 60 Hz, P-$(CH_2)_2$-***CH_2***-$(CH_2)_{10}$-CH_3), 29.97 (d, $^{3}J_{C/P}$ = 60 Hz, P-$(CH_2)_2$-***CH_2***-$(CH_2)_2$-CH_3), 30.37 (s, P-$(CH_2)_3$-***CH_2***-$(CH_2)_9$-CH_3), 31.26 (s, P-$(CH_2)_3$-***CH_2***-CH_2-CH_3) 38.52 (s, SO_3-CH_2-***CH_2***-NH_2), 54.76 (s, SO_3-***CH_2***-CH_2-NH_2).

^{31}P{^{1}H}NMR (242.94 MHz, d_6 -DMSO) δ: 33.53.

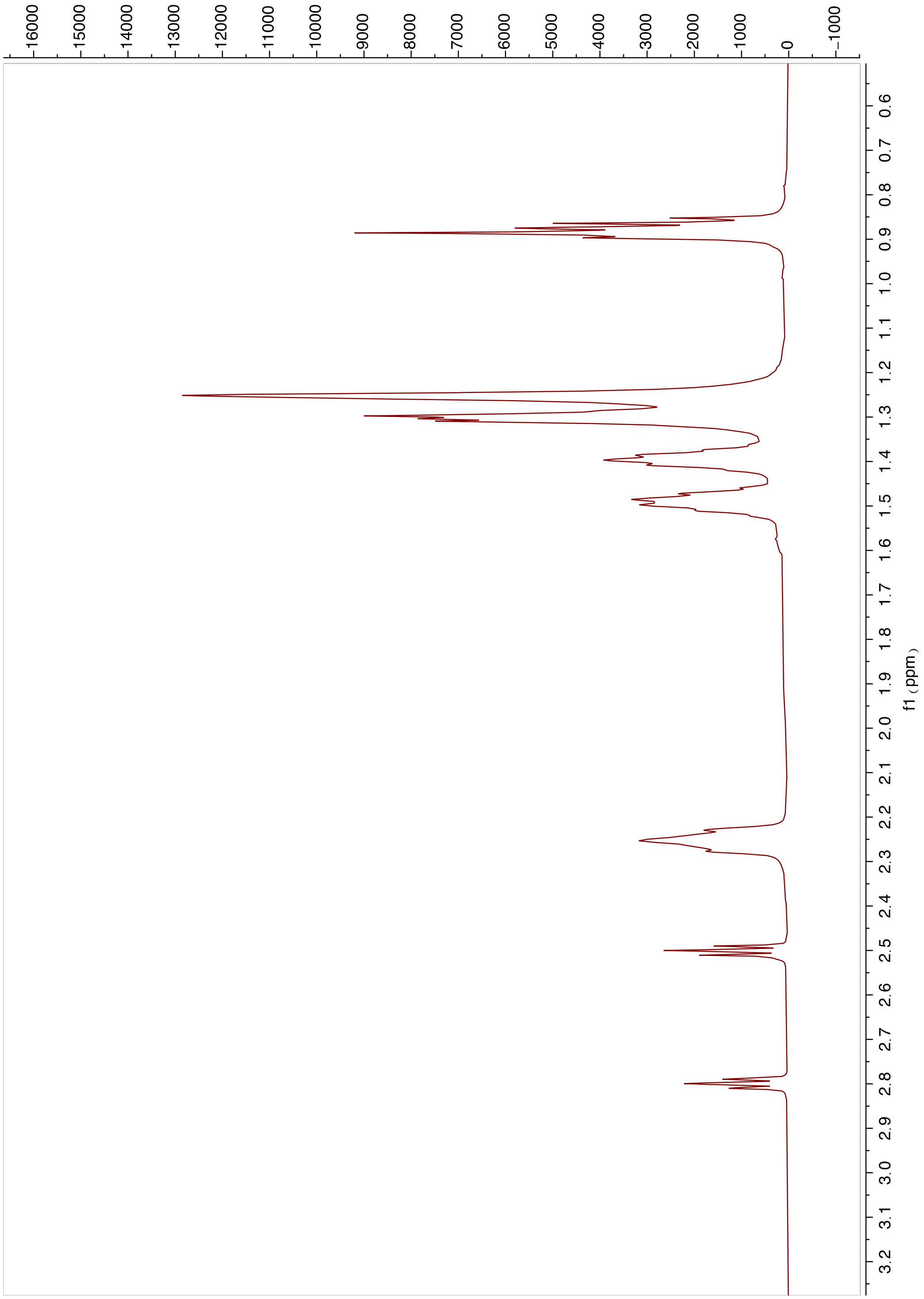

Supplementary Figure 16 ^{1}H NMR spectrum (d_6-DMSO, 600.13 MHz, 25 ˚C) of [$P_{666,14}$][TAU]. Source data are provided as a Source Data file.

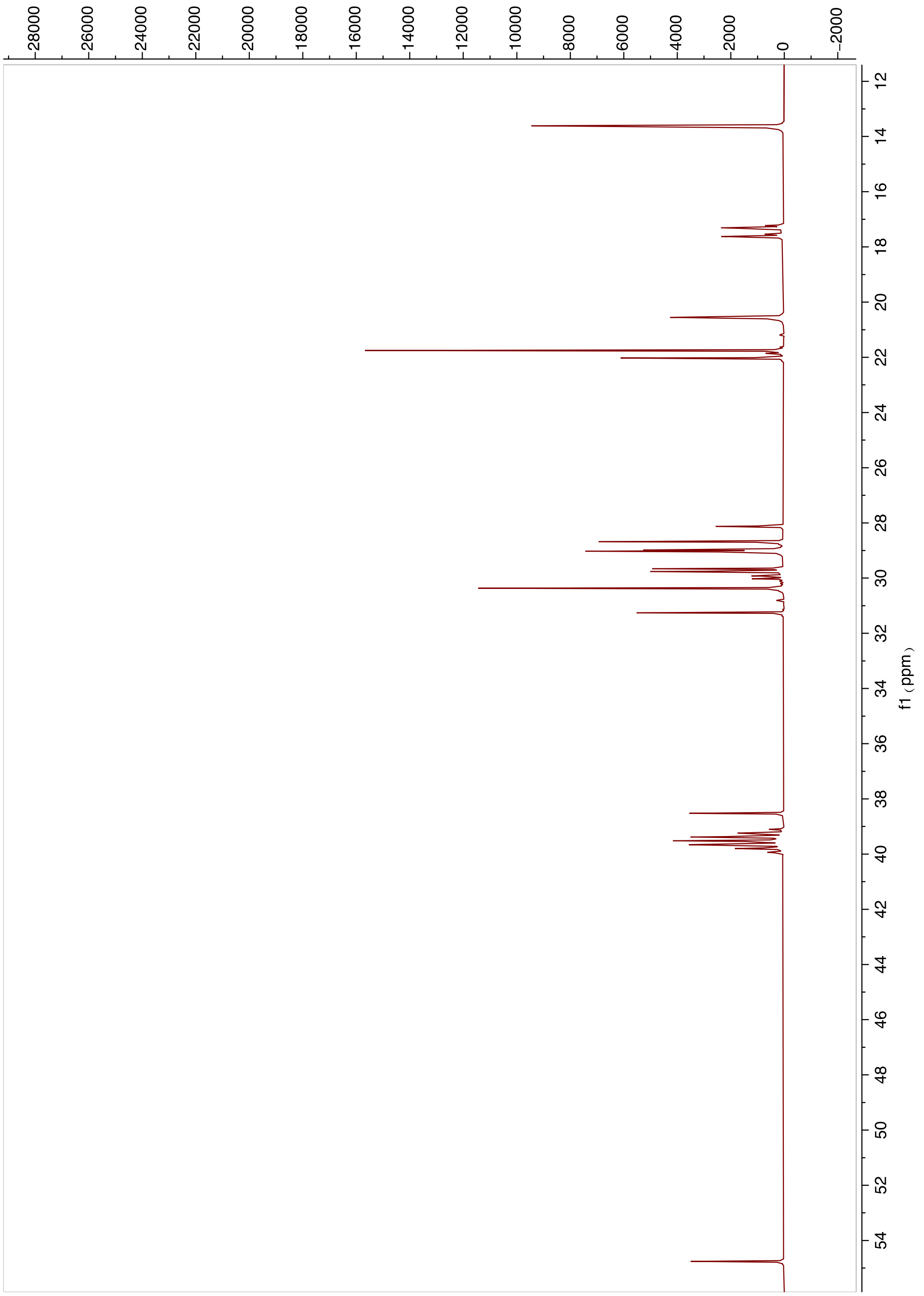

Supplementary Figure 17 ^{13}C NMR spectrum (d_6-DMSO, 150.90 MHz, 25 ˚C) of $[P_{666,14}]$[TAU]. Source data are provided as a Source Data file.

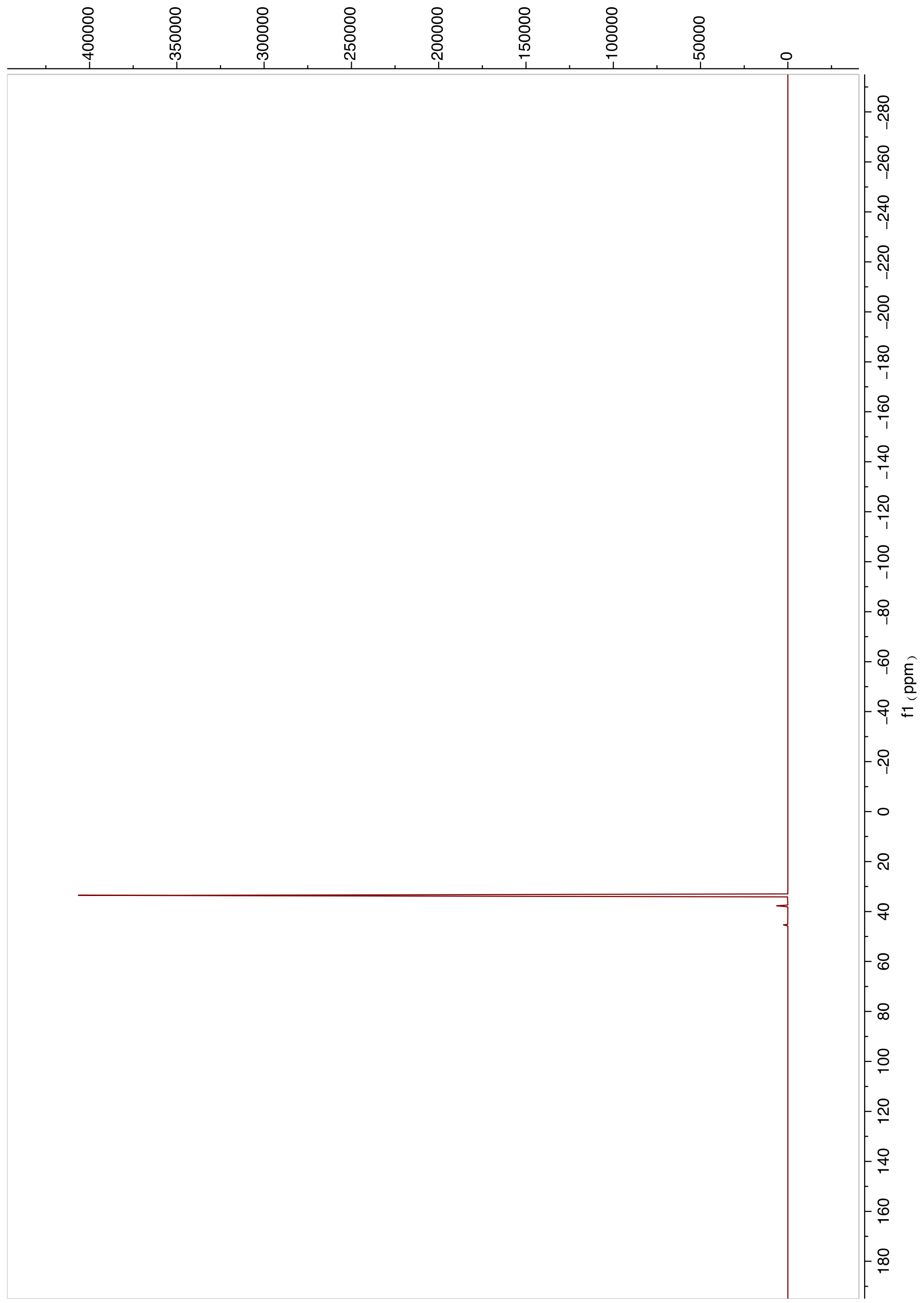

Supplementary Figure 18. ^{31}P NMR spectrum (d_6-DMSO, 242.94 MHz, 25 ˚C) of $[P_{666,14}]$[TAU]. Source data are provided as a Source Data file.

[$P_{666,14}$][BH_4]. Trihexyl(tetradecyl)phosphonium chloride [$P_{666,14}$]Cl (0.10 mol eq.) and sodium borohydride, Na[BH_4] (0.13 mol eq.) were separately added to 150 cm^3 of deionised water (18.2 MΩ.cm). Both solutions were combined (total of 300 cm^3) in a round-bottomed flask, resulting in the formation of a biphasic liquid system. The solution was left to react (48 h, room temperature, 600 rpm). The ionic liquid layer was dissolved in 300 cm^3 of chloroform ($CHCl_3$) and collected using a separating funnel. The dense chloroform layer was washed ten times with 100 cm^3 of salt solution (1.0 equivalent of $NaBH_4$ in 1000 cm^3 of deionised water (18.2 MΩ.cm)). Chloroform was removed *via* rotary evaporation (30 min, 35 °C) and the solution was dried overnight under high vacuum (12 h, 70 °C, 10^{-2} mbar). XRF analysis of [$P_{666,14}$][BH_4] recorded a chloride content of 47900 ppm with a lower detection limit (LLD) of 3.57 ppm. Chloride removal was carried out in three subsequent polishing steps.

Firstly, ionic liquid [$P_{666,14}$][BH_4] was redissolved in 300 cm^3 of chloroform and additional washes were performed to lower the chloride content. 1.0 equivalent of $NaBH_4$ was dissolved in 150 cm^3 of deionised water (18.2 MΩ.cm) and added to the solution. The reaction mixture was stirred for 4 h, then allowed to stand for 30 min for chloroform–water phase separation. Centrifugation was performed to encourage phase disentanglement. The dense chloroform layer was washed seven times with 100 cm^3 of deionised water (18.2 MΩ.cm) to remove traces of NaCl by-product residing within the organic layer. Chloroform was removed *via* rotary evaporation (30 min, 35 °C) and the solution was dried overnight under high vacuum (12 h, 70 °C, 10^{-2} mbar). XRF analysis of [$P_{666,14}$][BH_4] recorded a chloride content of 9270 ppm with a lower detection limit (LLD) of 3.97 ppm.

Secondly, the ionic liquid [$P_{666,14}$][BH_4] was again redissolved in 300 cm^3 of chloroform and additional washes were performed to lower the chloride content. 1.0 equivalent of $NaBH_4$ was dissolved in 150 cm^3 of deionised water (18.2 MΩ.cm) and added to the solution. The reaction mixture was stirred for 4 h, then allowed to stand for an additional 30 min. Centrifugation was performed to encourage phase disentanglement. The dense chloroform layer was washed seven times with 100 cm^3 of deionised water (18.2 MΩ.cm). Chloroform was removed *via* rotary evaporation (30 min, 35 °C) and the solution was dried overnight under high vacuum (12 h, 70 °C, 10^{-2} mbar). XRF analysis of [$P_{666,14}$][BH_4] recorded a chloride content of 7230 ppm with a lower detection limit (LLD) of 3.96 ppm.

In the final step, the ionic liquid [$P_{666,14}$][BH_4] was redissolved in 300 cm^3 of chloroform and additional washes were performed. 1.0 equivalent of $NaBH_4$ was dissolved in 150 cm^3 of deionised water (18.2 MΩ.cm) and added to the solution. The reaction mixture was stirred for 4 h and allowed to stand for 30 min. The dense chloroform layer was washed seven times with 100 cm^3 of salt solution (1.0 equivalent of $NaBH_4$ in 700 cm^3 of deionised water (18.2 MΩ.cm). Final three washes were performed with 100 cm^3 of deionised water (18.2 MΩ.cm). Chloroform was removed *via* rotary evaporation (30 min, 35 °C) and the ionic liquid was dried overnight under high vacuum (12 h, 70 °C, 10^{-2} mbar). XRF analysis of [$P_{666,14}$][BH_4] was recorded three times to give an average chloride content of 1326 ppm with an average lower detection limit (LLD) of 3.49 ppm. ^{1}H, ^{13}C, ^{11}B and ^{31}P NMR spectra of the ionic liquid were recorded in d_6-DMSO.

^{1}H NMR (600.13 MHz, d_6-DMSO) δ: -0.72-(-0.27) (q, 4H) 0.44-0.60 (m, 12H), 0.60-0.92 (m, 18H), 0.92-1.01 (m, 14H), 1.04-1.16 (m, 8H), 1.17-1.38 (m, 8H), 1.92-2.48 (m, 8H).

$^{13}C\{^{1}H\}$ NMR (150.90 MHz, *d*$_6$-DMSO) δ: 13.15 (s, P-$(CH_2)_5$-***CH_3***), 13.18 (s, P-$(CH_2)_{13}$-***CH_3***), 18.07 (d, $^{1}J_{C/P}$ = 186 Hz, P-***CH_2***-$(CH_2)_4$-CH_3 and P-***CH_2***-$(CH_2)_{12}$-CH_3), 20.98 (s, P-$(CH_2)_{12}$-***CH_2***-CH_3), 21.06 (s, P-$(CH_2)_{11}$-***CH_2***-CH_2-CH_3), 21.62 (s, P-$(CH_2)_{10}$-***CH_2***-$(CH_2)_2$-CH_3), 21.75 (s, P-$(CH_2)_9$-***CH_2***-$(CH_2)_3$-CH_3), 21.84 (s, P-$(CH_2)_8$-***CH_2***-$(CH_2)_4$-CH_3), 27.54 (d, $^{2}J_{C/P}$ = 258 Hz, P-CH_2-***CH_2***-$(CH_2)_3$-CH_3), 28.29 (s, P-$(CH_2)_7$-***CH_2***-$(CH_2)_5$-CH_3), 28.57 (s, P-$(CH_2)_6$-***CH_2***-$(CH_2)_6$-CH_3), 28.75 (s, P-$(CH_2)_5$-***CH_2***-$(CH_2)_7$-CH_3), 28.87 (s, P-$(CH_2)_4$-***CH_2***-$(CH_2)_8$-CH_3), 28.96 (s, P-$(CH_2)_4$-***CH_2***-CH_3), 29.64 (d, $^{2}J_{C/P}$ = 54 Hz, P-CH_2-***CH_2***-$(CH_2)_{11}$-CH_3), 30.00 (d, $^{3}J_{C/P}$ = 60 Hz, P-$(CH_2)_2$-***CH_2***-$(CH_2)_{10}$-CH_3), 30.17 (d, $^{3}J_{C/P}$ = 54 Hz, P-$(CH_2)_2$-***CH_2***-$(CH_2)_2$-CH_3), 30.36 (s, P-$(CH_2)_3$-***CH_2***-$(CH_2)_9$-CH_3), 31.11 (s, P-$(CH_2)_3$-***CH_2***-CH_2-CH_3).

^{11}B NMR (64.48 MHz, *d*$_6$-DMSO) δ: -36.98 (p).

$^{31}P\{^{1}H\}$NMR (242.94 MHz, *d*$_6$-DMSO) δ: 32.72.

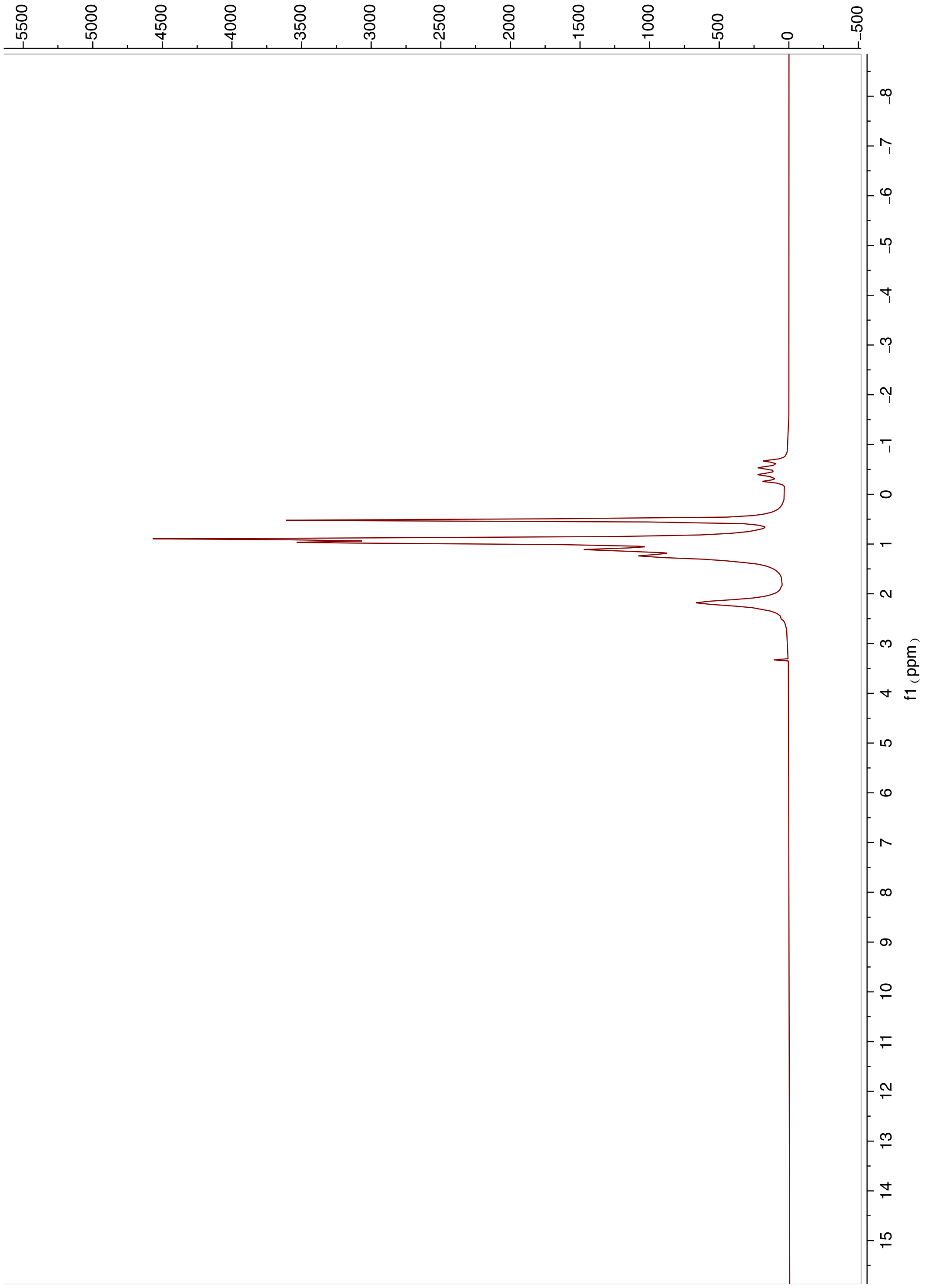

Supplementary Figure 19 ^{1}H NMR spectrum (d_6-DMSO, 600.13 MHz, 25 ˚C) of $[P_{666,14}][BH_4]$. Source data are provided as a Source Data file.

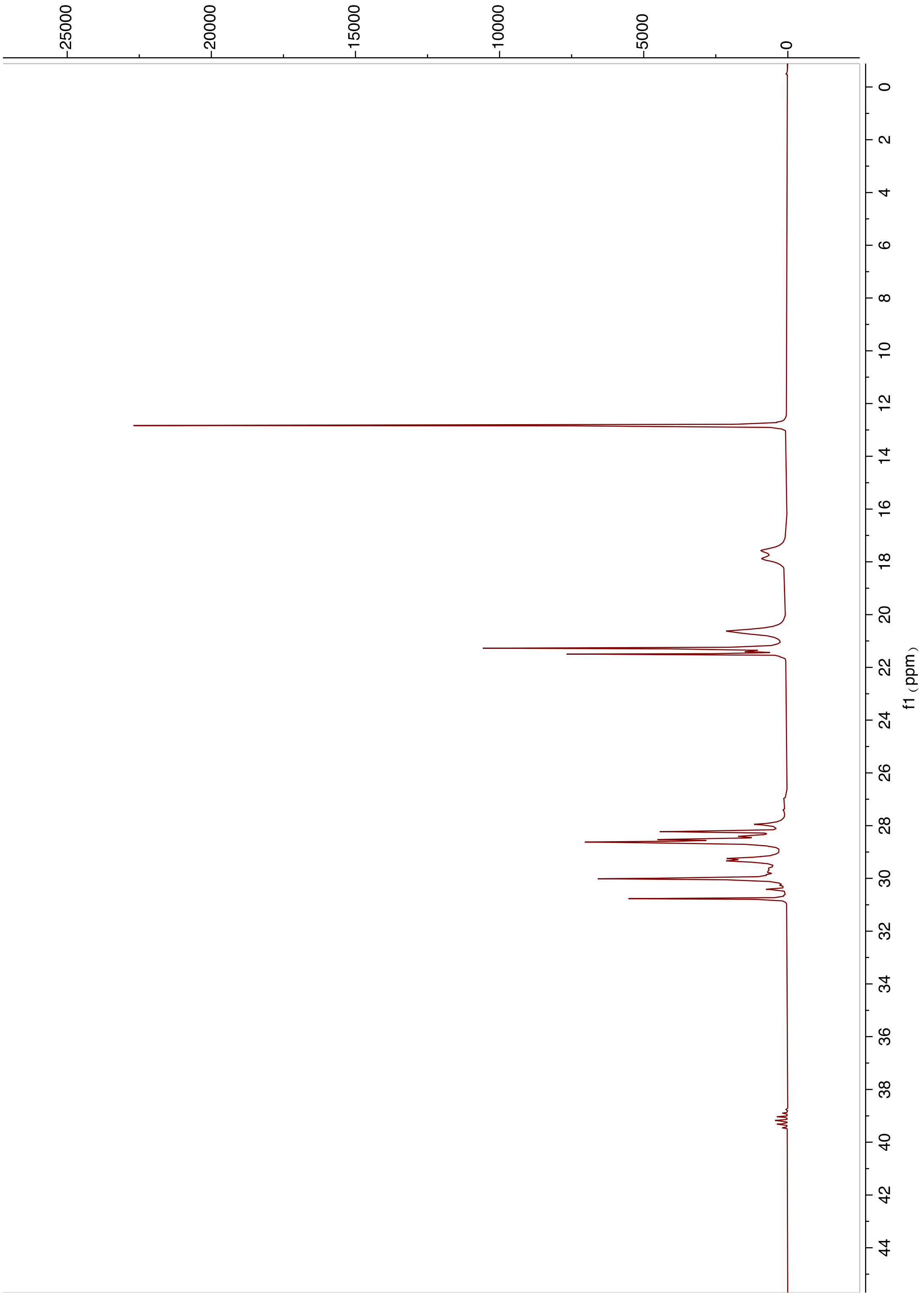

Supplementary Figure 20 ^{13}C NMR spectrum (d_6-DMSO, 150.90 MHz, 25 ˚C) of $[P_{666,14}][BH_4]$. Source data are provided as a Source Data file.

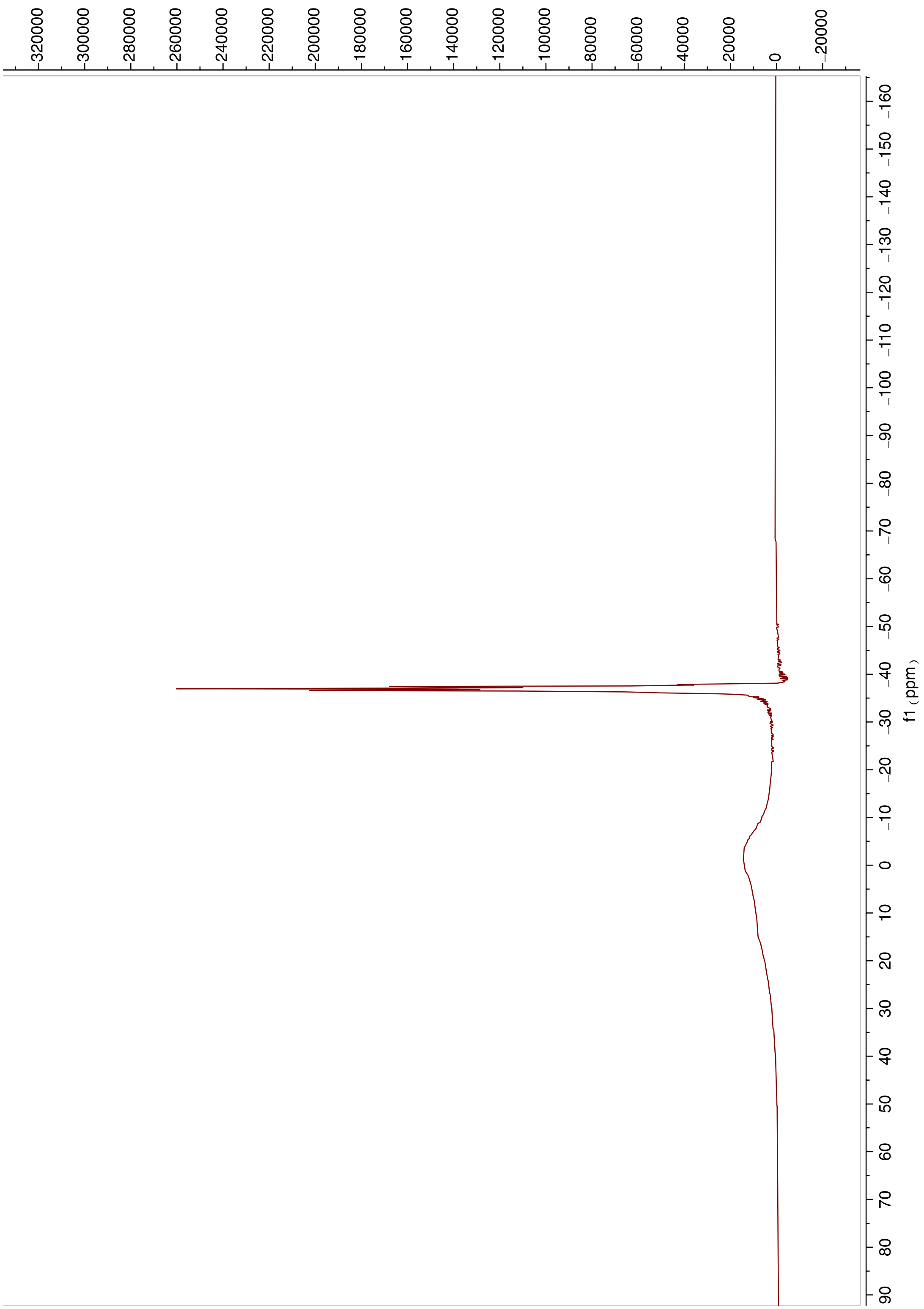

Supplementary Figure 21 ^{11}B NMR spectrum (d_6-DMSO, 64.48 MHz, 25 ˚C) of [$P_{666,14}$][BH_4]. Source data are provided as a Source Data file.

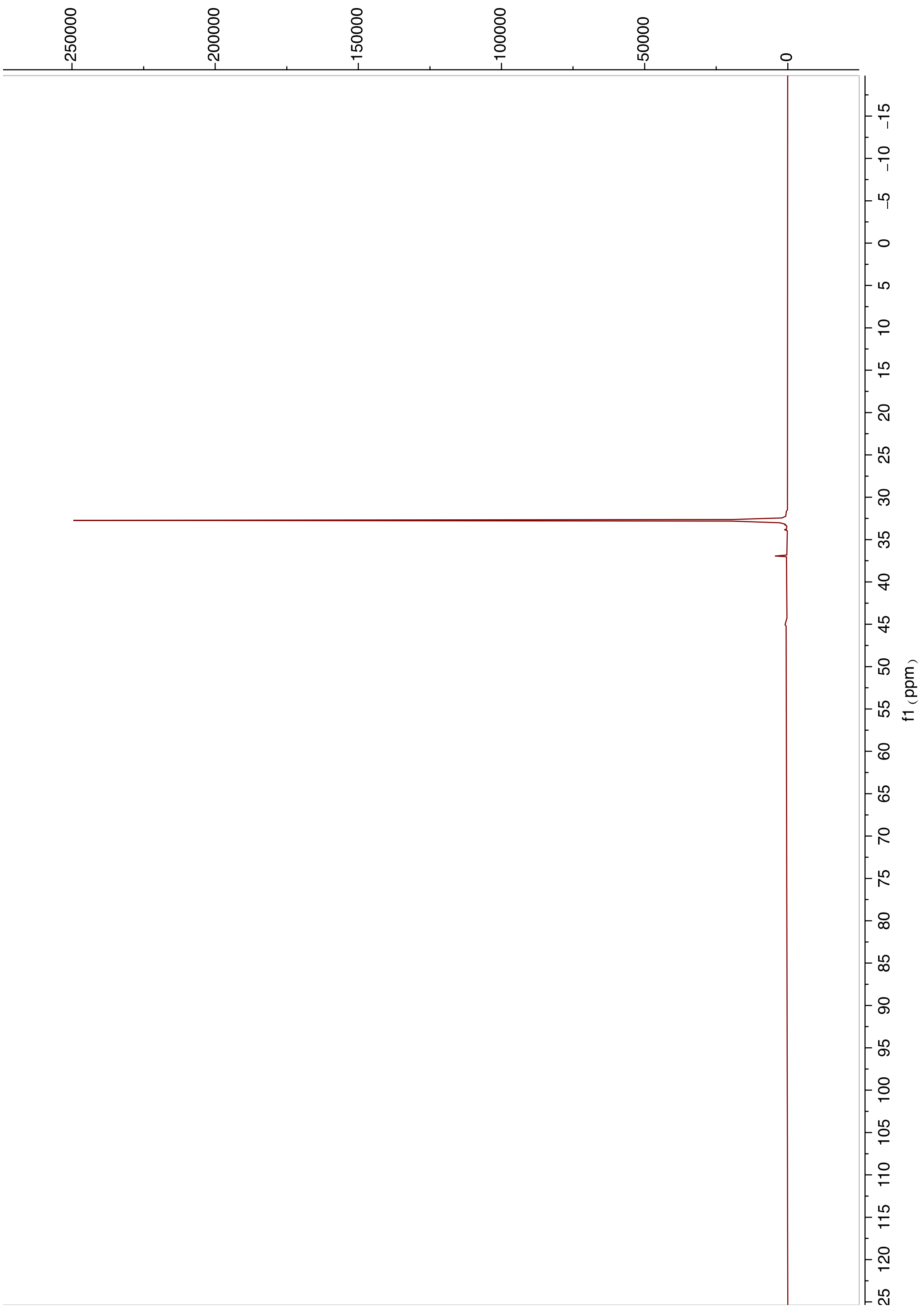

Supplementary Figure 22. ^{31}P NMR spectrum (d_6-DMSO, 242.94 MHz, 25 ˚C) of $[P_{666,14}][BH_4]$. Source data are provided as a Source Data file.

[$P_{666,14}$][BOB] was synthesized in a two-step synthesis, following a recently-reported, improved procedure[3].

Na[BOB]. Oxalic acid (0.03 mol eq.) and boric acid (0.01 mol eq.) were separately dissolved in water and then combined under constant stirring. Na_2CO_3 (0.5 mol eq.) was slowly added to the mixture with vigorous stirring. The turbid solution was heated in an oil bath at 120 °C and water collected by distillation until a dry white powder was obtained. The crude product was dispersed in hot acetonitrile at 60 °C and stirred for one hour. The white powder formed was isolated using vacuum filtration. The product was further washed with cold ethanol and the powder was dried overnight under high vacuum (12 h, 60 °C, 10^{-2} mbar). ^{13}C and ^{11}B NMR spectra of sodium bis(oxalate)borate (Na[BOB]) were recorded in $CDCl_3$.

$^{13}C\{^{1}H\}$NMR (150.90 MHz, $CDCl_3$) δ: 158.59.

^{11}B NMR (64.48 MHz, $CDCl_3$) δ: 7.35.

[$P_{666,14}$][BOB]. Trihexyl(tetradecyl)phosphonium chloride [$P_{666,14}$]Cl (0.01 mol eq.) and sodium bis(oxalato)borate (0.01 mol eq.) were mixed in 150 cm^3 of dichloromethane, DCM. The reaction mixture was stirred overnight at room temperature and then water was added under continuous stirring. The aqueous layer was separated, and the organic layer was collected and washed with 100 cm^3 deionized water (18.2 MΩ.cm). Subsequent washes were performed with solution of Na[BOB] in deionized water (18.2 MΩ.cm). Final three washes were performed with deionised water (18.2 MΩ.cm) water until no chloride could be detected with silver nitrate solution. Subsequently, DCM was removed *via* rotary evaporation (30 min, 35 °C) and the ionic liquid was dried overnight under high vacuum (12 h, 60 °C, 10^{-2} mbar). XRF analysis of [$P_{666,14}$][BOB] recorded a chloride content of 158 ppm with a lower detection limit (LLD) of 2.22 ppm. ^{1}H, ^{13}C and ^{31}P NMR spectra of the ionic liquid were recorded in $CDCl_3$.

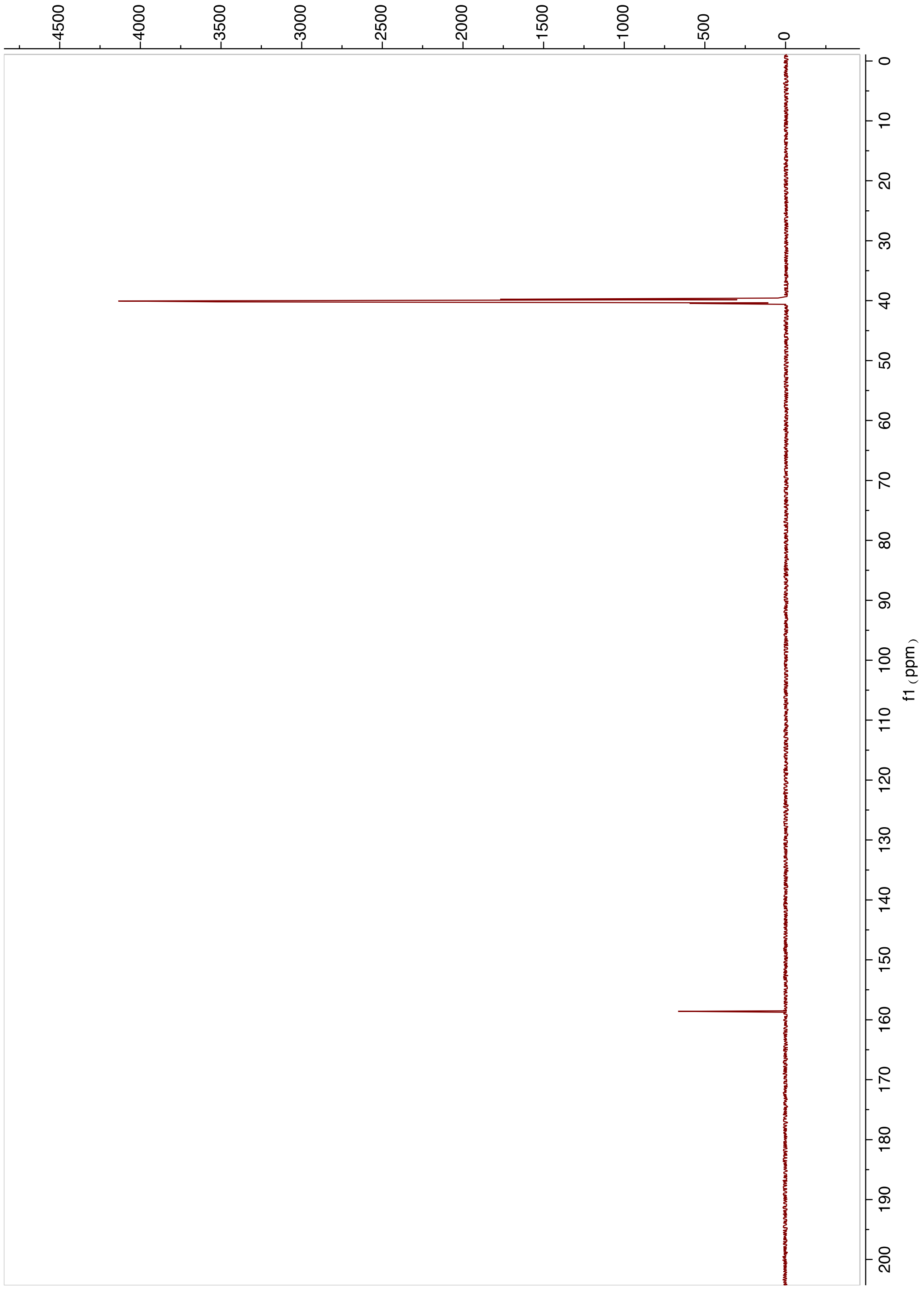

Supplementary Figure 23 ^{13}C NMR spectrum ($CDCl_3$, 150.90 MHz, 25 ˚C) of Na[BOB]. Source data are provided as a Source Data file.

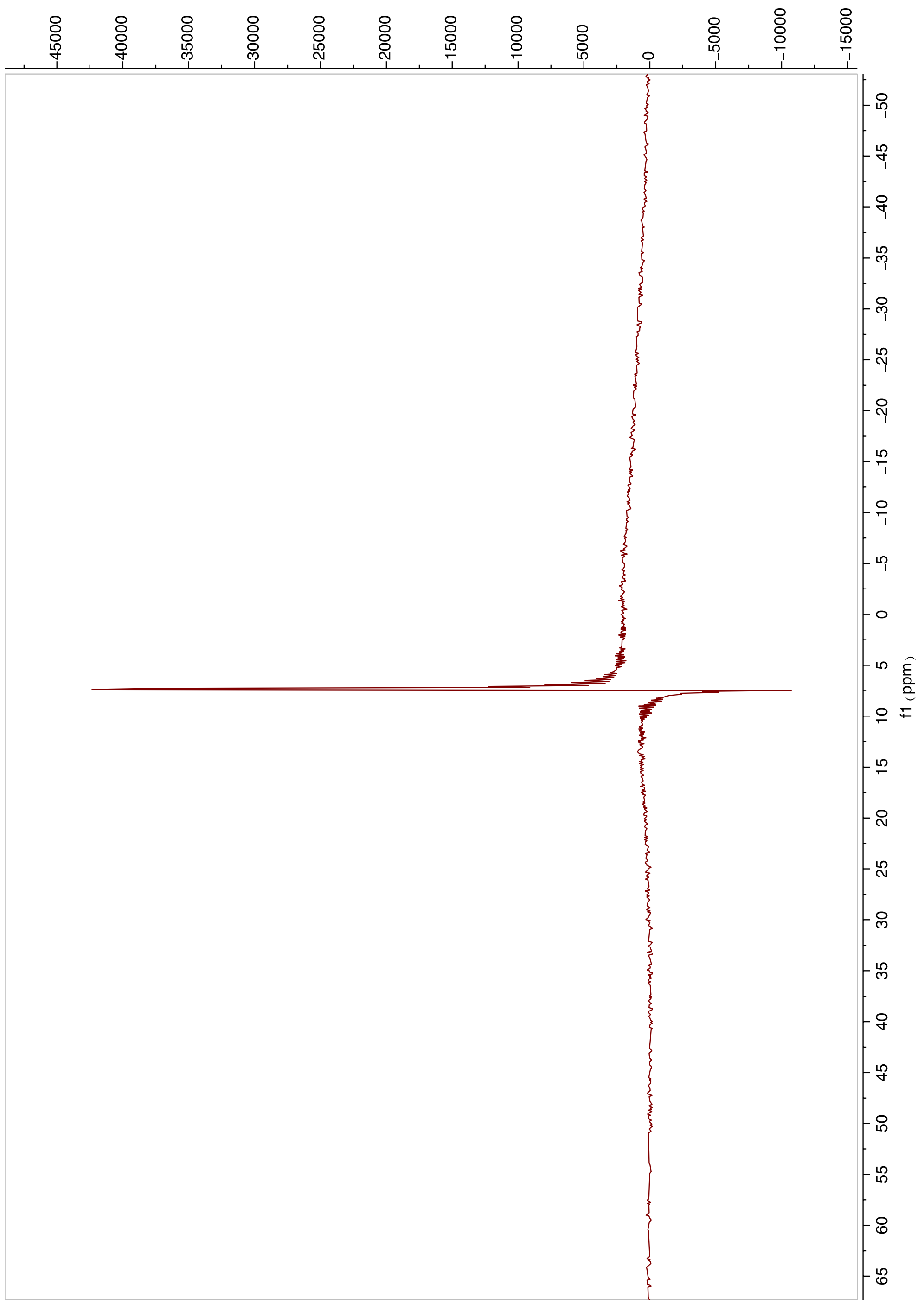

Supplementary Figure 24. ^{11}B NMR spectrum ($CDCl_3$, 64.48 MHz, 25 ˚C) of Na[BOB]. Source data are provided as a Source Data file.

^{1}H NMR (600.13 MHz, $CDCl_3$) δ: 0.86-0.91 (m, 12H), 1.21-1.31 (m, 18H), 1.31-1.35 (m, 14H), 1.44-1.51 (m, 8H), 1.51-1.58 (m, 8H), 2.09-2.21 (m, 8H).

$^{13}C\{^{1}H\}$NMR (150.90 MHz, $CDCl_3$) δ: 13.98 (s, P-$(CH_2)_5$-$\boldsymbol{CH_3}$), 14.23 (s, P-$(CH_2)_{13}$-$\boldsymbol{CH_3}$), 18.99 (d, $^{1}J_{C/P}$ = 186 Hz, P-$\boldsymbol{CH_2}$-$(CH_2)_4$-CH_3), 19.00 (d, $^{1}J_{C/P}$ = 186 Hz, P-$\boldsymbol{CH_2}$-$(CH_2)_{12}$-CH_3), 21.63 (s, P-$(CH_2)_{12}$-$\boldsymbol{CH_2}$-CH_3), 21.67 (s, P-$(CH_2)_{11}$-$\boldsymbol{CH_2}$-CH_2-CH_3), 21.71 (s, P-$(CH_2)_{10}$-$\boldsymbol{CH_2}$-$(CH_2)_2$-CH_3), 22.37 (s, P-$(CH_2)_9$-$\boldsymbol{CH_2}$-$(CH_2)_3$-CH_3), 22.80 (s, P-$(CH_2)_8$-$\boldsymbol{CH_2}$-$(CH_2)_4$-CH_3), 27.92 (s, P-$(CH_2)_7$-$\boldsymbol{CH_2}$-$(CH_2)_5$-CH_3), 28.35 (s, P-$(CH_2)_6$-$\boldsymbol{CH_2}$-$(CH_2)_6$-CH_3), 28.94 (s, P-$(CH_2)_5$-$\boldsymbol{CH_2}$-$(CH_2)_7$-CH_3), 29.47 (d, $^{2}J_{C/P}$ = 72 Hz, P-CH_2-$\boldsymbol{CH_2}$-$(CH_2)_3$-CH_3), 29.65 (d, $^{2}J_{C/P}$ = 72 Hz, P-CH_2-$\boldsymbol{CH_2}$-$(CH_2)_{11}$-CH_3), 29.75 (s, P-$(CH_2)_4$-$\boldsymbol{CH_2}$-CH_3), 29.79 (s, P-$(CH_2)_4$-$\boldsymbol{CH_2}$-$(CH_2)_8$-CH_3), 30.44 (d, $^{3}J_{C/P}$ = 60 Hz, P-$(CH_2)_2$-$\boldsymbol{CH_2}$-$(CH_2)_2$-CH_3), 30.79 (d, $^{3}J_{C/P}$ = 60 Hz, P-$(CH_2)_2$-$\boldsymbol{CH_2}$-$(CH_2)_{10}$-CH_3), 31.01 (s, P-$(CH_2)_3$-$\boldsymbol{CH_2}$-$(CH_2)_9$-CH_3), 32.03 (s, P-$(CH_2)_3$-$\boldsymbol{CH_2}$-CH_2-CH_3), 159.03 (s, B($\boldsymbol{C_2}O_4)_2$).

^{11}B NMR (64.48 MHz, $CDCl_3$) δ: 7.66.

$^{31}P\{^{1}H\}$ NMR (242.94 MHz, $CDCl_3$) δ: 33.18.

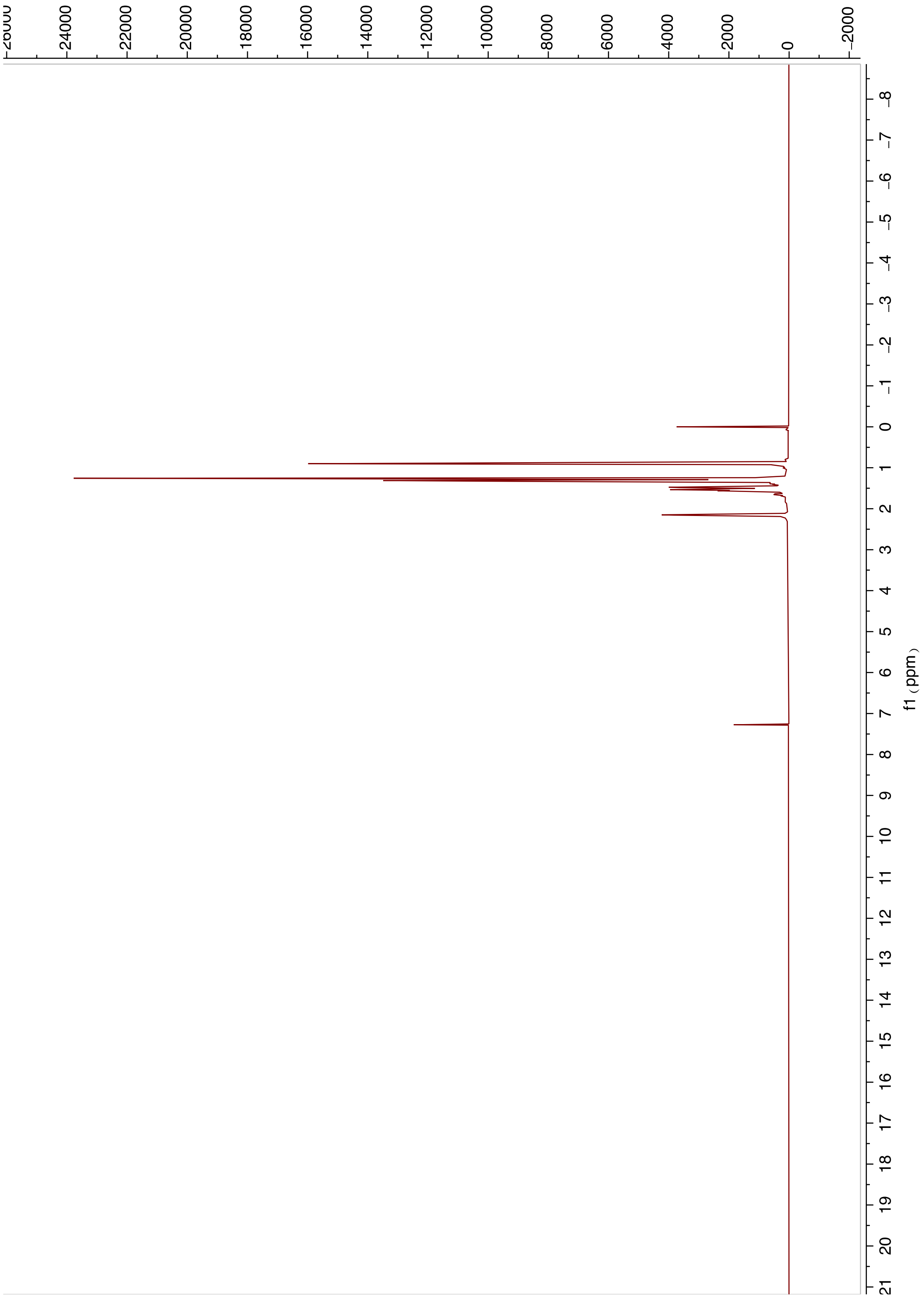

Supplementary Figure 25 ^{1}H NMR spectrum ($CDCl_3$, 600.13 MHz, 25 ˚C) of [$P_{666,14}$][BOB]. Source data are provided as a Source Data file.

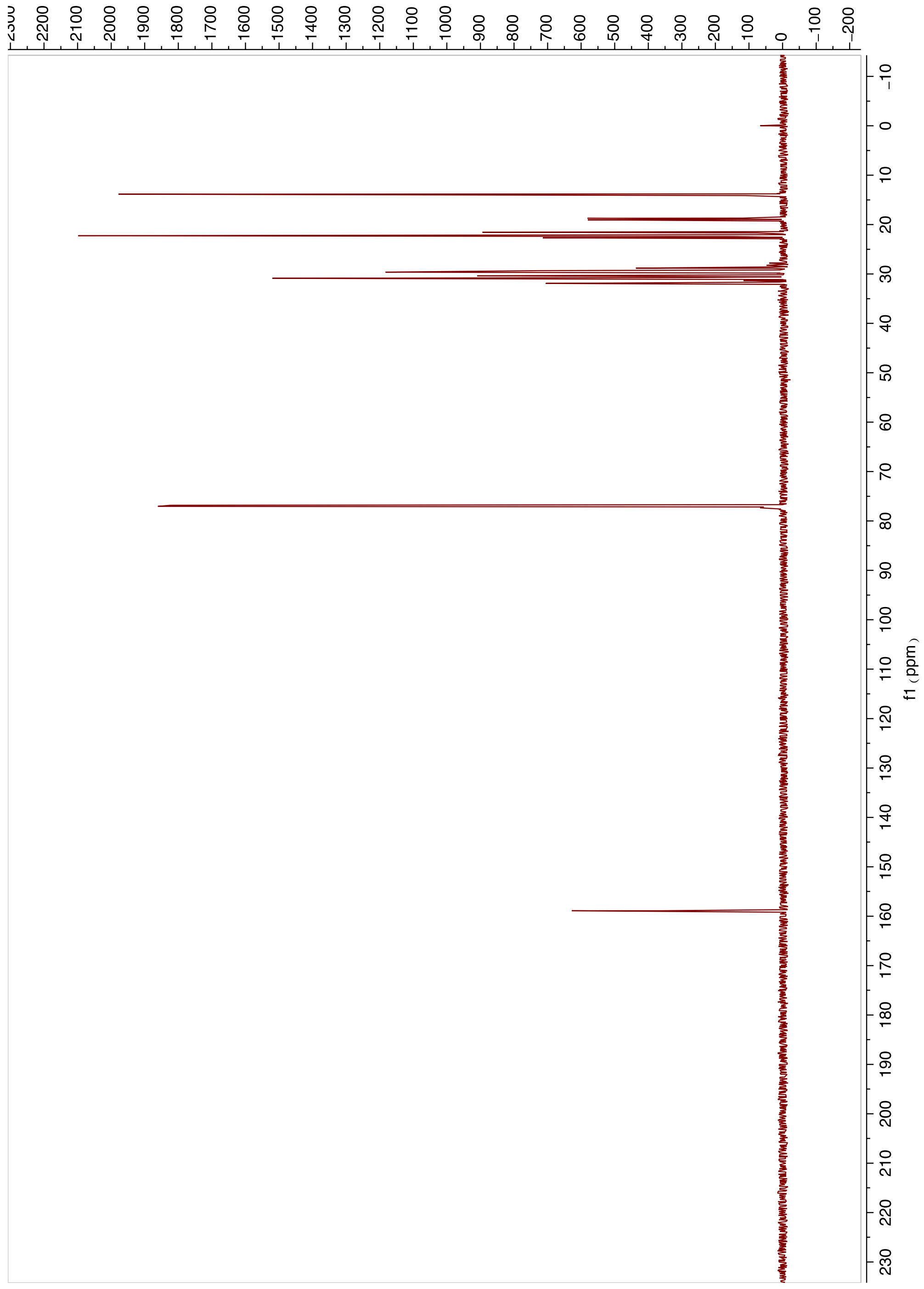

Supplementary Figure 26 ^{13}C NMR spectrum ($CDCl_3$, 150.90 MHz, 25 ˚C) of [$P_{666,14}$][BOB]. Source data are provided as a Source Data file.

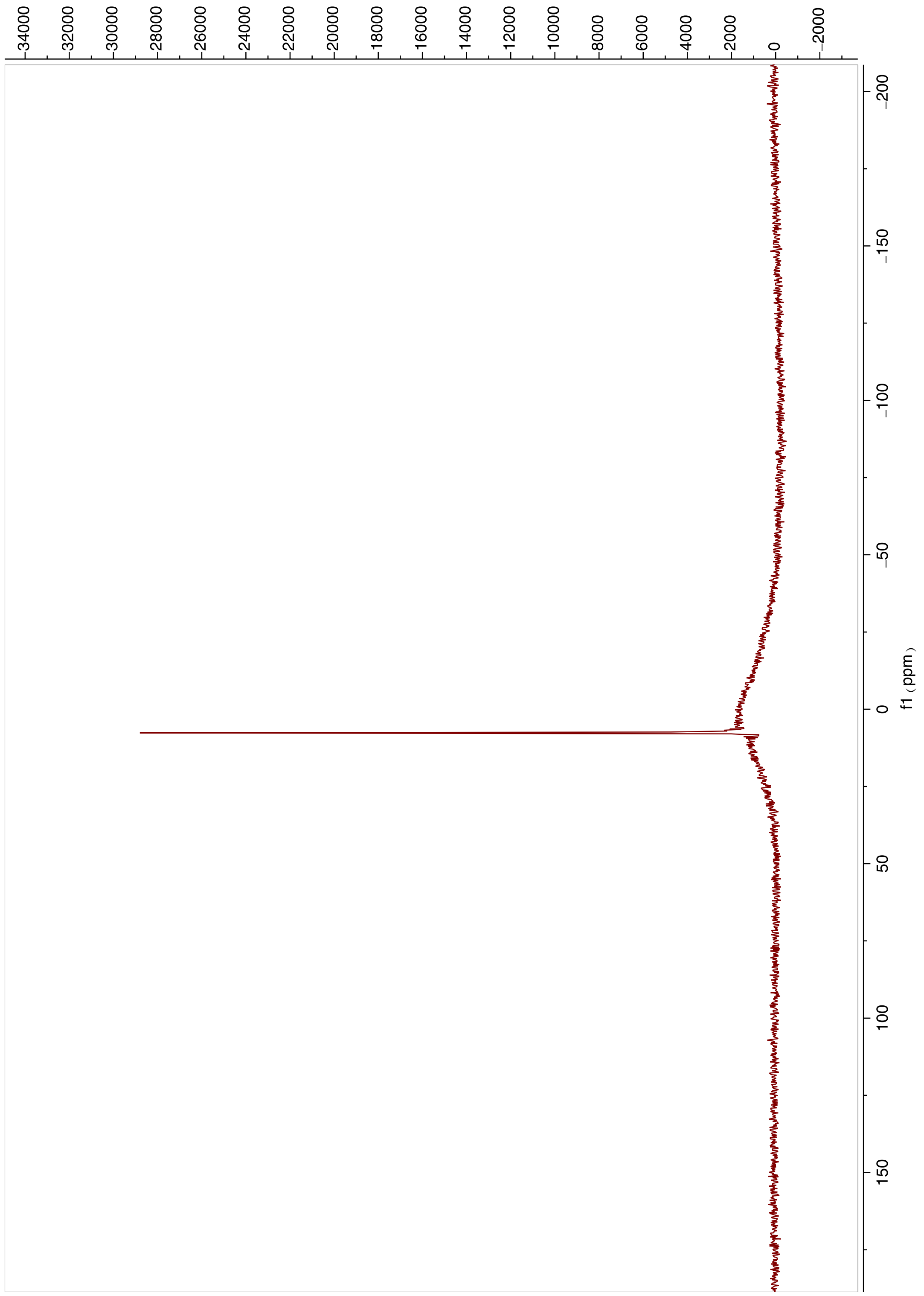

Supplementary Figure 27 ^{11}B NMR spectrum ($CDCl_3$, 64.48 MHz, 25 ˚C) of [$P_{666,14}$][BOB]. Source data are provided as a Source Data file.

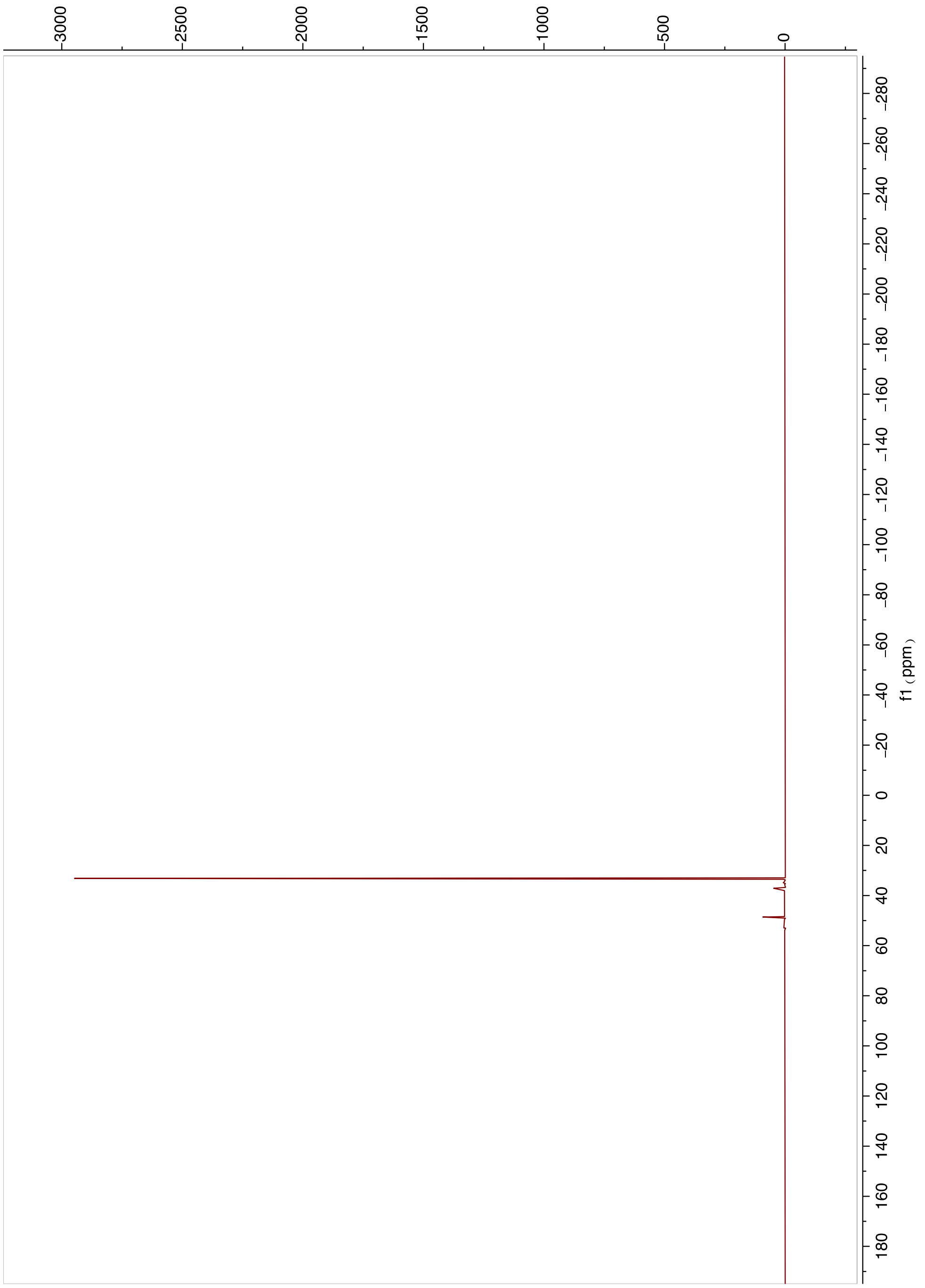

Supplementary Figure 28. ^{31}P NMR spectrum ($CDCl_3$, 242.94 MHz, 25 ˚C) of [$P_{666,14}$][BOB]. Source data are provided as a Source Data file.

Prior to the measurements, the samples were dried under vacuum at 90 °C for 24 h. The water content detected using the Karl Fischer method, was around 500 and 300 ppm before and after drying, respectively. The one exception is [$P_{666,14}$][BH_4] with the water content of 3% after drying.

Supplementary Note 1

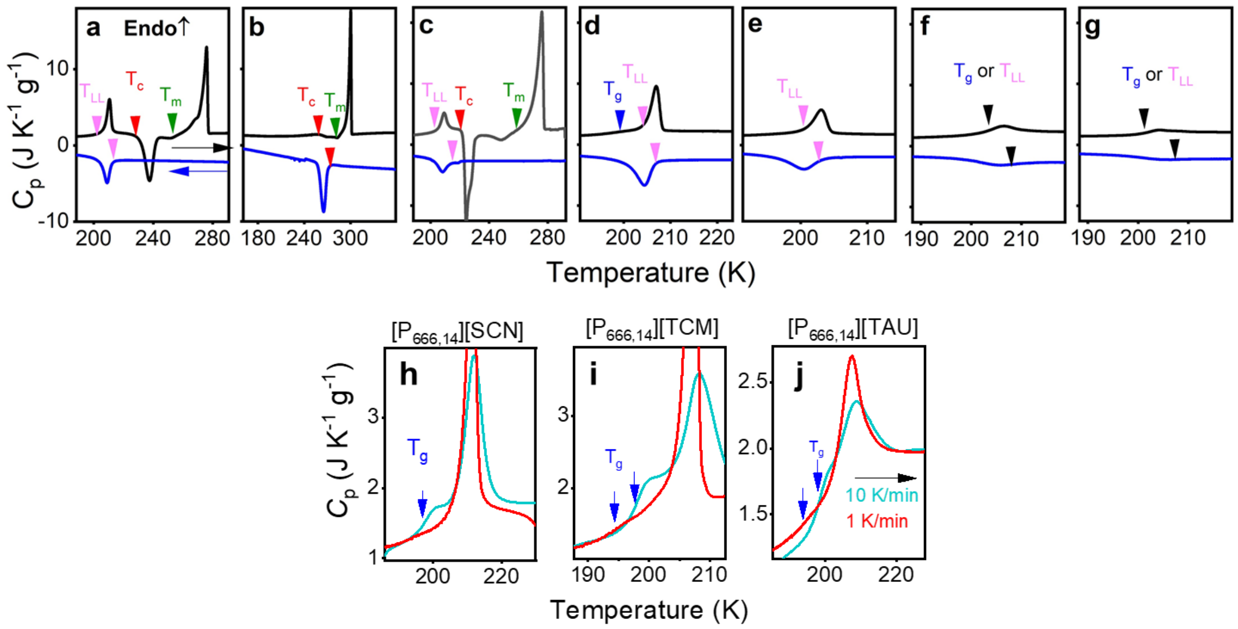

Supplementary Figure 29 Differential scanning calorimetry (DSC) traces of $[P_{666,14}]^+$-based ILs. The DSC data collected on cooling (blue curve) and subsequent heating (black curve) with the rate of 1 K·min^{-1} of **a** $[P_{666,14}]$[SCN], **b** $[P_{666,14}][BF_4]$, **c** $[P_{666,14}][BH_4]$, **d** $[P_{666,14}]$[TCM], **e** $[P_{666,14}]$[TFSI], **f** $[P_{666,14}]$[TAU], **g** $[P_{666,14}]$[BOB]. Arrows indicate the onset of: LLT (pink one), melting point (green one), cold crystallization (red one) and T_g (blue one). Panels **h**, **i** and **j** shows DSC thermograms obtained on heating of aged glass with the rate of 1 K per min (red curve) and 10 K per min (turquoise curve). T_g shifts left on a slower scanning rate as indicated by blue arrows; however, at the same time, a slow release of enthalpy occurs and overshoot peak is no longer visible. Source data are provided as a Source Data file

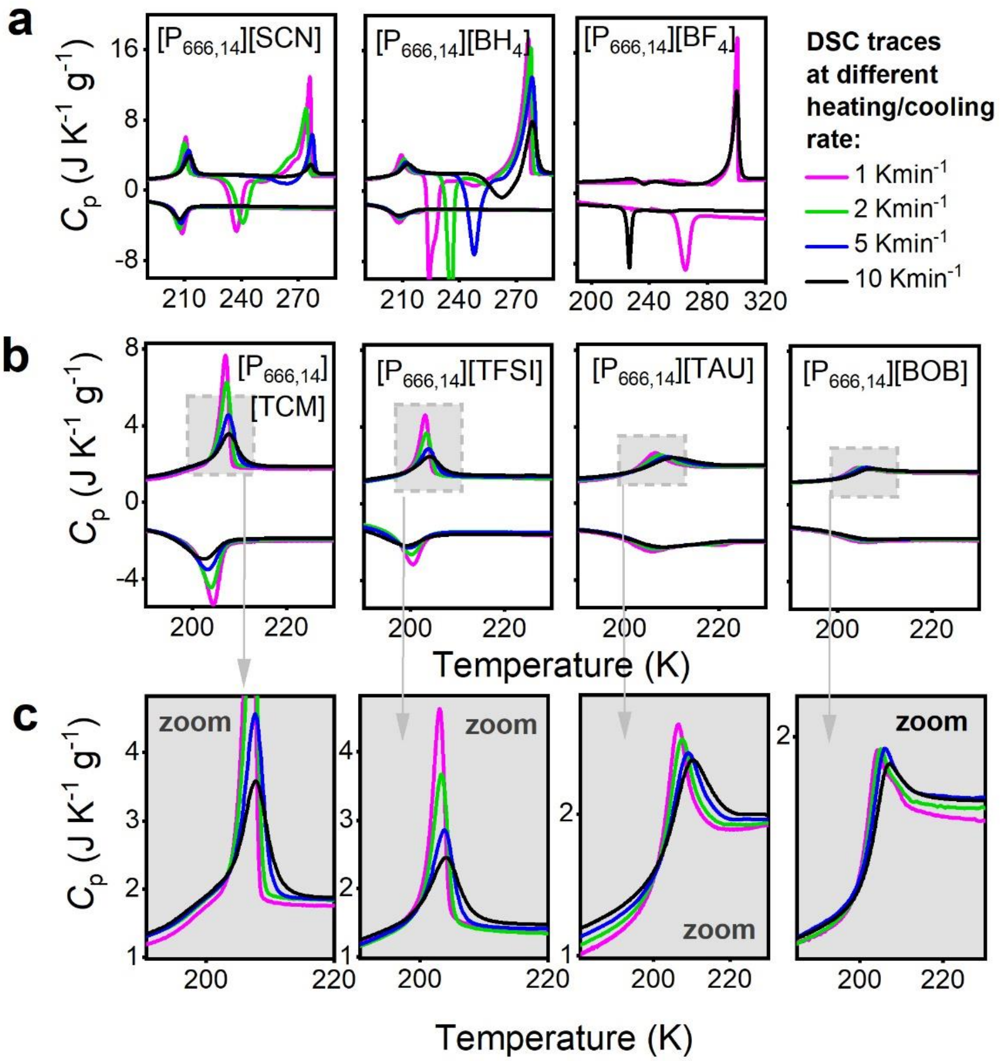

Supplementary Figure 30. a and **b** Heat capacity measured on different heating and cooling rate:10 (black), 5 (blue), 2 (green), and 1 (pink) K per min for [$P_{666,14}$]-based ionic liquids (the name of IL is described in the panel). Panel **c** presents the zoom of data presented in panel **b**. Source data are provided as a Source Data file.

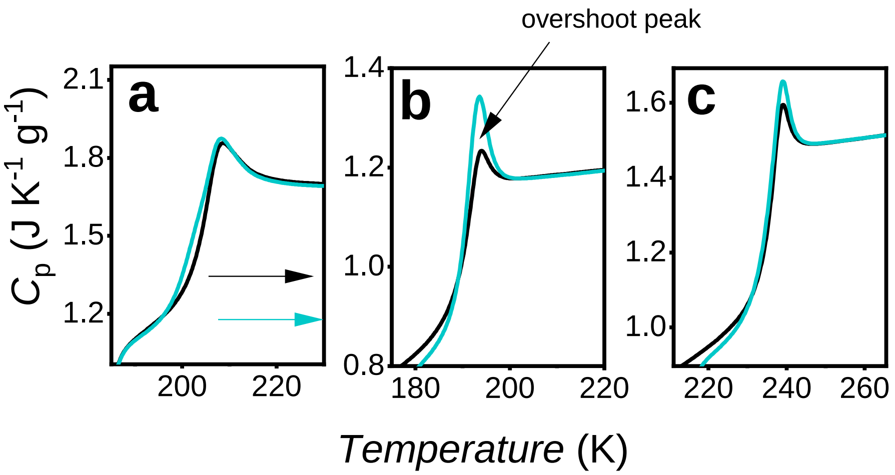

Supplementary Figure 31 Comparison between DSC traces obtained during the standard heating with rate 10 K/min (black curved) and after the 6 hours-aging process performed in the glassy state (turquoise curve) for **a** [$P_{666,14}$][BOB], **b** [BMIm][BETI] and **c** [BMP][BOB]. In the latter two cases, clear overshoot peak of aged glass is visible. Source data are provided as a Source Data file.

Supplementary Note 2

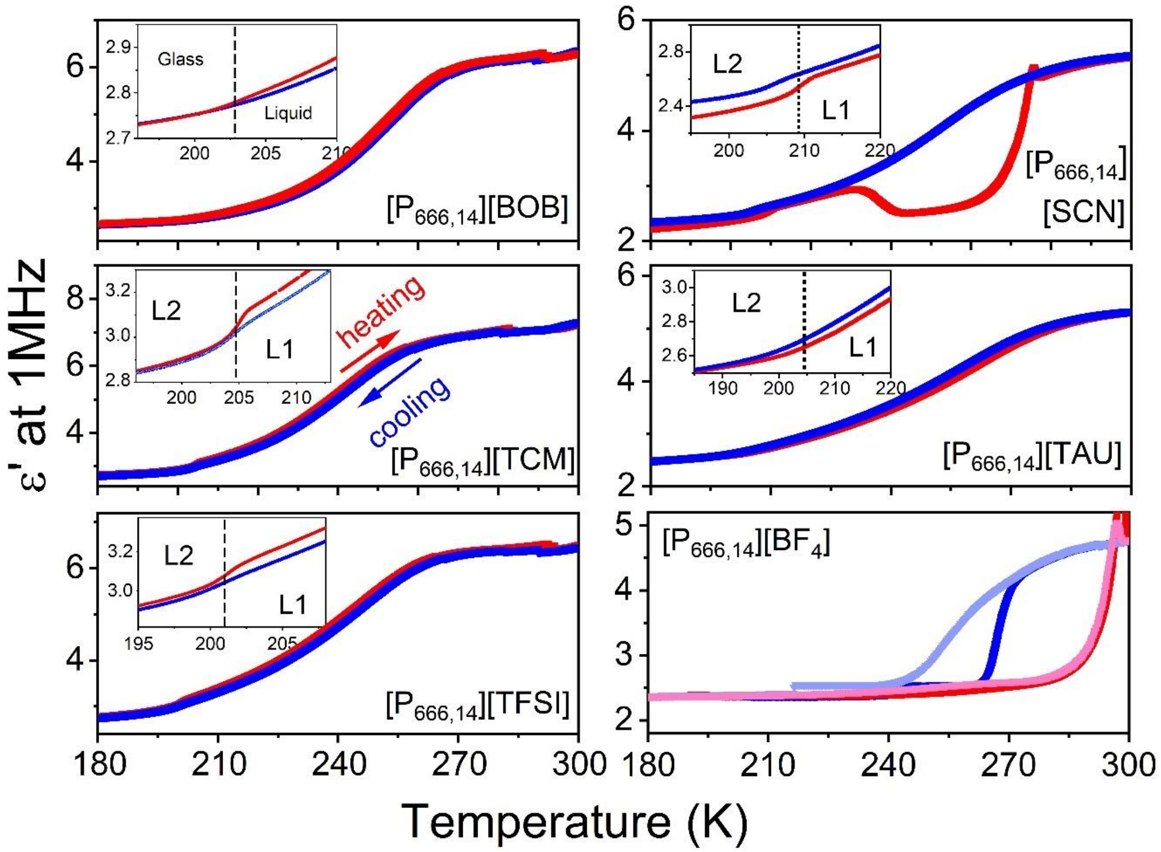

Supplementary Figure 32. The change of ε' as a function of temperature at fixed frequency (f=1MHz) during cooling (blue) and heating (red) with a rate of 1 K per min for [P_{66614}] [BOB], [P_{66614}] [TCM], [P_{66614}] [TFSI], [P_{66614}] [SCN], [P_{66614}] [Tau] and [P_{66614}] [BF_4]. The light blue and light red curves in the graph of [P_{66614}] [BF_4] denote the cooling and heating processes with a rate of 5 K per min, respectively. Source data are provided as a Source Data file.

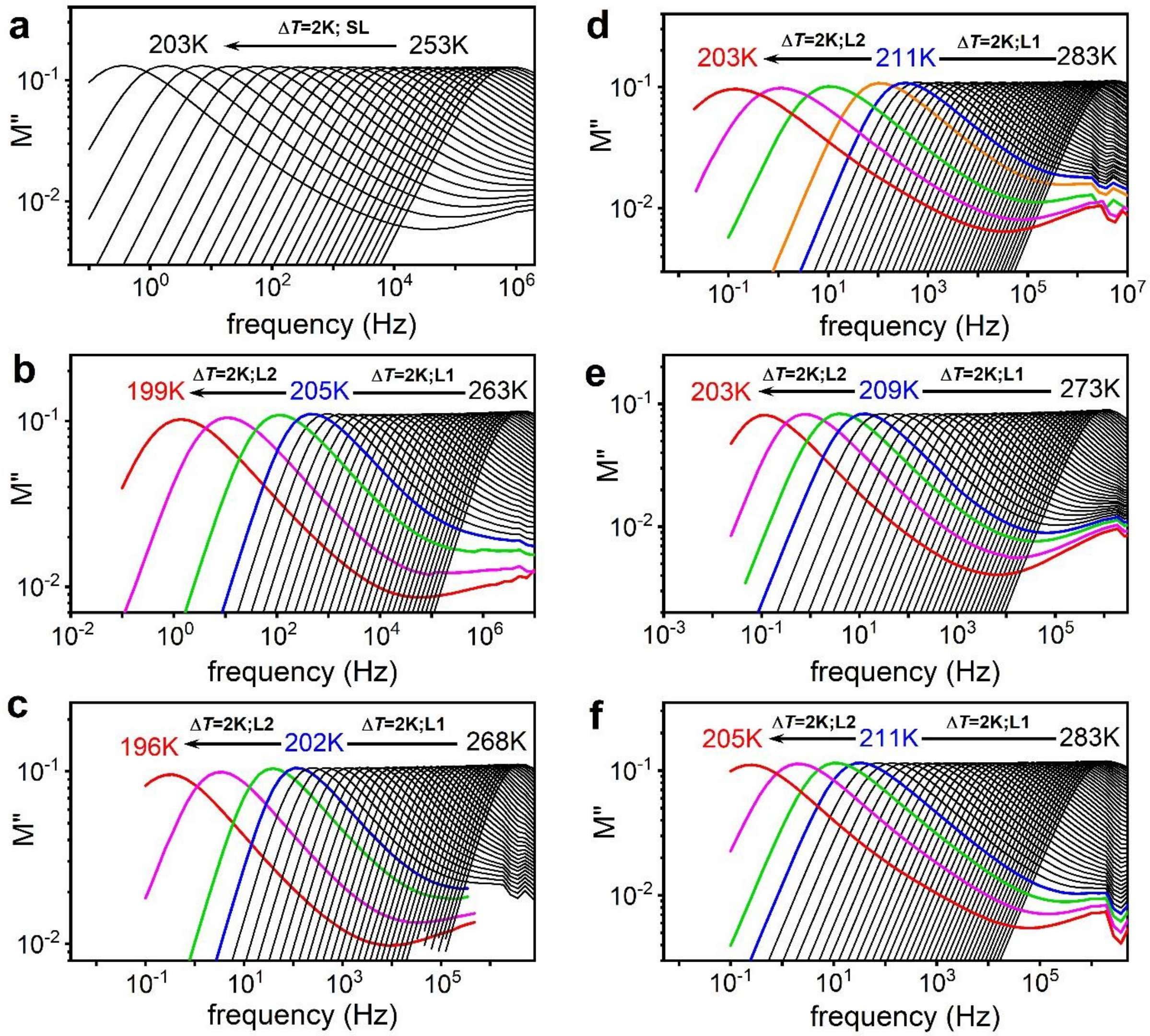

Supplementary Figure 33. All the dielectric modulus spectra collected for [P_{66614}]-based IL at ambient pressure conditions. **a** [$P_{666,14}$][BOB], **b** [$P_{666,14}$][TCM], **c** [$P_{666,14}$][TFSI], **d** [$P_{666,14}$][SCN], **e** [$P_{666,14}$][TAU], **f** [$P_{666,14}$][BH_4]. Source data are provided as a Source Data file.

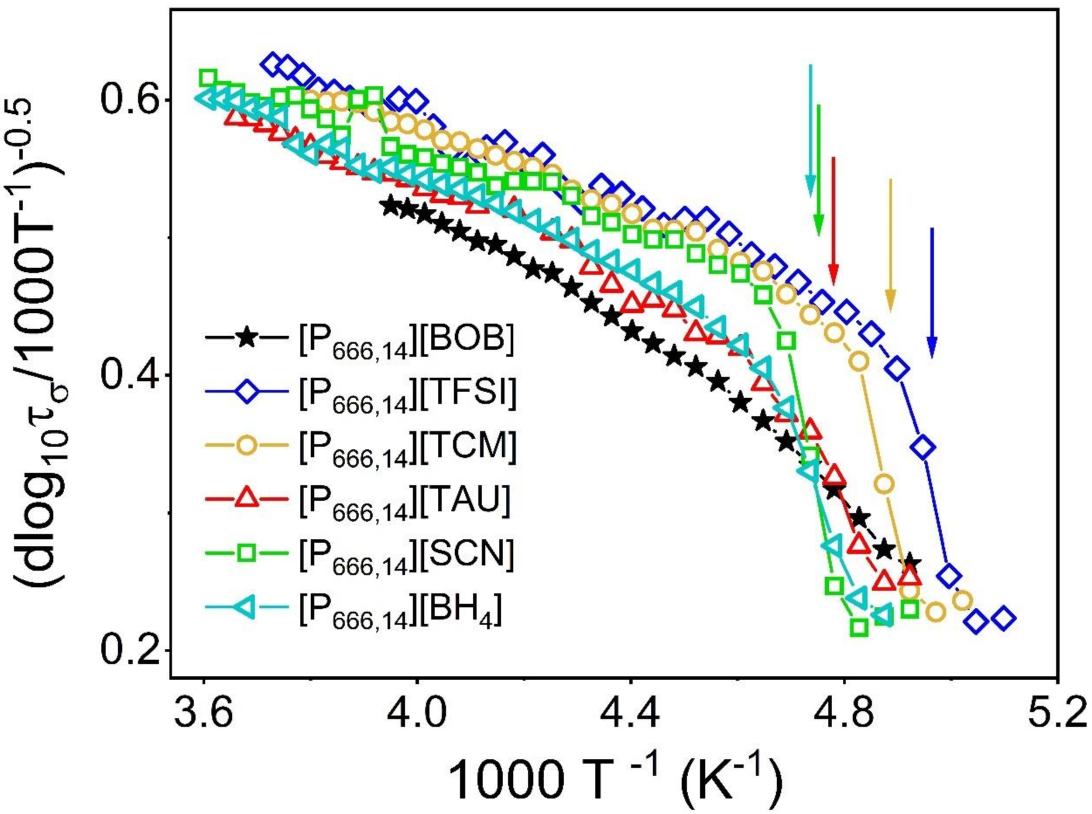

Supplementary Figure 34. Stickel analysis of studied samples. Arrows indicate the LLT. Source data are provided as a Source Data file.

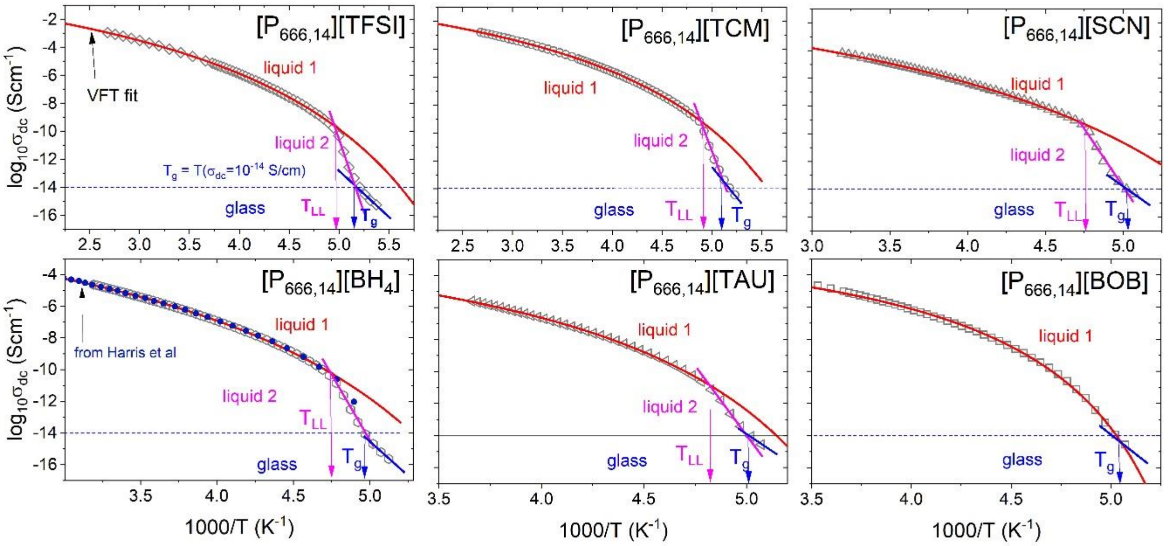

Supplementary Figure 35. The dc-conductivity of [$P_{666,14}$]-based ionic liquids as a function of inverse temperature. The solid circles for [$P_{666,14}$][BH_4] represent the data taken from ref. [4]. T_{LL} denotes temperature of liquid-liquid transition. Source data are provided as a Source Data file.

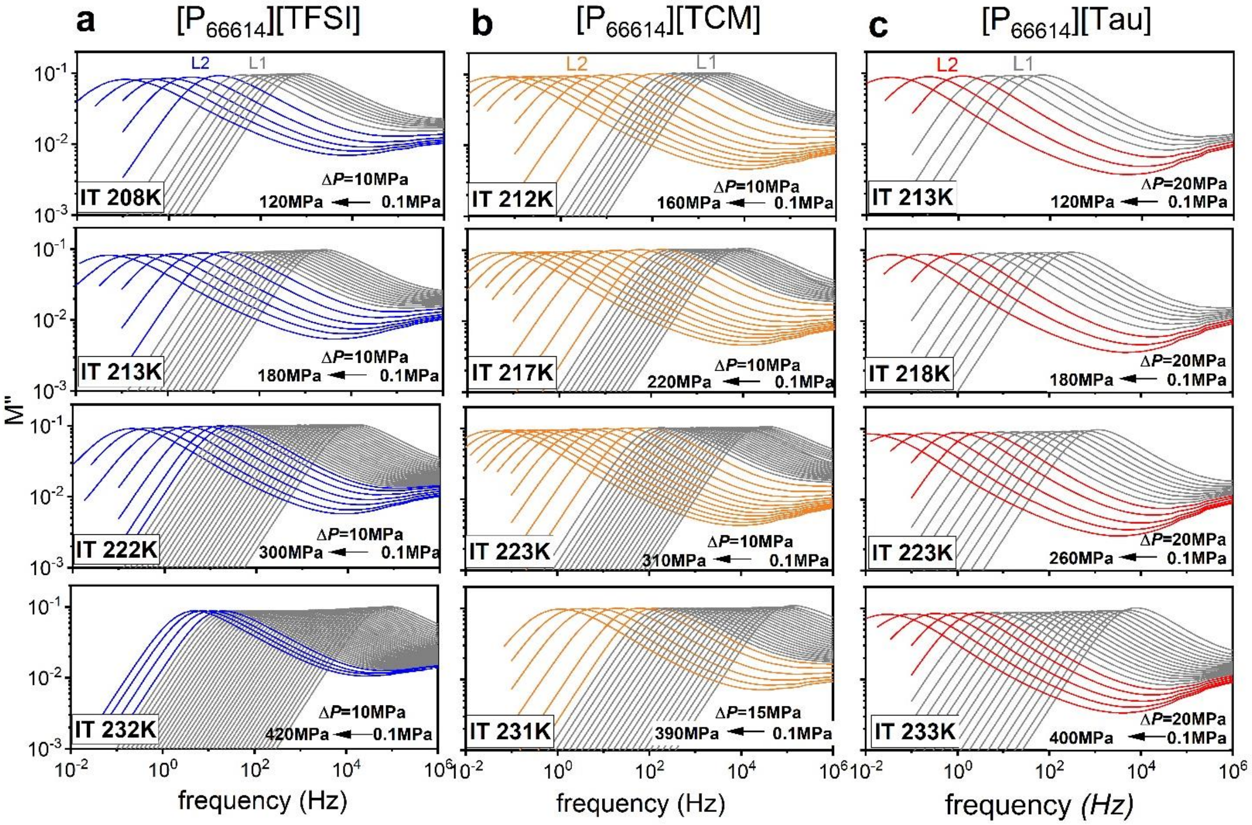

Supplementary Figure 36. Dielectric modulus data recorded at high-pressure conditions and four different temperatures for **a** [$P_{666,14}$][[TFSI], **b** [$P_{666,14}$][[TCM] and **c** [$P_{666,14}$][[TAU]. IT denotes temperature of isotherm. The data collected in liquid 1 (L1) are marked in grey, while data collected in liquid 2 (L2) are marked in color. Source data are provided as a Source Data file.

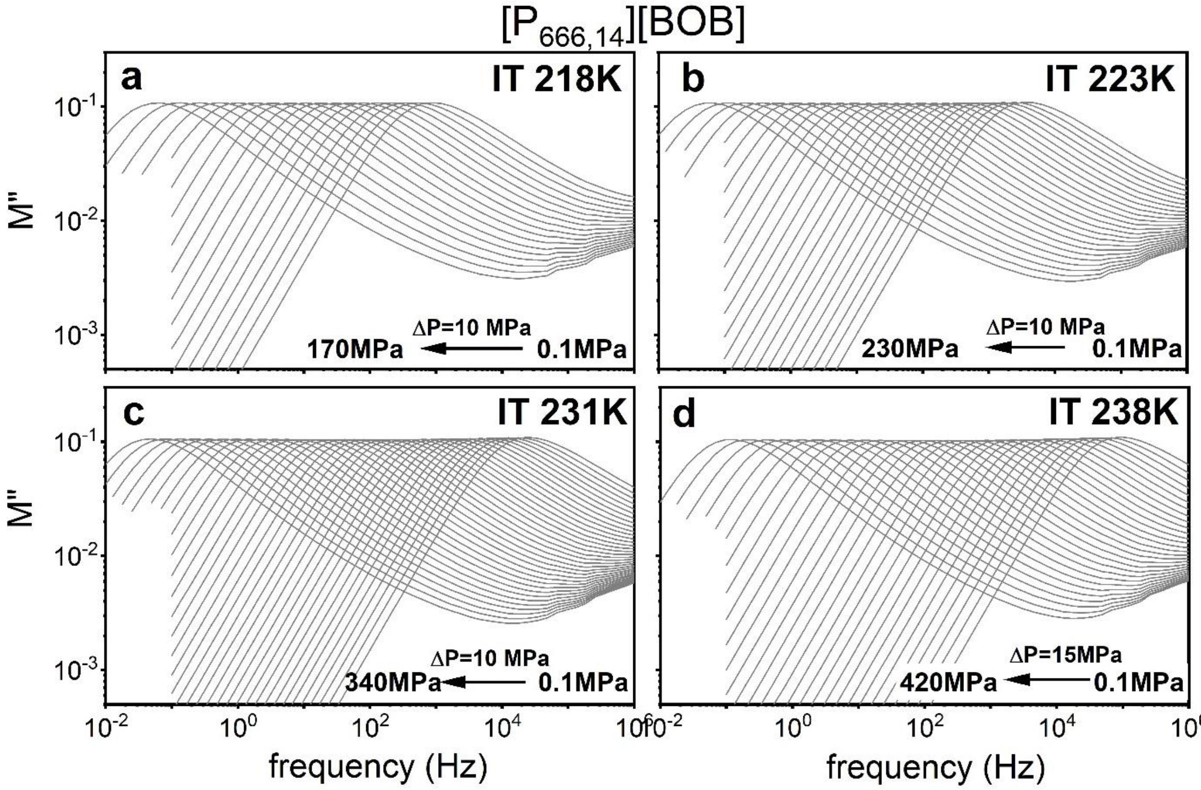

Supplementary Figure 37. Dielectric modulus data of [$P_{666,14}$][BOB] recorded at high-pressure conditions at **a** 218 K, **b** 223 K, **c** 231 K, **d** 238 K. IT means isotherm. Source data are provided as a Source Data file.

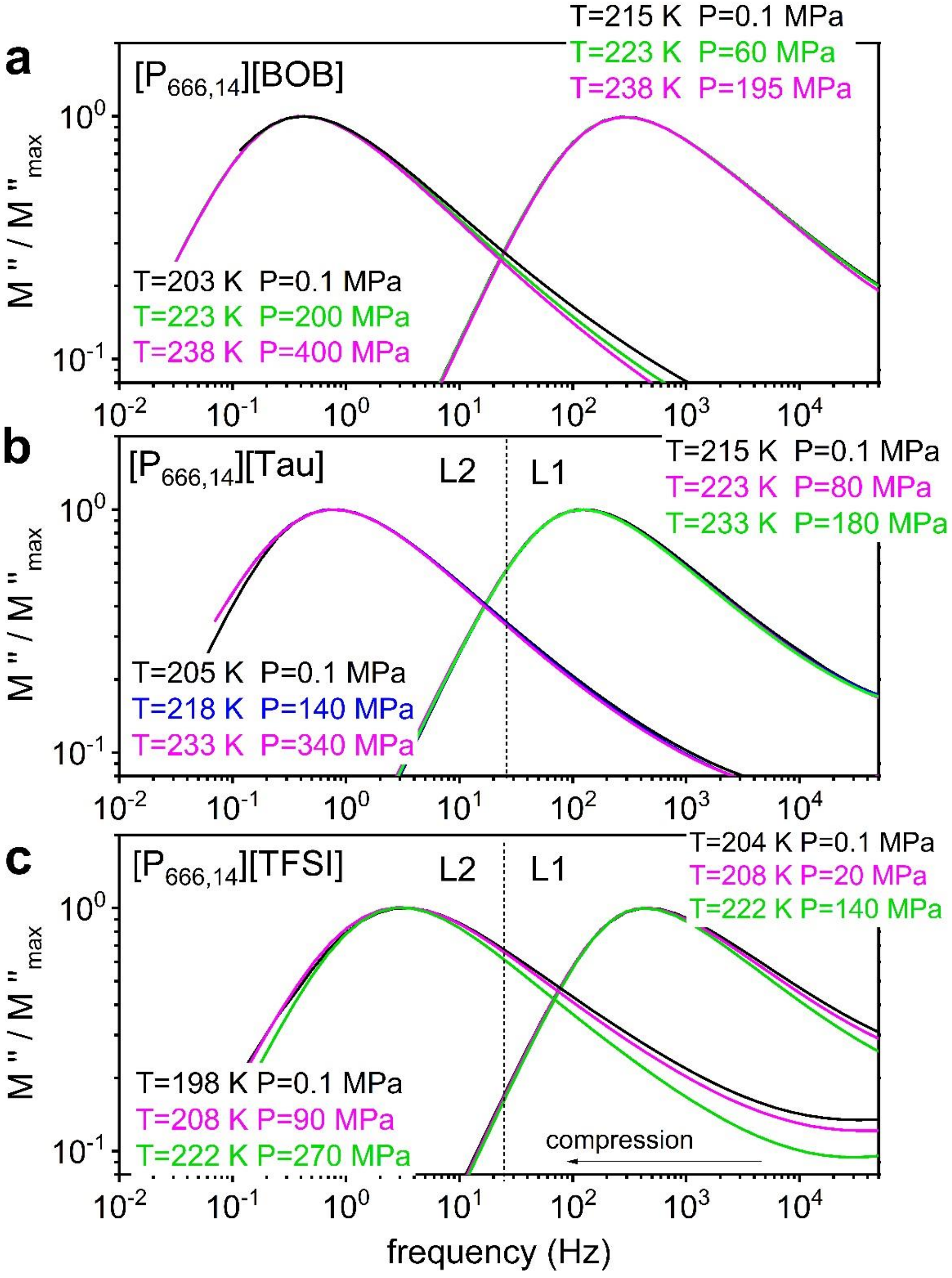

Supplementary Figure 38. The representative *M*''(*f*) spectra of **a** [$P_{666,14}$][BOB], **b** [$P_{666,14}$][TAU] and **c** [$P_{666,14}$][TFSI] recorded at various *T*-*P* conditions however the same τ_σ superimposed to each other at two different frequencies in liquid 1 (L1) and liquid 2 (L2). Source data are provided as a Source Data file.

Supplementary Note 3

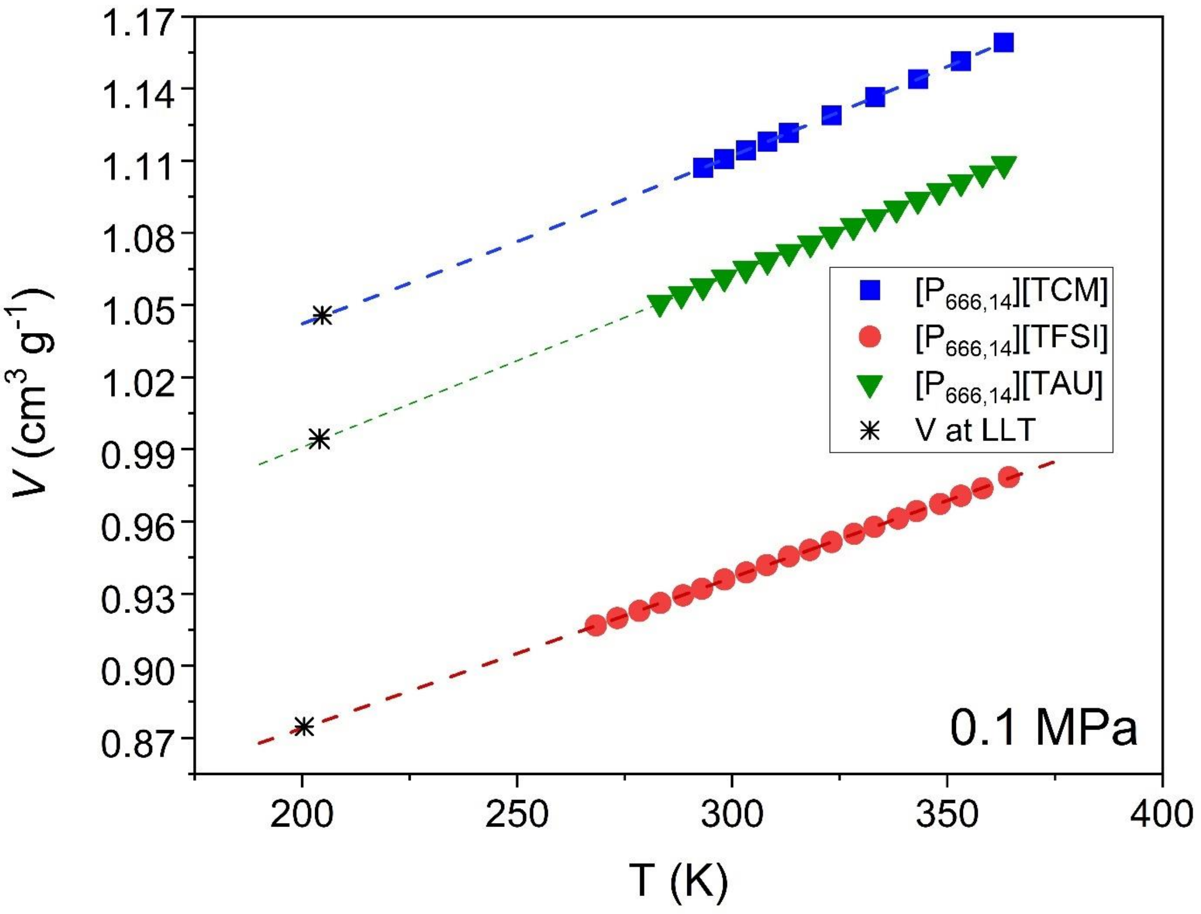

Supplementary Figure 39. Temperature dependence of specific volume for $[P_{666,14}]$[TCM], $[P_{666,14}]$[TAU] and $[P_{666,14}]$[TFSI]. The data for $[P_{666,14}]$[TFSI] were taken from ref. 5. Source data are provided as a Source Data file.

Supplementary references

[1] Ferguson, J. L. , Holbrey, J. D., Plechkova, S. Ng, N. V., Seddon, K. R., Tomaszowska, A. A. Wassell, D.F. A greener, halide-free approach to ionic liquid synthesis *Pure Appl. Chem*., **84**, 723–744 (2012)

[2] Goodrich, B.F., de la Fuente, J.C., Gurkan, B.E., Lopez, Z. K., Price, E.A., Huang, Y., Brennecke, J.F. Effect of Water and Temperature on Absorption of CO2 by Amine-Functionalized Anion-Tethered Ionic Liquids *J. Phys. Chem. B*, **115**, 29, 9140–9150 (2011)

[3] Shimpi, M.R., Rohlmann, P., Shah, F.U., Glavatskih, S. Antzutkin, O.N. Transition anionic complex in trihexyl(tetradecyl)phosphonium-bis(oxalato)borate ionic liquid – revisited P*hys. Chem. Chem. Phys*., **23**, 6190-6203 (2021)

[4] Harris, M.A., Kinsey, T., Wagle, D.V., Baker, G.A. & Sangoro, A. Evidence of a liquid–liquid transition in a glass-forming ionic liquid, *PNAS*, **118**, 11 (2021)

[5] Klomfar, J.; Souckova, M.; Patek, J. Low Temperature Densities from (218 to 364) K and up to 50 MPa in Pressure and Surface Tension for Trihexyl(tetradecyl)phosphonium Bis(trifluoromethylsulfonyl)imide and Dicyanamide and 1-Hexyl-3-methylimidazolium Hexafluorophosphate *J. Chem. Eng. Data* **59**, 7, 2263-2274, (2014)
