# Supplementary material for: Pressure-induced liquid-liquid transition in a family of ionic materials
Source: Nat Commun. 2022 Mar 15;13:1342. doi: 10.1038/s41467-022-29021-0 (PMC8924164; doi:10.1038/s41467-022-29021-0)
Supplement: Supplementary file 2 — Description of Additional Supplementary Files [file 41467_2022_29021_MOESM2_ESM.docx]

**Supplementary Data legends**

**Pressure-induced liquid-liquid transition in a family of ionic materials**Z. Wojnarowska et al.

File Name: Supplementary Data 1
Description: Edited ChemSketch structures of cation and anions.

File name: Source Data

Description: The file contains all data presented in the main manuscript and supplementary information. The numerical dielectric, calorimetric and NMR data are available in excel files.
